# Are the Growth Standards of the World Health Organization Valid for Spanish Children? The SONEV Study

**DOI:** 10.3389/fped.2021.700748

**Published:** 2021-08-16

**Authors:** Marcelino Pérez-Bermejo, Luisa Alcalá-Dávalos, Javier Pérez-Murillo, Maria Ester Legidos-García, Maria Teresa Murillo-Llorente

**Affiliations:** SONEV Research Group (Overweight, Obesity, Nutrition and Lifestyles), School of Medicine and Health Sciences, Catholic University of Valencia San Vicente Mártir, Valencia, Spain

**Keywords:** child, adolescent, anthropometry, growth charts, cubic-spline, percentiles, z-scores

## Abstract

**Background:** The use of different growth tables to assess the population's nutritional status has given rise to a series of limitations arising from the lack of consensus and uniform methodological criteria. This leads to a disparity of results that prevent an accurate and reliable diagnosis of whether a child is overweight or obese.

**Objective:** The purpose of this study was to develop growth references for weight, height, and body mass index for Eastern-Spanish children from 6 to 16 years of age.

**Methods:** The final sample used to fit the growth curves was made up of 1,102 observations. The 2007 WHO curves are currently used for Child Health Service Cards. Therefore, to make the comparison of the internal values obtained as realistic as possible, the same construction method has been used for the internal curves, modeling age as a continuous variable and simultaneously adjusting the curves, smoothing them using cubic splines and further smoothing the edge effects by means of data extending above or below the upper and lower age limits.

**Results:** Growth curves for percentiles were constructed for both sexes and higher values were noticeably found to set as growth-standard compared to WHO-standards.

**Conclusion:** Our analysis shows that the WHO 2007 standard references are not suitable for Eastern-Spanish children. The standards shown in this study are much more realistic and current, and we believe that their use will help healthcare professionals more effectively combat the current epidemic of overweight and obesity.

## Introduction

According to the latest report on obesity and overweight from the World Health Organization (WHO), more than 340 million children and adolescents between the ages of 5 and 19 were overweight and obese worldwide. Years ago, it was considered a nutritional disorder typical of industrialized countries (1) but currently represents one of the major social and health problems for both developed and developing countries (2, 3).

The WHO uses child growth standards that analyze specific percentiles and standard deviations according to sex and age to define overweight and what constitutes obesity in childhood and adolescence. Growth patterns represent the distribution of an anthropometric measure in a population, reflecting its nutritional status. They constitute a useful and appropriate instrument for carrying out longitudinal follow-up of infants and to detect those who present a nutritional risk (4–6).

Body Mass Index (BMI) by age and sex is currently the most widely used metric for estimating overweight and obesity due to its high sensitivity (7–9). In Spain, the BMI growth curves of the Orbegozo Foundation (10, 11) and those of the WHO 2007 (12) are mainly used.

Anthropometric measurements yield significant information about a child's growth and nutritional status, although it is often not easy to obtain accurate and useful measurements because we may find a child has physical limitations or an inappropriate procedure has been followed. Knowledge of appropriate anthropometric metrics and alternatives is essential for assessing children's growth and focusing on those who may be physically disabled or seriously ill (13).

The use of different growth tables to assess the population's nutritional status has given rise to a series of limitations arising from the lack of consensus and uniform methodological criteria. This leads to a disparity of results that prevent an accurate and reliable diagnosis of whether a child is overweight or obese (14, 15). Taking correct anthropometric measurements and properly interpreting them play a fundamental role in identifying children who are overweight or obese.

Worldwide, there is a great consensus on the use of the WHO child growth standards, but despite the fact that international standards unquestionably allow comparison between countries, they may not be applicable to all populations mainly due to epidemiological changes, genetic differences, and different growth standards (16). The fact that the WHO study was conducted with a population that was breastfed and that it only included one European country, suggests the need for countries to use their own internal population standards (11).

Another significant controversial point resides in the definition of the cut-off points for defining what constitutes overweight or obesity. Many studies define obesity with the P95 and overweight with the P85 for epidemiological studies and clinical screening (8, 12), although the most widely used criterion in international publications is that established by Cole et al., together with the IOTF, which establishes the definition of overweight or obese at the age of 18 at a BMI ≥ 25 and a BMI ≥ 30 Kg/m^2^, respectively, projecting percentiles calculated to other age groups, as suggested by the International Obesity Task Force (IOTF) (17).

Based on all of the above and considering that national growth standards, if available, may be more appropriate for evaluating growth deviations (18), the purpose of this study was to develop standard growth references for weight, height, and BMI for Spanish-eastern children and adolescents from 6 to 16 years of age.

## Materials and Methods

### Sample Description

This is a cross-sectional study. Study participants were selected in two stages. Firstly, educational centers and sports centers were randomly selected for recruitment of children who were then invited to participate. After the initial interview, members of 17 elementary and secondary education centers and 4 sports centers, a football club, a basketball club, a handball club, and a swimming club agreed to participate. After teachers, parents or legal guardians were duly informed, a total of 1,183 children (630 boys and 553 girls) between 6 and 16 years-of-age participated in the study, which was approved by the Research Ethics Committee of the General Directorate of Public Health and Higher Center for Research in Public Health (CEI DGSP-CSISP) on September 28, 2018, with number 20180928/03. Written informed consent was obtained from all participants. Data was collected between October 2018 and May 2020.

### Metrics

Anthropometric data were collected with light clothing on and without shoes. A bioelectric impedance scale (Omron® model HBF-511B-E with 150.0 kg capacity) was used to calculate weight, lean mass, and fat mass. Precision for weight was 0.1 kg and precision was 3.5% for fat mass and lean mass. A SECA brand wall height measurement rod with a range of 750–2,000 mm and with precision to within 1 millimeter was used to determine height. This determination was made with the children barefoot, in an upright position, feet together and with their backs to the height rod, keeping the head in the Frankfort horizontal plane. An anatomical tape measure with precision to within one millimeter was used to obtain body circumferences and perimeters. Measurement of the contours was performed with the subject standing upright, feet together, with the abdomen relaxed and with normal breathing.

Skin folds were measured following the procedures established by the International Society of the Advancement of Kinanthropometry (ISAK) with a basic Slim Guide caliper. The tricipital, bicipital, abdominal, suprailiac and subscapular folds were analyzed.

### Construction of Growth Curves

Children whose weight or BMI was above or below 3 standard deviations (SD) were excluded to avoid the influence of unhealthy weights. This followed the same approach used in the construction of the cross-sectional curves published by the WHO [12]. Children of immigrants (19) and those who suffered some pathology that influenced growth were also excluded. As a result, 42 observations for boys (3.5%) and 39 observations for girls (3.5%) were excluded. The final sample used to fit the growth curves was made up of 1,102 observations (588 boys and 514 girls).

Data were entered and stored in an MS Excel file and then transferred to SPSS v.23 software (SPSS Inc., Chicago, IL, USA) for descriptive statistical analysis. Anthropometric data for weight, height, and BMI are presented using mean and SD. The Kolmogorov-Smirnov goodness-of-fit test was used to analyze the normality of the populations and the unpaired Student *T*-test to analyze differences between both sexes. The z-test of comparison of proportions was used to analyze the differences between the values obtained using the own reference and the WHO 2007 reference. The two sample Anderson-Darling goodness-of-fit analysis was used to compare the curves.

The BCPE method (20) with smoothing of curves using cubic splines was used to construct the curves. The best fit was obtained with the following parameters: For height-for-age: BCPE [df (μ) = 5.5, df (σ) = 2.15, df (ν) = 2, df (τ) = 2] for children and BCPE [df (μ) = 6, df (σ) = 4, df (ν) = 2, df (τ) = 5] for girls. For weight for age: BCPE [df (μ) = 5.6, df (σ) = 4.6, df (ν) = 2.4, df (τ) = 2)] for boys and BCPE [df (μ) = 5.5, df (σ) = 4.7, df (ν) = 4.5, df (τ) = 2] for girls. For BMI by age: BCPE [df (μ) = 2, df (σ) = 4, df (ν) = 2, df (τ) = 4] for boys and BCPE [df (μ) = 2, df (σ) = 4, df (ν) = 5, df (τ = 2.5)] for girls. Df (μ), df (σ), df (ν) and df (τ) represent the degrees of freedom of the cubic splines that adjust the median, the coefficient of variation, the Box-Cox transformation power, and kurtosis, respectively. The transformation powers were λ = 1.5 for the weight of boys and girls and for the height of boys, λ = 0.08 for the BMI of boys and λ = 1 for the BMI of girls. Finally, a value of λ = 5.84e-5 was applied for the height-for-age of the girls. Once the models were adjusted, the 3, 5, 10, 25, 50, 75, 85, 90, 95, and 97 percentiles were estimated for each of the curves. The analysis was done using R version 4.0.3 (21), with the modeling in gamlss version 5.2-0 (22).

## Results

Table 1 summarizes the characteristics of the sample. The fact that children and youth sports clubs were included in the selection of centers in the first step of the sampling was due to the ease of accessing children of the indicated ages, as well as public and private schools. The selected clubs collaborate in the extracurricular activities of the children. They are not elite sports clubs that can contribute significant bias. Many of the children were even overweight or obese and attend clubs to lose weight. Table 2 shows an analysis by gender of the mean values of weight, height and BMI of the children differentiated by attendance or not at sports clubs. There are no statistically significant differences between them.

**Table 1:** Descriptive statistics for demographic information.

**Gender**	**Age (years)**	**Male**			**Female**			***P*-value^a^**
		***n***	**Mean (SD)^b^**	**Min–Max**	***n***	**Mean (SD)^b^**	**Min–Max**	
Height (cm)	6	54	121.3 (4.5)	112.5–130.2	32	118.6 (4.0)	109.5–126.2	**0.007**
	7	30	128.4 (4.4)	119–136	33	126.1 (4.6)	117.9–134	**0.049**
	8	74	131.7 (4.6)	122–142	54	130.7 (5.0)	119–139.2	0.236
	9	58	138.8 (5.4)	125.4–150	41	136.3 (6.0)	125–149.9	**0.031**
	10	92	143.9 (5.2)	131.1–155	79	144.1 (7.3)	129.5–155	0.787
	11	73	149.0 (6.4)	135.3–162.6	63	151.2 (5.4)	137–163	**0.034**
	12	63	154.8 (6.9)	140.6–168	80	154.7 (7.6)	132.7–169	0.954
	13	38	161.0 (7.7)	135.9–172	27	158.2 (4.1)	150–165	0.097
	14	67	166.4 (7.5)	151–183	48	162.0 (5.2)	153.5–178	**0.001**
	15	22	172.9 (7.8)	157–190	30	161.8 (6.1)	153–176.9	**0.000**
	16	17	173.1 (5.6)	160–180.5	27	163.2 (6.4)	149.5–179.8	**0.000**
Weight (kg)	6	54	23.4 (3.0)	18.2–30.3	32	22.5 (3.2)	17.3–32.3	0.171
	7	30	28.1 (3.9)	20.9–35.6	33	27.5 (4.2)	21–36.3	0.548
	8	74	30.2 (5.1)	21.9–43.5	54	30.0 (5.1)	22.5–41.8	0.868
	9	58	35.6 (5.4)	27.3–49.1	41	33.2 (5.8)	22.1–44	**0.036**
	10	92	40.3 (6.3)	26–55	79	38.6 (8.1)	21.9–54.4	0.122
	11	73	42.1 (9.8)	25.3–68.1	63	43.3 (8.0)	27.7–60.4	0.445
	12	63	46.5 (9.0)	30.4–70.1	80	48.3 (9.1)	25.6–69.4	0.247
	13	38	52.9 (7.8)	35.4–68.8	27	51.0 (10.2)	36–73.8	0.395
	14	67	56.8 (7.4)	41.3–79.4	48	53.0 (6.3)	41.1–67.6	**0.005**
	15	22	61.2 (11.8)	41.5–84.3	30	58.9 (10.5)	45.7–89.5	0.459
	16	17	68.2 (14.2)	51.8–110	27	58.8 (8.1)	41.6–79	**0.008**
BMI (Kg/m^2^)	6	54	15.9 (1.6)	12.5–19.5	32	15.9 (1.7)	12.6–20.3	0.895
	7	30	17.0 (1.8)	13.6–20.8	33	16.3 (2.1)	13.7–21.2	0.648
	8	74	17.1 (2.2)	13.6–22.1	54	17.5 (2.2)	14.1–23.1	0.362
	9	58	18.2 (2.0)	14.6–23.5	41	17.8 (2.2)	13.6–21.4	0.343
	10	92	19.4 (2.8)	14.3–25.4	79	18.5 (3.0)	12.8–24.9	**0.034**
	11	73	19.1 (2.9)	15–26	63	18.8 (2.7)	14.3–24.8	0.586
	12	63	19.3 (2.9)	15–28.4	80	20.1 (3.0)	14.5–27.6	0.124
	13	38	20.5 (3.2)	15.7–32.5	27	20.3 (3.5)	15.0–27.4	0.840
	14	67	20.5 (2.3)	16.8–28.8	48	20.2 (2.3)	16.1–26.6	0.522
	15	22	20.4 (2.9)	16.5–20.9	30	22.4 (3.2)	17.3–29.4	**0.022**
	16	17	21.9 (2.7)	18.9–27.8	27	22.1 (2.9)	17.9–30.0	0.874

a *Unpaired T-test*.

b *SD, Standard Deviation*.

*Values in bold indicates statistically significant differences*.

**Table 2:** Differences by gender of the mean values of weight, height and BMI of children differentiated by attendance or not at sports clubs.

	**Sport center**		*p*-value^*^
	**No**	**Yes**	
**Male**			
Weight [mean (SD)]	41.2 (13.35)	42.82 (14.67)	0.128
Height	147.68 (15.94)	149.51 (19.37)	0.243
BMI	18.28 (2,58)	18.31 (2.44)	0.877
**Female**			
Weight [mean (SD)]	42.15 (12.1)	41.02 (14.46)	0.452
Height	147.77 (14.29)	144.89 (17.13)	0.109
BMI	18.79 (2.75)	18.7 (3.09)	0.776

* *Unpaired T-test*.

**Figure 1:**
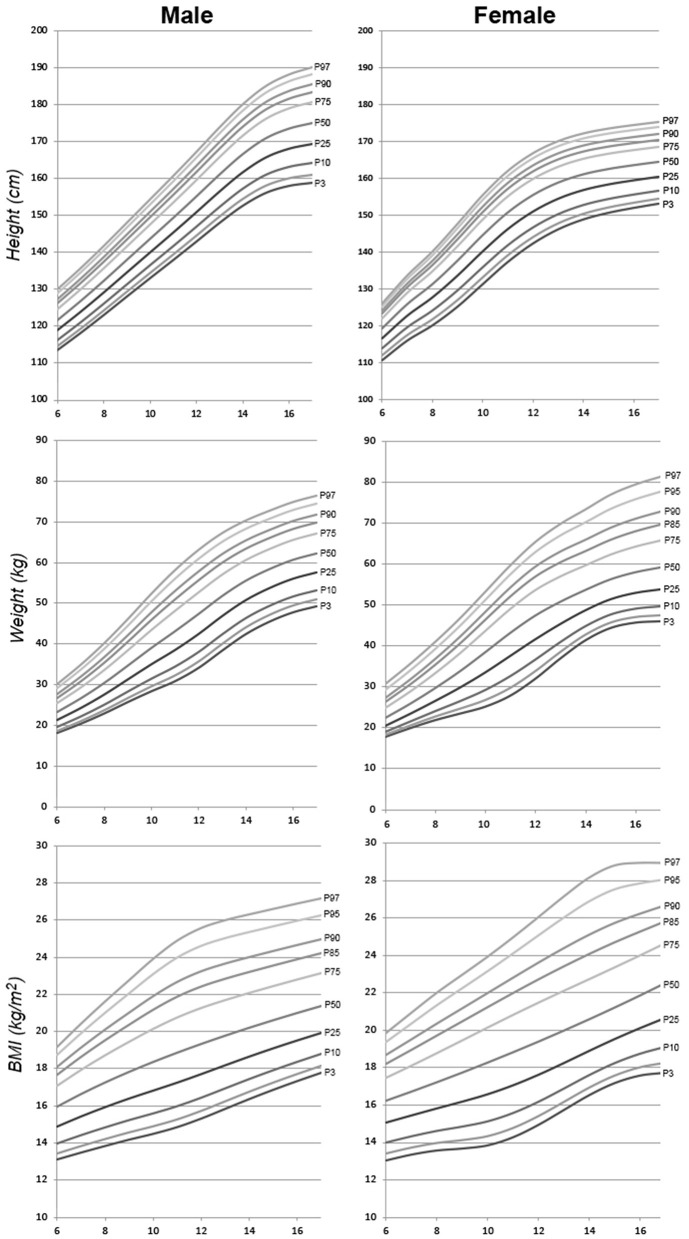
Spanish-eastern children height, weight, and BMI reference charts.

**Figure 2:**
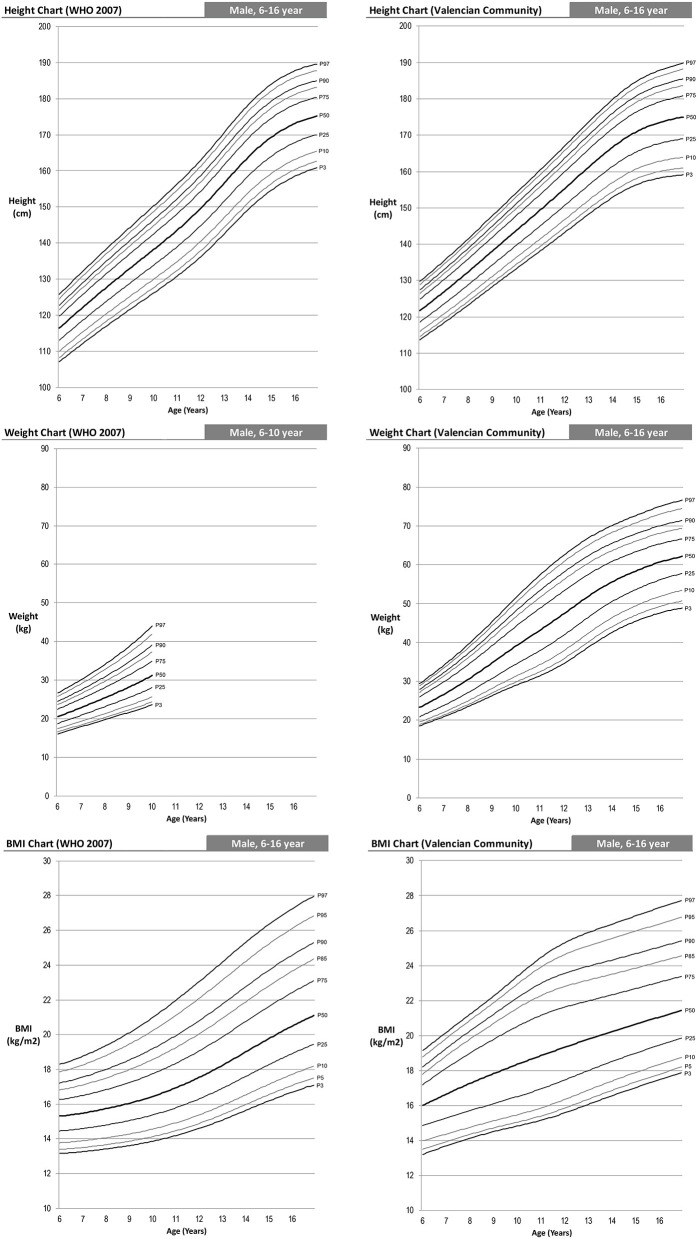
WHO 2007 and Spanish-eastern children male reference charts.

**Figure 3:**
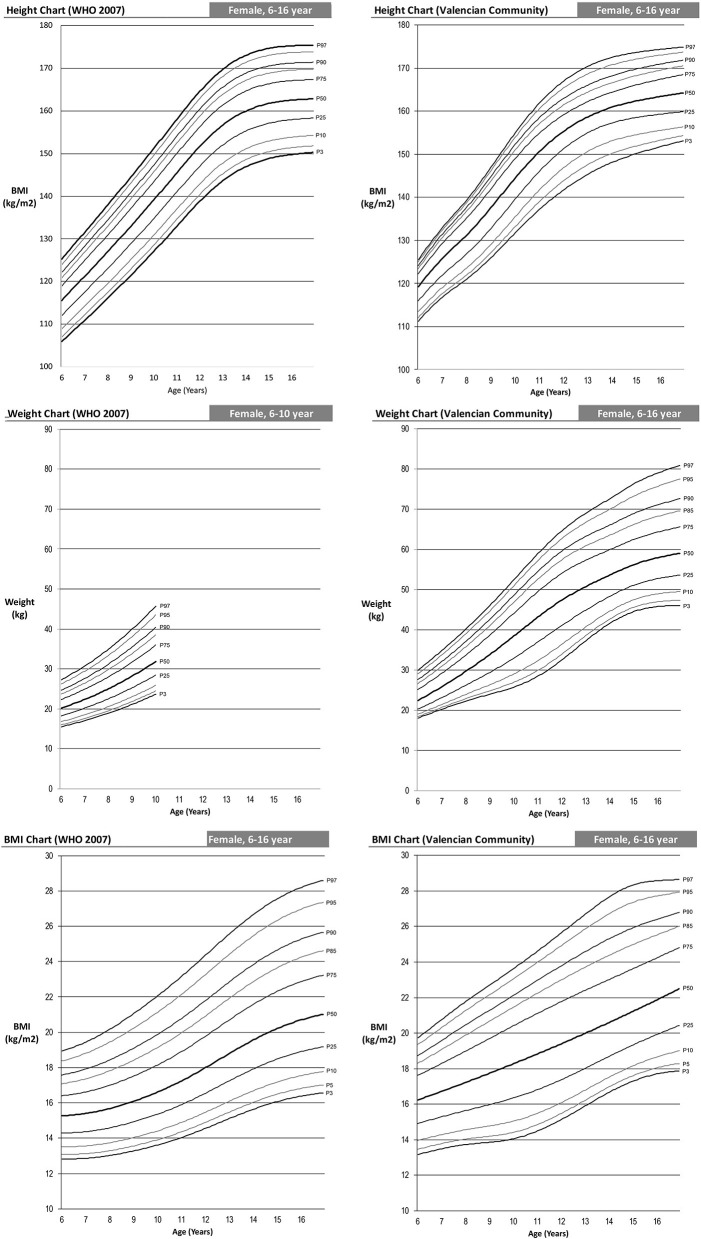
WHO 2007 and Spanish-eastern children female reference charts.

**Figure 4:**
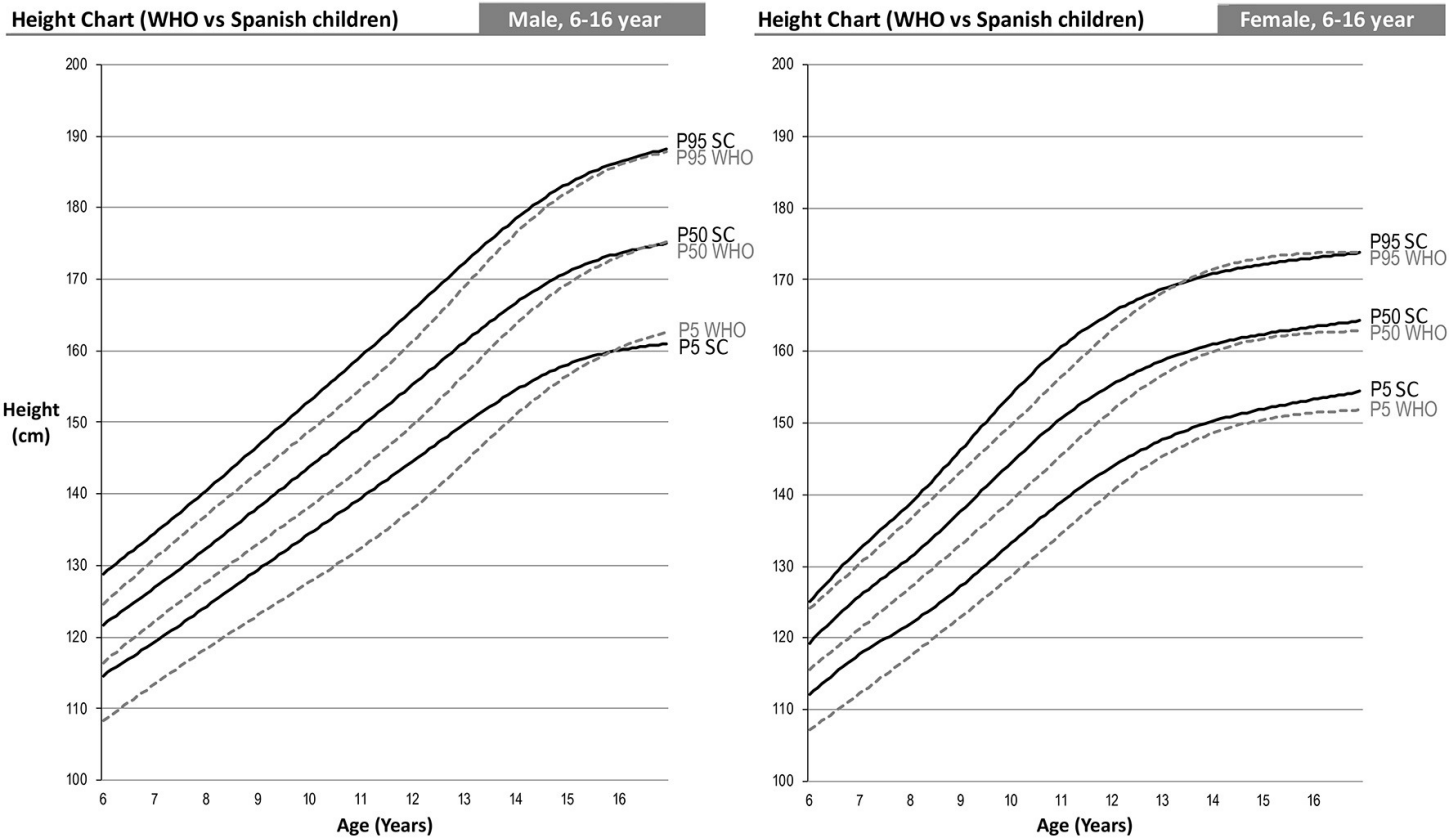
Comparison of the 2007 WHO height percentile and children from eastern Spain. Two-sample Anderson Darling test for goodness of fit of the curves: male 5th percentile (AD: 3.84; *p* = 0.010); Male 50th percentile (AD: 3.03; *p* = 0.026); male 95th percentile (AD: 3.24; *p* = 0.020); female 5th percentile (AD: 5.39; *p* = 0.002); female 50th percentile (AD: 3.60; *p* = 0.013); female 95th percentile (AD: 3.08; *p* = 0.025).

**Figure 5:**
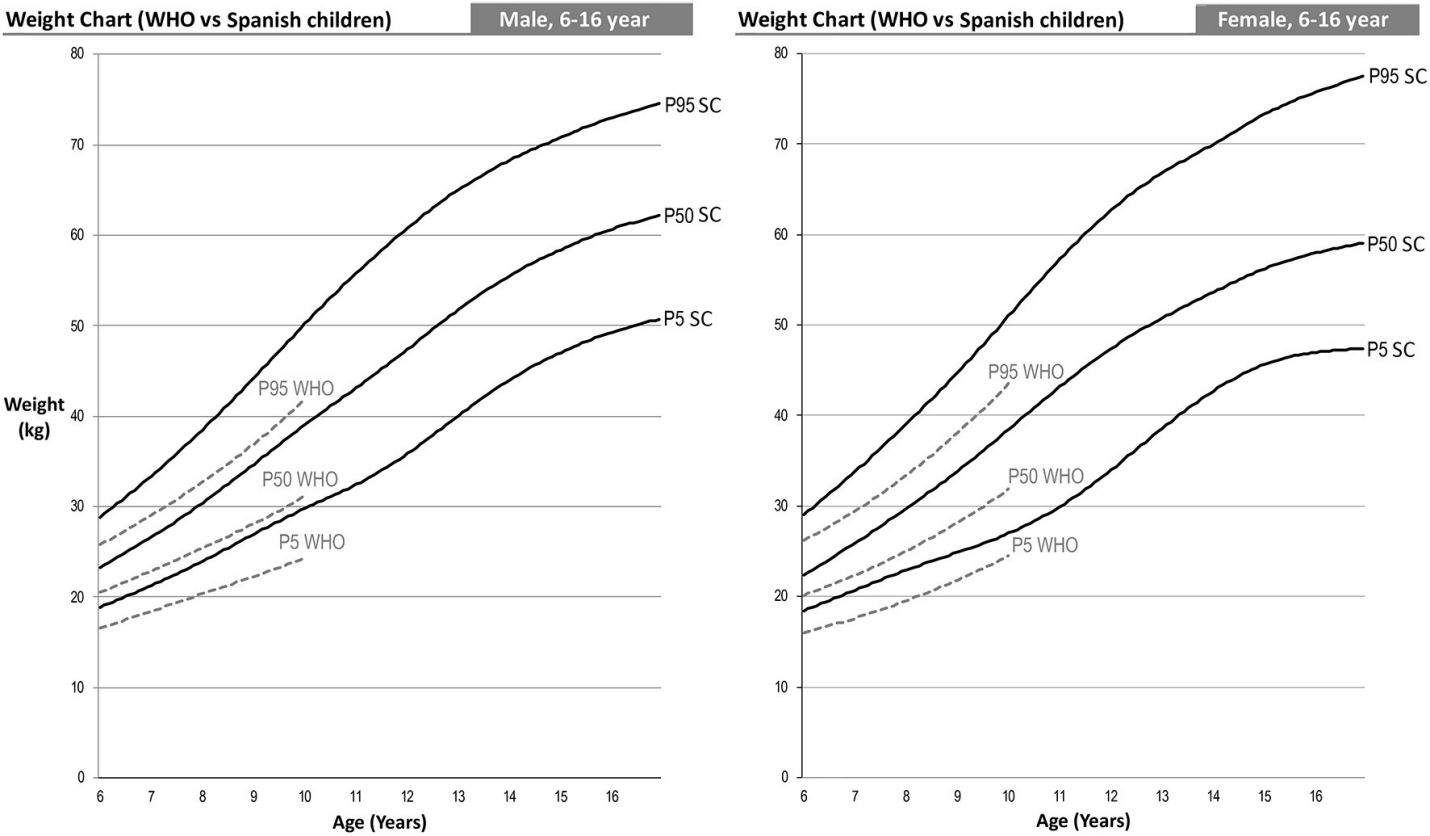
WHO 2007 and Spanish-eastern children weight percentile comparison. Two-sample Anderson Darling test for goodness of fit of the curves: male 5th percentile (AD: 14.33; *p* = 0.000); Male 50th percentile (AD: 13.88; *p* = 0.000); male 95th percentile (AD: 10.00; *p* = 0.000); female 5th percentile (AD: 13.03; *p* = 0.000); female 50th percentile (AD: 11.22; *p* = 0.000); female 95th percentile (AD: 8.71; *p* = 0.000).

**Figure 6:**
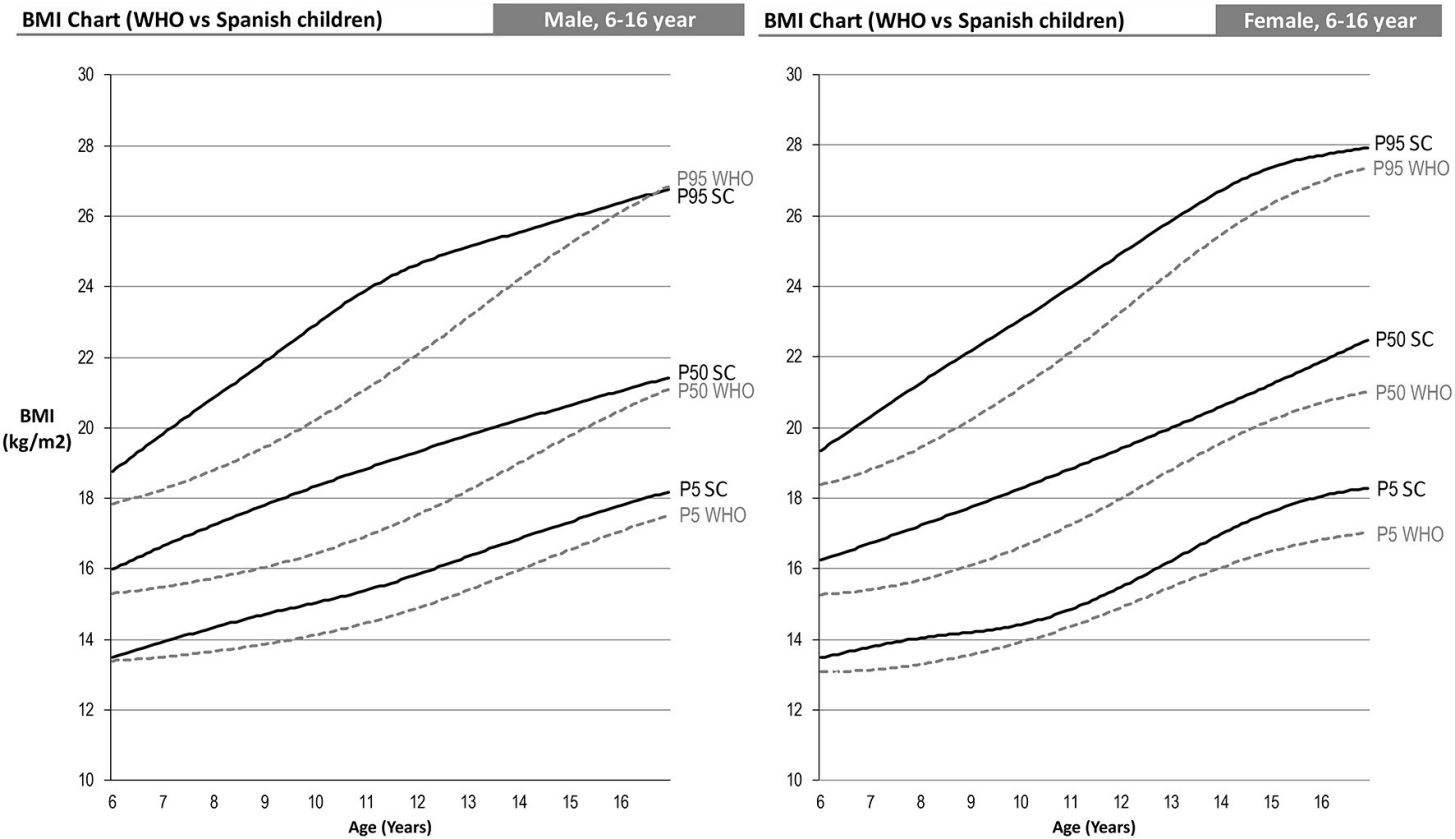
WHO 2007 and Spanish-eastern children BMI percentile comparison. Two-sample Anderson Darling test for goodness of fit of the curves: male 5th percentile (AD: 11.93; *p* = 0.000); Male 50th percentile (AD: 19.94; *p* = 0.000); male 95th percentile (AD: 13.50; *p* = 0.000); female 5th percentile (AD: 7.03; *p* = 0.000); female 50th percentile (AD: 17.27; *p* = 0.000); female 95th percentile (AD: 10.63; *p* = 0.000).

The following are differentiated by sex the graphs of height for age, weight for age and BMI for age of the Spanish-eastern children (Figure 1). The data is provided as Supplementary Tables 2–7. Subsequently, the graphs obtained are compared with WHO growth curves published in 2007 (12) (Figures 2–**6**). The final graphs appear as Supplementary Material and will be available for download at SONEV Research Group site, as well as the CV2021 data file prepared for use with the LMSGrowth software (a Microsoft Excel add-in) developed by Tim Cole, from University College London), downloadable free of charge from https://www.healthforallchildren.com.

### Reference Charts

#### Height Comparison

The shape of our growth charts is similar to those of the WHO (Figure 4). Nevertheless, one can observe that as from the age of 6, the Spanish-eastern children are taller. In the case of boys, at 6 years of age they have values of 6.7, 4.95 and 4.3 cm higher in the 5th, 50th, and 95th percentiles, respectively, and girls, 5, 3.7, and 1 cm higher in the same percentiles. This difference remains approximately the same up to 13 years of age in boys and 12 years of age in girls, ages from which it gradually begins to decrease. At 16 years of age, the percentiles of boys in both curves are practically the same, but girls continue to be taller in the east of Spain.

### Weight Comparison

Comparison of weight percentiles can only be performed up to age 10 since the WHO age-based weight curves are only performed up to that age. In this case, we can see an effect similar to that of height, i.e., Spanish-eastern children have a greater weight from the age of 6 (Figure 5). This difference increases up to 10 years of age, the age at which we find higher weights in the Spanish-eastern children of 5.4, 7.9, and 8.3 kg in the 5th, 50th, and 95th percentiles of boys and 2.5; 6.6 and 7.5 kg higher in the same percentiles of girls.

### BMI Comparison

The combined effect analyzed in the weight and height curves (Figures 4, 5) in turn causes the differences in the BMI curves of the WHO and the Spanish-eastern children that we observe in Figure 6. Both boys and girls in the east of Spain already have a higher BMI at the age of 6 with respect to the levels of the WHO. This difference reaches its highest level around the age of 9 in girls and 11 years of age in boys, ages in which we find a higher BMI in the 5th, 50th, and 95th percentiles of 1, 2, and 2 Kg/m^2^ in the boys and 0.7, 1.6, and 2 Kg/m^2^ in girls. The difference in BMI is gradually reduced in boys until it becomes equal in the upper percentiles in which the heights are practically the same as has been analyzed in Figure 4. However, the difference in BMI remains at values close to 1 Kg/m^2^ higher in girls.

**Figure 7:**
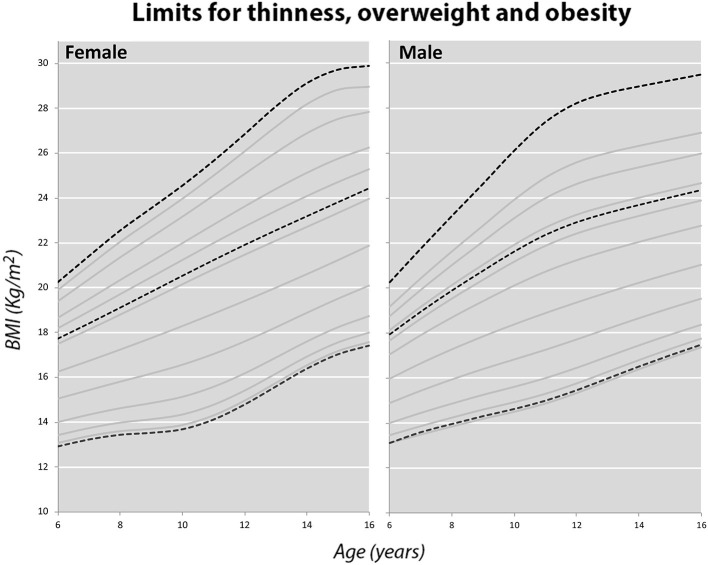
International cut-off points for body mass index by sex for thinness, overweight and obesity.

Limits have been established for thinness, overweight and obesity in both boys and girls. Therefore, three curves have been modeled in each BMI graph corresponding to the curves that will cross through 18.5, 25, and 30 kg/m^2^ (17) respectively at age 18, as can be seen in Figure 7. The limits of overweight and obesity correspond, respectively, to an 88th percentile (z score +1.19) and a 99th percentile (z score +2.46) in boys. The limits for girls are the 79th percentile (z score +0.81) for overweight and the 98th percentile (z score +2.05). In the case of thinness, the limits correspond to a 3.5 percentile (−1.81 z-score) for boys and a 2.5 percentile (−1.96 z-score) for girls. Applying these values to our sample, we obtain the results that can be seen in Table 3. In all cases, the percentages obtained by applying the local reference are significantly lower than by applying the WHO 2007 references.

**Table 3:** Comparison of the proportions of thinness, overweight and obesity obtained with the local references and with the 2007 WHO reference.

**Thinness**			**Overweight**			**Obesity**		
**Local reference**	**WHO reference**	*p*-value^*^	**Local reference**	**WHO reference**	*p*-value^*^	**Local reference**	**WHO reference**	*p*-value^*^
**Male**								
2.9%	5.6%	0.010	12.4%	27.5%	0.000	2.4%	5.4%	0.000
**Female**								
1.75%	3.5%	0,039	18.9%	36.4%	0.000	2.7%	5.4%	0.000

* *Difference of proportions z test*.

## Discussion

The 2007 WHO curves (12) are currently used in the east of Spain for Child Health Service Cards. Therefore, to make the comparison of the internal values obtained as realistic as possible, the same construction method has been used for the internal curves, modeling age as a continuous variable and simultaneously adjusting the curves, smoothing them using cubic splines and further smoothing the edge effects by means of data extending above or below the upper and lower age limits (12).

There are obvious differences in growth reference values between boys and girls (Table 1). At age 6, boys are, on average, 2.70 cm taller and 900 g heavier than girls. At age 16, males are, on average, almost 10 cm taller and 10 kg heavier than females. Height and weight values for boys are higher than for girls at all ages except for 10–12 years due to the onset of puberty in girls. We found significant differences in BMI values according to age between both sexes at some ages (Table 1), but these differences were small and of little clinical relevance, which coincides with other studies (23, 24). As indicated in them, the differences observed between the sexes in weight according to age can be mostly associated with proportional differences in height according to age.

Our analysis shows that the use in our population of the WHO standard growth references published in 2007 and based on historical statistical data from the National Center for Health Statistics (NCHS) from 1977 can lead to misdiagnoses. Our children are taller and weigh more, so the differences in BMI are notable; values differ by up to 2 Kg/m^2^. This can lead to overestimating the prevalence of obesity. As an example, an 11-year-old boy with a BMI of 22 would be in a 90th percentile according to our references and yet he would be in a value higher than the 97th percentile according to the WHO reference. These differences, as we have seen in the results, can be explained above all by the increase in height of the Spanish-eastern children. The large differences in the height of children from different European countries have been described previously (25), which causes differences in growth standards, especially in Spain, where mean height continues to grow secularly (26).

A study carried out in Spain between 2000 and 2004 (27) found remarkably similar BMI growth standards to those found in this study, with a secular growth acceleration of about 14 cm during the last century, accompanied by a corresponding acceleration in weight, which is consistent with our findings. Thus, a recent survey will better reflect the current height of the population, even if it runs the risk of including a growing number of overweight and obese children.

Another cross-sectional study conducted in Italy (28) also found the same BMI pattern in boys and girls. As in our community, Italian children are taller than the WHO reference children, equaling the height at the end of growth. As for the BMI, its standard, similar to ours, was quite different from that of the reference. Reasons argued for the similarity of standards may be that Spanish and Italian children share the Mediterranean diet and lifestyles.

Other studies have shown similar effects in the opposite direction (29, 30), indicating that the use of the WHO 2007 growth charts may lead to overestimating the prevalence of being underweight and lead pediatricians to incorrectly identify a child as being stunted or underweight. This seems to indicate that the use of the WHO 2007 standards may not be useful in populations with genetic and environmental differences from the WHO reference population (30–32). For these reasons, the European Nutrition Commission (25) also recommended that each European nation analyze the usefulness of these references in their populations. As already indicated, the cut-off points calculated to detect overweight and obesity have followed the study by Cole et al. (17), since they are linked to the widely accepted cut-off points for adults of a body mass index of 25 and 30 kg/m^2^, which avoids arbitrary choices and is recommended for use in international comparisons of the prevalence of overweight and obesity. The values found in our sample are similar to those found in other studies (23, 24) As we have seen previously, if we had applied the WHO 2007 references (12), the rate of overweight and obesity doubles with respect to our references, which again affects the idea that the use of growth standards developed in another population can lead to misdiagnoses, in this case an overestimation of the prevalence of obesity. In our opinion, the differences found between our analysis and the standard currently used in our community are clinically relevant, so it is important to have up-to-date growth charts, especially in the extreme percentiles (33).

A limitation of the present work, as in most population studies, could be related to the measurements themselves. However, we hope to have minimized it as much as possible by having carried out all the measurements with the same instruments and by the same researchers from the SONEV Research Group (Overweight, Obesity, and Lifestyles). Furthermore, we are aware that, in the case of cross-sectional data, the curves do not provide information on the crossing of percentiles over time, which is considered a weakness of growth charts based on cross-sectional data (17). Another limitation of the present study is the size of the sample, although we consider that it is sufficient to show that the WHO growth standard, carried out with data from more than 40 years of selected children, is not currently applicable to children from eastern Spain. On the other hand, we consider that, since there are no ethnic or sociodemographic differences between the different areas of our country, the results obtained can be applied to all children in the country. It is necessary to supplement the findings with a longitudinal study in order to establish growth standards for the entire country, as well as periodically update the references shown here.

## Data Availability Statement

The original contributions presented in the study are included in the article/Supplementary Material, further inquiries can be directed to the corresponding author/s.

## Ethics Statement

The studies involving human participants were reviewed and approved by Research Ethics Committee of the General Directorate of Public Health and Higher Center for Research in Public Health (CEI DGSP-CSISP) with number 20180928/03. Written informed consent to participate in this study was provided by the participants' legal guardian/next of kin.

## Author Contributions

MP-B, MM-L, and LA-D: idea and design of the study. MP-B, JP-M, LA-D, ML-G, and MM-L: participant enrolment and data collection. MP-B and MM-L: organized the database and performed the statistical analysis. MP-B, JP-M, and MM-L: wrote the first draft of the manuscript. LA-D and ML-G: wrote sections of the manuscript. All authors contributed to manuscript revision, read, and approved the submitted version.

## Conflict of Interest

The authors declare that the research was conducted in the absence of any commercial or financial relationships that could be construed as a potential conflict of interest.

## Publisher's Note

All claims expressed in this article are solely those of the authors and do not necessarily represent those of their affiliated organizations, or those of the publisher, the editors and the reviewers. Any product that may be evaluated in this article, or claim that may be made by its manufacturer, is not guaranteed or endorsed by the publisher.
