# Supplementary material for: Are the Growth Standards of the World Health Organization Valid for Spanish Children? The SONEV Study
Source: Front Pediatr. 2021 Aug 16;9:700748. doi: 10.3389/fped.2021.700748 (PMC8418137; doi:10.3389/fped.2021.700748)
Supplement: Supplementary file 1 [file Data_Sheet_1.docx]

Supplementary Material

**Supplementary Table 1.** **Spanish-eastern children L, M and S reference parameters for height, weight and BMI for age.**

| Year | Height | | | | | |
| --- | --- | --- | --- | --- | --- | --- |
|  | Boys | | | Girls | | |
|  | L | M | S | L | M | S |
| 6 | 0,9258694 | 121,7390 | 0,03587409 | 5,178730 | 119,3084 | 0,03319992 |
| 7 | 0,9914108 | 126,9202 | 0,03666315 | 4,663735 | 125,8621 | 0,03626021 |
| 8 | 1,0618172 | 132,3997 | 0,03753094 | 4,217562 | 131,2578 | 0,03954829 |
| 9 | 1,1367732 | 138,1015 | 0,03847594 | 3,823868 | 137,6287 | 0,04248421 |
| 10 | 1,2160175 | 143,7853 | 0,03949017 | 3,471529 | 144,5025 | 0,04373765 |
| 11 | 1,2993294 | 149,4541 | 0,04055616 | 3,152606 | 150,6477 | 0,04312744 |
| 12 | 1,3865192 | 155,2717 | 0,04164922 | 2,861296 | 155,3536 | 0,04166570 |
| 13 | 1,4774215 | 161,2035 | 0,04275767 | 2,593210 | 158,7173 | 0,03998597 |
| 14 | 1,5718903 | 166,7095 | 0,04388683 | 2,344949 | 160,9580 | 0,03856624 |
| 15 | 1,6697958 | 171,0188 | 0,04504551 | 2,113810 | 162,4001 | 0,03751361 |
| 16 | 1,7710212 | 173,6222 | 0,04624936 | 1,897596 | 163,4370 | 0,03671296 |
| 17 | 1,8754611 | 175,1194 | 0,04751832 | 1,694498 | 164,3413 | 0,03603738 |
| Year | Weight | | | | | |
|  | Boys | | | Girls | | |
|  | L | M | S | L | M | S |
| 6 | -0,1516864 | 23,26660 | 0,1334140 | -1,1943771 | 22,42863 | 0,1382796 |
| 7 | -0,0557502 | 26,62264 | 0,1415404 | -0,7239893 | 25,90370 | 0,1506136 |
| 8 | 0,0483724 | 30,42711 | 0,1483584 | -0,1851625 | 29,72405 | 0,1652688 |
| 9 | 0,1572977 | 34,69398 | 0,1542678 | 0,3476992 | 33,88086 | 0,1811605 |
| 10 | 0,2649550 | 39,04646 | 0,1617620 | 0,7167335 | 38,47460 | 0,1933157 |
| 11 | 0,3621143 | 43,13919 | 0,1671560 | 0,8070532 | 43,19418 | 0,1953417 |
| 12 | 0,4396537 | 47,39808 | 0,1619585 | 0,5688374 | 47,41890 | 0,1865789 |
| 13 | 0,4966533 | 51,80834 | 0,1477431 | 0,0021615 | 50,78639 | 0,1683217 |
| 14 | 0,5369029 | 55,56683 | 0,1332213 | -0,6391511 | 53,66710 | 0,1498074 |
| 15 | 0,5650722 | 58,41441 | 0,1237530 | -1,0210124 | 56,24477 | 0,1419369 |
| 16 | 0,5857875 | 60,70064 | 0,1184295 | -1,0181429 | 57,98820 | 0,1426638 |
| 17 | 0,6029417 | 62,26622 | 0,1157770 | -0,8410811 | 59,15725 | 0,1478953 |
| Year | BMI | | | | | |
|  | Boys | | | Girls | | |
|  | L | M | S | L | M | S |
| 6 | 0,2970788 | 15,95976 | 0,1008363 | 0,35240363 | 16,23753 | 0,1115524 |
| 7 | 0,0745806 | 16,64319 | 0,1103408 | -0,03771495 | 16,72309 | 0,1196519 |
| 8 | -0,1203530 | 17,26572 | 0,1185025 | -0,14577650 | 17,22492 | 0,1284939 |
| 9 | -0,2940077 | 17,83960 | 0,1255151 | 0,00846078 | 17,74334 | 0,1378611 |
| 10 | -0,4507188 | 18,37352 | 0,1323911 | 0,08936757 | 18,27875 | 0,1454912 |
| 11 | -0,5936068 | 18,87404 | 0,1359924 | -0,08181339 | 18,83161 | 0,1485644 |
| 12 | -0,7249942 | 19,34635 | 0,1345058 | -0,44399075 | 19,40239 | 0,1471568 |
| 13 | -0,8466574 | 19,79440 | 0,1295281 | -0,90796112 | 19,99157 | 0,1423360 |
| 14 | -0,9599866 | 20,22125 | 0,1237687 | -1,32582713 | 20,59971 | 0,1362665 |
| 15 | -1,0660904 | 20,62932 | 0,1185430 | -1,41564850 | 21,22735 | 0,1319896 |
| 16 | -1,1658675 | 21,02063 | 0,1139456 | -0,98279505 | 21,87503 | 0,1303790 |
| 17 | -1,2600571 | 21,39685 | 0,1097754 | -0,14931344 | 22,54341 | 0,1305683 |

**Supplementary Table 2.** **Centile reference values for male height**

| Months | 3th | 5th | 10th | 25th | 50th | 75th | 85th | 90th | 95th | 97th |
| --- | --- | --- | --- | --- | --- | --- | --- | --- | --- | --- |
| 72 | 113,7521 | 114,6322 | 116,0439 | 118,5913 | 121,739 | 124,8926 | 126,4502 | 127,4539 | 128,8766 | 129,7649 |
| 73 | 114,1345 | 115,0198 | 116,4396 | 119,0012 | 122,1655 | 125,3354 | 126,901 | 127,9099 | 129,34 | 130,2329 |
| 74 | 114,517 | 115,4075 | 116,8354 | 119,4112 | 122,5922 | 125,7783 | 127,352 | 128,3661 | 129,8037 | 130,7012 |
| 75 | 114,8998 | 115,7954 | 117,2316 | 119,8217 | 123,0193 | 126,2218 | 127,8036 | 128,8229 | 130,2679 | 131,1701 |
| 76 | 115,2829 | 116,1839 | 117,6282 | 120,2327 | 123,4472 | 126,666 | 128,256 | 129,2805 | 130,733 | 131,6399 |
| 77 | 115,6668 | 116,573 | 118,0256 | 120,6445 | 123,8759 | 127,1112 | 128,7093 | 129,7392 | 131,1991 | 132,1107 |
| 78 | 116,0514 | 116,9629 | 118,424 | 121,0573 | 124,3057 | 127,5576 | 129,1639 | 130,199 | 131,6666 | 132,5829 |
| 79 | 116,4371 | 117,354 | 118,8234 | 121,4714 | 124,7368 | 128,0054 | 129,6199 | 130,6604 | 132,1356 | 133,0566 |
| 80 | 116,824 | 117,7463 | 119,2242 | 121,8869 | 125,1695 | 128,4548 | 130,0777 | 131,1236 | 132,6064 | 133,5322 |
| 81 | 117,2123 | 118,14 | 119,6265 | 122,304 | 125,604 | 128,9062 | 130,5374 | 131,5887 | 133,0792 | 134,0099 |
| 82 | 117,6022 | 118,5354 | 120,0305 | 122,723 | 126,0405 | 129,3596 | 130,9993 | 132,0561 | 133,5543 | 134,4899 |
| 83 | 117,9939 | 118,9327 | 120,4365 | 123,144 | 126,4791 | 129,8155 | 131,4636 | 132,5259 | 134,032 | 134,9724 |
| 84 | 118,3876 | 119,3319 | 120,8445 | 123,5673 | 126,9202 | 130,2739 | 131,9306 | 132,9985 | 134,5124 | 135,4578 |
| 85 | 118,7834 | 119,7334 | 121,2549 | 123,9931 | 127,364 | 130,7351 | 132,4005 | 133,4739 | 134,9959 | 135,9462 |
| 86 | 119,1813 | 120,137 | 121,6675 | 124,4213 | 127,8103 | 131,199 | 132,8732 | 133,9523 | 135,4823 | 136,4377 |
| 87 | 119,5812 | 120,5427 | 122,0823 | 124,8518 | 128,2591 | 131,6657 | 133,3486 | 134,4334 | 135,9715 | 136,932 |
| 88 | 119,9831 | 120,9504 | 122,4992 | 125,2845 | 128,7104 | 132,1349 | 133,8267 | 134,9173 | 136,4636 | 137,4292 |
| 89 | 120,3868 | 121,3601 | 122,9181 | 125,7195 | 129,164 | 132,6066 | 134,3074 | 135,4038 | 136,9584 | 137,9292 |
| 90 | 120,7924 | 121,7717 | 123,339 | 126,1566 | 129,6199 | 133,0808 | 134,7907 | 135,8929 | 137,4558 | 138,4318 |
| 91 | 121,1998 | 122,1851 | 123,7619 | 126,5958 | 130,0781 | 133,5574 | 135,2764 | 136,3845 | 137,9558 | 138,9371 |
| 92 | 121,6088 | 122,6002 | 124,1866 | 127,0369 | 130,5384 | 134,0363 | 135,7645 | 136,8785 | 138,4583 | 139,4449 |
| 93 | 122,0195 | 123,0171 | 124,613 | 127,48 | 131,0008 | 134,5174 | 136,2548 | 137,3749 | 138,9632 | 139,9551 |
| 94 | 122,4318 | 123,4355 | 125,0412 | 127,925 | 131,4652 | 135,0006 | 136,7474 | 137,8736 | 139,4705 | 140,4678 |
| 95 | 122,8455 | 123,8555 | 125,471 | 128,3717 | 131,9315 | 135,486 | 137,2422 | 138,3744 | 139,98 | 140,9827 |
| 96 | 123,2607 | 124,277 | 125,9024 | 128,8201 | 132,3997 | 135,9733 | 137,739 | 138,8773 | 140,4916 | 141,4998 |
| 97 | 123,6772 | 124,6999 | 126,3353 | 129,2702 | 132,8696 | 136,4625 | 138,2377 | 139,3823 | 141,0054 | 142,019 |
| 98 | 124,0949 | 125,124 | 126,7694 | 129,7217 | 133,3412 | 136,9535 | 138,7383 | 139,8891 | 141,521 | 142,5402 |
| 99 | 124,5136 | 125,5492 | 127,2047 | 130,1744 | 133,8142 | 137,446 | 139,2405 | 140,3975 | 142,0383 | 143,0631 |
| 100 | 124,9331 | 125,9753 | 127,641 | 130,6283 | 134,2883 | 137,9398 | 139,744 | 140,9073 | 142,5571 | 143,5875 |
| 101 | 125,3533 | 126,402 | 128,078 | 131,083 | 134,7635 | 138,4347 | 140,2487 | 141,4184 | 143,0772 | 144,1132 |
| 102 | 125,7738 | 126,8292 | 128,5156 | 131,5384 | 135,2395 | 138,9306 | 140,7544 | 141,9305 | 143,5983 | 144,64 |
| 103 | 126,1947 | 127,2567 | 128,9536 | 131,9943 | 135,7161 | 139,4272 | 141,2609 | 142,4433 | 144,1203 | 145,1677 |
| 104 | 126,6156 | 127,6844 | 129,3917 | 132,4505 | 136,1931 | 139,9243 | 141,768 | 142,9568 | 144,643 | 145,6961 |
| 105 | 127,0364 | 128,1119 | 129,8299 | 132,9068 | 136,6703 | 140,4217 | 142,2754 | 143,4707 | 145,1661 | 146,225 |
| 106 | 127,4569 | 128,5393 | 130,2679 | 133,3631 | 137,1476 | 140,9193 | 142,783 | 143,9848 | 145,6894 | 146,7541 |
| 107 | 127,8769 | 128,9662 | 130,7055 | 133,8191 | 137,6247 | 141,4168 | 143,2906 | 144,4989 | 146,2128 | 147,2833 |
| 108 | 128,2963 | 129,3925 | 131,1425 | 134,2746 | 138,1015 | 141,914 | 143,7979 | 145,0128 | 146,736 | 147,8123 |
| 109 | 128,7149 | 129,818 | 131,5789 | 134,7295 | 138,5778 | 142,4108 | 144,3049 | 145,5263 | 147,2589 | 148,341 |
| 110 | 129,1326 | 130,2427 | 132,0145 | 135,1838 | 139,0535 | 142,9071 | 144,8114 | 146,0394 | 147,7814 | 148,8694 |
| 111 | 129,5496 | 130,6667 | 132,4495 | 135,6375 | 139,5287 | 143,4029 | 145,3175 | 146,5522 | 148,3035 | 149,3974 |
| 112 | 129,9657 | 131,0899 | 132,8837 | 136,0906 | 140,0034 | 143,8984 | 145,8232 | 147,0646 | 148,8254 | 149,9252 |
| 113 | 130,3811 | 131,5124 | 133,3173 | 136,5432 | 140,4776 | 144,3935 | 146,3286 | 147,5766 | 149,3469 | 150,4526 |
| 114 | 130,7957 | 131,9342 | 133,7503 | 136,9952 | 140,9514 | 144,8881 | 146,8336 | 148,0883 | 149,8681 | 150,9798 |
| 115 | 131,2096 | 132,3553 | 134,1826 | 137,4466 | 141,4247 | 145,3824 | 147,3383 | 148,5997 | 150,389 | 151,5067 |
| 116 | 131,6228 | 132,7758 | 134,6143 | 137,8976 | 141,8976 | 145,8764 | 147,8426 | 149,1108 | 150,9097 | 152,0334 |
| 117 | 132,0352 | 133,1955 | 135,0454 | 138,348 | 142,3701 | 146,37 | 148,3467 | 149,6216 | 151,4302 | 152,5599 |
| 118 | 132,447 | 133,6147 | 135,476 | 138,798 | 142,8422 | 146,8633 | 148,8505 | 150,1322 | 151,9504 | 153,0862 |
| 119 | 132,8582 | 134,0332 | 135,906 | 139,2475 | 143,3139 | 147,3563 | 149,3541 | 150,6426 | 152,4705 | 153,6123 |
| 120 | 133,2687 | 134,4511 | 136,3354 | 139,6966 | 143,7853 | 147,8491 | 149,8574 | 151,1527 | 152,9903 | 154,1382 |
| 121 | 133,6786 | 134,8685 | 136,7644 | 140,1453 | 144,2564 | 148,3416 | 150,3605 | 151,6627 | 153,51 | 154,664 |
| 122 | 134,088 | 135,2854 | 137,193 | 140,5937 | 144,7273 | 148,834 | 150,8635 | 152,1726 | 154,0297 | 155,1898 |
| 123 | 134,497 | 135,702 | 137,6213 | 141,0419 | 145,1981 | 149,3264 | 151,3665 | 152,6825 | 154,5495 | 155,7157 |
| 124 | 134,9058 | 136,1184 | 138,0495 | 141,4901 | 145,669 | 149,8189 | 151,8698 | 153,1927 | 155,0695 | 156,2419 |
| 125 | 135,3144 | 136,5347 | 138,4777 | 141,9384 | 146,1401 | 150,3117 | 152,3733 | 153,7032 | 155,5899 | 156,7685 |
| 126 | 135,7231 | 136,951 | 138,9059 | 142,3869 | 146,6115 | 150,8049 | 152,8773 | 154,2142 | 156,1108 | 157,2956 |
| 127 | 136,1318 | 137,3675 | 139,3345 | 142,8358 | 147,0833 | 151,2987 | 153,3819 | 154,7258 | 156,6323 | 157,8233 |
| 128 | 136,5408 | 137,7844 | 139,7634 | 143,2852 | 147,5558 | 151,7931 | 153,8872 | 155,2381 | 157,1547 | 158,3519 |
| 129 | 136,9502 | 138,2016 | 140,1928 | 143,7351 | 148,0289 | 152,2884 | 154,3934 | 155,7513 | 157,6779 | 158,8815 |
| 130 | 137,3601 | 138,6194 | 140,6228 | 144,1859 | 148,503 | 152,7846 | 154,9005 | 156,2656 | 158,2022 | 159,4121 |
| 131 | 137,7706 | 139,0379 | 141,0536 | 144,6374 | 148,978 | 153,2818 | 155,4088 | 156,7809 | 158,7277 | 159,9439 |
| 132 | 138,1818 | 139,4571 | 141,4852 | 145,09 | 149,4541 | 153,7803 | 155,9183 | 157,2976 | 159,2546 | 160,4771 |
| 133 | 138,5939 | 139,8773 | 141,9179 | 145,5437 | 149,9314 | 154,2801 | 156,4292 | 157,8157 | 159,7828 | 161,0117 |
| 134 | 139,0069 | 140,2984 | 142,3516 | 145,9985 | 150,41 | 154,7813 | 156,9415 | 158,3352 | 160,3126 | 161,5479 |
| 135 | 139,4208 | 140,7205 | 142,7863 | 146,4545 | 150,8899 | 155,2838 | 157,4553 | 158,8562 | 160,8439 | 162,0856 |
| 136 | 139,8356 | 141,1435 | 143,2221 | 146,9118 | 151,3711 | 155,7878 | 157,9705 | 159,3787 | 161,3767 | 162,6249 |
| 137 | 140,2514 | 141,5676 | 143,659 | 147,3702 | 151,8537 | 156,2932 | 158,4872 | 159,9027 | 161,9111 | 163,1658 |
| 138 | 140,6682 | 141,9928 | 144,097 | 147,8299 | 152,3376 | 156,8001 | 159,0054 | 160,4283 | 162,4471 | 163,7083 |
| 139 | 141,086 | 142,419 | 144,5362 | 148,2909 | 152,823 | 157,3086 | 159,5252 | 160,9554 | 162,9847 | 164,2525 |
| 140 | 141,5049 | 142,8463 | 144,9766 | 148,7532 | 153,3098 | 157,8185 | 160,0466 | 161,4842 | 163,524 | 164,7983 |
| 141 | 141,9248 | 143,2747 | 145,4181 | 149,2168 | 153,798 | 158,33 | 160,5696 | 162,0146 | 164,065 | 165,3459 |
| 142 | 142,3458 | 143,7043 | 145,8609 | 149,6818 | 154,2877 | 158,8431 | 161,0942 | 162,5467 | 164,6076 | 165,8952 |
| 143 | 142,7679 | 144,1351 | 146,305 | 150,1482 | 154,7789 | 159,3578 | 161,6205 | 163,0804 | 165,152 | 166,4462 |
| 144 | 143,1911 | 144,567 | 146,7503 | 150,616 | 155,2717 | 159,8741 | 162,1484 | 163,6159 | 165,6982 | 166,9991 |
| 145 | 143,6154 | 145,0001 | 147,1969 | 151,0851 | 155,766 | 160,392 | 162,678 | 164,1531 | 166,2461 | 167,5536 |
| 146 | 144,0406 | 145,4341 | 147,6445 | 151,5554 | 156,2614 | 160,9112 | 163,209 | 164,6916 | 166,7954 | 168,1096 |
| 147 | 144,4663 | 145,8686 | 148,0927 | 152,0264 | 156,7577 | 161,4314 | 163,7409 | 165,2311 | 167,3457 | 168,6667 |
| 148 | 144,8921 | 146,3034 | 148,5411 | 152,4978 | 157,2545 | 161,9521 | 164,2734 | 165,7712 | 167,8966 | 169,2244 |
| 149 | 145,3177 | 146,738 | 148,9896 | 152,9692 | 157,7514 | 162,473 | 164,8061 | 166,3116 | 168,4479 | 169,7824 |
| 150 | 145,7428 | 147,1721 | 149,4376 | 153,4403 | 158,2481 | 162,9937 | 165,3386 | 166,8518 | 168,999 | 170,3403 |
| 151 | 146,1671 | 147,6055 | 149,8849 | 153,9108 | 158,7442 | 163,5139 | 165,8707 | 167,3915 | 169,5496 | 170,8978 |
| 152 | 146,5903 | 148,0378 | 150,3311 | 154,3803 | 159,2394 | 164,0331 | 166,4018 | 167,9303 | 170,0993 | 171,4543 |
| 153 | 147,0119 | 148,4685 | 150,7759 | 154,8485 | 159,7333 | 164,5511 | 166,9317 | 168,4679 | 170,6478 | 172,0096 |
| 154 | 147,4317 | 148,8975 | 151,219 | 155,3149 | 160,2255 | 165,0674 | 167,4599 | 169,0038 | 171,1946 | 172,5633 |
| 155 | 147,8493 | 149,3244 | 151,6599 | 155,7794 | 160,7157 | 165,5817 | 167,9861 | 169,5377 | 171,7394 | 173,1149 |
| 156 | 148,2645 | 149,7487 | 152,0985 | 156,2414 | 161,2035 | 166,0937 | 168,5099 | 170,0692 | 172,2818 | 173,6641 |
| 157 | 148,6768 | 150,1703 | 152,5343 | 156,7007 | 161,6885 | 166,6028 | 169,031 | 170,5979 | 172,8214 | 174,2105 |
| 158 | 149,0858 | 150,5887 | 152,9668 | 157,1568 | 162,1704 | 167,1088 | 169,5487 | 171,1233 | 173,3577 | 174,7536 |
| 159 | 149,4911 | 151,0033 | 153,3957 | 157,6092 | 162,6486 | 167,6109 | 170,0627 | 171,645 | 173,8902 | 175,2929 |
| 160 | 149,8924 | 151,4139 | 153,8205 | 158,0576 | 163,1226 | 168,1089 | 170,5725 | 172,1624 | 174,4185 | 175,8279 |
| 161 | 150,289 | 151,8199 | 154,2408 | 158,5014 | 163,5921 | 168,6022 | 171,0776 | 172,675 | 174,9419 | 176,358 |
| 162 | 150,6807 | 152,2209 | 154,6561 | 158,9402 | 164,0564 | 169,0904 | 171,5774 | 173,1824 | 175,46 | 176,8828 |
| 163 | 151,0669 | 152,6165 | 155,0659 | 159,3735 | 164,5152 | 169,5728 | 172,0715 | 173,684 | 175,9723 | 177,4018 |
| 164 | 151,4473 | 153,0063 | 155,4699 | 159,8009 | 164,968 | 170,0491 | 172,5594 | 174,1794 | 176,4783 | 177,9145 |
| 165 | 151,8215 | 153,3897 | 155,8676 | 160,222 | 165,4143 | 170,5188 | 173,0406 | 174,668 | 176,9775 | 178,4203 |
| 166 | 152,1889 | 153,7665 | 156,2586 | 160,6362 | 165,8536 | 170,9814 | 173,5146 | 175,1494 | 177,4694 | 178,9187 |
| 167 | 152,5491 | 154,136 | 156,6423 | 161,0431 | 166,2855 | 171,4364 | 173,9809 | 175,6231 | 177,9535 | 179,4093 |
| 168 | 152,9018 | 154,498 | 157,0184 | 161,4423 | 166,7095 | 171,8833 | 174,439 | 176,0885 | 178,4292 | 179,8914 |
| 169 | 153,2465 | 154,852 | 157,3864 | 161,8333 | 167,1251 | 172,3216 | 174,8885 | 176,5452 | 178,8961 | 180,3647 |
| 170 | 153,5828 | 155,1975 | 157,746 | 162,2157 | 167,532 | 172,7509 | 175,3289 | 176,9927 | 179,3537 | 180,8287 |
| 171 | 153,9103 | 155,5342 | 158,0966 | 162,589 | 167,9295 | 173,1707 | 175,7596 | 177,4305 | 179,8016 | 181,2827 |
| 172 | 154,2286 | 155,8617 | 158,438 | 162,9529 | 168,3174 | 173,5806 | 176,1803 | 177,8581 | 180,2391 | 181,7265 |
| 173 | 154,5373 | 156,1795 | 158,7696 | 163,3069 | 168,6952 | 173,9801 | 176,5905 | 178,2752 | 180,666 | 182,1595 |
| 174 | 154,836 | 156,4873 | 159,091 | 163,6505 | 169,0623 | 174,3687 | 176,9897 | 178,6812 | 181,0817 | 182,5812 |
| 175 | 155,1243 | 156,7846 | 159,402 | 163,9835 | 169,4185 | 174,746 | 177,3774 | 179,0757 | 181,4857 | 182,9911 |
| 176 | 155,4018 | 157,071 | 159,7019 | 164,3052 | 169,7632 | 175,1116 | 177,7533 | 179,4581 | 181,8775 | 183,3888 |
| 177 | 155,6681 | 157,3462 | 159,9905 | 164,6154 | 170,096 | 175,465 | 178,1167 | 179,8281 | 182,2567 | 183,7738 |
| 178 | 155,9227 | 157,6097 | 160,2674 | 164,9136 | 170,4165 | 175,8058 | 178,4674 | 180,1852 | 182,6229 | 184,1456 |
| 179 | 156,1654 | 157,8612 | 160,532 | 165,1993 | 170,7243 | 176,1334 | 178,8047 | 180,5288 | 182,9755 | 184,5037 |
| 180 | 156,3958 | 158,1002 | 160,784 | 165,4722 | 171,0188 | 176,4474 | 179,1283 | 180,8586 | 183,314 | 184,8477 |
| 181 | 156,6134 | 158,3265 | 161,0232 | 165,7319 | 171,2998 | 176,7476 | 179,4379 | 181,1742 | 183,6382 | 185,1773 |
| 182 | 156,8187 | 158,5403 | 161,2498 | 165,9788 | 171,5676 | 177,0342 | 179,7337 | 181,4759 | 183,9483 | 185,4926 |
| 183 | 157,0121 | 158,7421 | 161,4642 | 166,2133 | 171,8227 | 177,3077 | 180,0162 | 181,7643 | 184,2449 | 185,7944 |
| 184 | 157,1939 | 158,9323 | 161,667 | 166,4359 | 172,0656 | 177,5686 | 180,286 | 182,0398 | 184,5285 | 186,083 |
| 185 | 157,3646 | 159,1114 | 161,8584 | 166,6469 | 172,2966 | 177,8175 | 180,5435 | 182,3029 | 184,7996 | 186,3591 |
| 186 | 157,5246 | 159,2797 | 162,039 | 166,8469 | 172,5163 | 178,0546 | 180,7892 | 182,5541 | 185,0586 | 186,623 |
| 187 | 157,6744 | 159,4376 | 162,2092 | 167,0363 | 172,7251 | 178,2806 | 181,0236 | 182,7939 | 185,3061 | 186,8752 |
| 188 | 157,8143 | 159,5857 | 162,3694 | 167,2155 | 172,9235 | 178,496 | 181,2472 | 183,0228 | 185,5426 | 187,1164 |
| 189 | 157,9447 | 159,7242 | 162,52 | 167,385 | 173,112 | 178,7011 | 181,4604 | 183,2413 | 185,7685 | 187,3469 |
| 190 | 158,0661 | 159,8538 | 162,6615 | 167,5453 | 173,2909 | 178,8964 | 181,6638 | 183,4499 | 185,9844 | 187,5674 |
| 191 | 158,1789 | 159,9746 | 162,7943 | 167,6966 | 173,4609 | 179,0826 | 181,8579 | 183,649 | 186,1907 | 187,7782 |
| 192 | 158,2835 | 160,0873 | 162,9188 | 167,8396 | 173,6222 | 179,2599 | 182,043 | 183,8392 | 186,388 | 187,9799 |
| 193 | 158,3804 | 160,1921 | 163,0356 | 167,9747 | 173,7755 | 179,429 | 182,2198 | 184,021 | 186,5768 | 188,1731 |
| 194 | 158,4701 | 160,2899 | 163,1451 | 168,1025 | 173,9213 | 179,5905 | 182,3889 | 184,195 | 186,7578 | 188,3584 |
| 195 | 158,5533 | 160,3811 | 163,2481 | 168,2237 | 174,0604 | 179,7451 | 182,551 | 184,3619 | 186,9316 | 188,5365 |
| 196 | 158,6307 | 160,4664 | 163,3452 | 168,3389 | 174,1934 | 179,8934 | 182,7068 | 184,5225 | 187,099 | 188,7081 |
| 197 | 158,7028 | 160,5465 | 163,437 | 168,4489 | 174,321 | 180,0362 | 182,857 | 184,6775 | 187,2607 | 188,874 |
| 198 | 158,7703 | 160,622 | 163,5243 | 168,5541 | 174,4438 | 180,1742 | 183,0023 | 184,8276 | 187,4175 | 189,0349 |
| 199 | 158,8339 | 160,6936 | 163,6076 | 168,6554 | 174,5626 | 180,308 | 183,1434 | 184,9734 | 187,5699 | 189,1915 |
| 200 | 158,8941 | 160,7618 | 163,6876 | 168,7534 | 174,678 | 180,4384 | 183,281 | 185,1156 | 187,7188 | 189,3444 |
| 201 | 158,9517 | 160,8274 | 163,7649 | 168,8487 | 174,7907 | 180,5659 | 183,4158 | 185,2551 | 187,8648 | 189,4945 |
| 202 | 159,0072 | 160,891 | 163,8403 | 168,9421 | 174,9014 | 180,6915 | 183,5485 | 185,3924 | 188,0087 | 189,6425 |
| 203 | 159,0613 | 160,9532 | 163,9142 | 169,0341 | 175,0108 | 180,8156 | 183,6798 | 185,5283 | 188,1511 | 189,789 |

**Supplementary Table 3.** **Centile reference values for female height**

| Months | 3th | 5th | 10th | 25th | 50th | 75th | 85th | 90th | 95th | 97th |
| --- | --- | --- | --- | --- | --- | --- | --- | --- | --- | --- |
| 72 | 111,2966 | 112,1106 | 113,4539 | 116,0196 | 119,3084 | 122,257 | 123,4682 | 124,1624 | 125,048 | 125,5517 |
| 73 | 111,795 | 112,608 | 113,9548 | 116,5451 | 119,8851 | 122,8795 | 124,1045 | 124,8039 | 125,6931 | 126,1973 |
| 74 | 112,2916 | 113,1037 | 114,4544 | 117,0694 | 120,4604 | 123,5007 | 124,7397 | 125,4445 | 126,3375 | 126,8424 |
| 75 | 112,7848 | 113,5965 | 114,9514 | 117,5913 | 121,0332 | 124,1193 | 125,3724 | 126,0828 | 126,98 | 127,4856 |
| 76 | 113,2734 | 114,0849 | 115,4445 | 118,1097 | 121,6022 | 124,7339 | 126,0013 | 126,7175 | 127,6191 | 128,1259 |
| 77 | 113,7559 | 114,5677 | 115,9325 | 118,6233 | 122,166 | 125,3433 | 126,6251 | 127,3473 | 128,2537 | 128,7619 |
| 78 | 114,231 | 115,0437 | 116,4141 | 119,1309 | 122,7234 | 125,9459 | 127,2425 | 127,9708 | 128,8825 | 129,3923 |
| 79 | 114,6974 | 115,5114 | 116,8882 | 119,6312 | 123,2732 | 126,5406 | 127,852 | 128,5867 | 129,5041 | 130,0159 |
| 80 | 115,1536 | 115,9696 | 117,3534 | 120,1231 | 123,814 | 127,1261 | 128,4524 | 129,1938 | 130,1173 | 130,6314 |
| 81 | 115,5985 | 116,417 | 117,8085 | 120,6054 | 124,3446 | 127,7008 | 129,0424 | 129,7906 | 130,7207 | 131,2376 |
| 82 | 116,0306 | 116,8523 | 118,2524 | 121,0767 | 124,8636 | 128,2636 | 129,6204 | 130,3759 | 131,3132 | 131,8332 |
| 83 | 116,4486 | 117,2743 | 118,6836 | 121,536 | 125,3699 | 128,8131 | 130,1854 | 130,9482 | 131,8932 | 132,4168 |
| 84 | 116,8511 | 117,6815 | 119,1012 | 121,9819 | 125,8621 | 129,3479 | 130,7358 | 131,5063 | 132,4597 | 132,9873 |
| 85 | 117,2373 | 118,0734 | 119,5043 | 122,4138 | 126,3395 | 129,8673 | 131,2709 | 132,0494 | 133,0118 | 133,5439 |
| 86 | 117,6092 | 118,4516 | 119,8947 | 122,8336 | 126,804 | 130,3731 | 131,7926 | 132,5794 | 133,5514 | 134,0886 |
| 87 | 117,9689 | 118,8184 | 120,2747 | 123,2434 | 127,2579 | 130,8678 | 132,3033 | 133,0987 | 134,081 | 134,6236 |
| 88 | 118,3189 | 119,1763 | 120,6466 | 123,6457 | 127,7035 | 131,3538 | 132,8054 | 133,6097 | 134,6029 | 135,1514 |
| 89 | 118,6614 | 119,5275 | 121,0127 | 124,0426 | 128,1433 | 131,8336 | 133,3015 | 134,115 | 135,1196 | 135,6745 |
| 90 | 118,9989 | 119,8744 | 121,3754 | 124,4366 | 128,5797 | 132,3095 | 133,7939 | 134,6168 | 135,6335 | 136,1954 |
| 91 | 119,3336 | 120,2192 | 121,7369 | 124,83 | 129,0149 | 132,7839 | 134,2851 | 135,1178 | 136,1472 | 136,7164 |
| 92 | 119,6678 | 120,5642 | 122,0995 | 125,225 | 129,4514 | 133,2594 | 134,7774 | 135,6203 | 136,6631 | 137,24 |
| 93 | 120,0039 | 120,9118 | 122,4655 | 125,6241 | 129,8916 | 133,7383 | 135,2735 | 136,1268 | 137,1835 | 137,7688 |
| 94 | 120,3441 | 121,2643 | 122,8373 | 126,0295 | 130,3378 | 134,2231 | 135,7757 | 136,6397 | 137,7111 | 138,3051 |
| 95 | 120,6906 | 121,6239 | 123,2172 | 126,4436 | 130,7924 | 134,7161 | 136,2864 | 137,1615 | 138,2483 | 138,8516 |
| 96 | 121,0459 | 121,9929 | 123,6073 | 126,8688 | 131,2578 | 135,2198 | 136,8081 | 137,6947 | 138,7975 | 139,4105 |
| 97 | 121,4116 | 122,3731 | 124,0096 | 127,3067 | 131,7358 | 135,736 | 137,3426 | 138,2411 | 139,3606 | 139,984 |
| 98 | 121,7878 | 122,7645 | 124,424 | 127,7574 | 132,2262 | 136,2644 | 137,8897 | 138,8004 | 139,9373 | 140,5714 |
| 99 | 122,174 | 123,1666 | 124,85 | 128,2202 | 132,7281 | 136,8039 | 138,4481 | 139,3714 | 140,5264 | 141,1718 |
| 100 | 122,5697 | 123,579 | 125,2869 | 128,6943 | 133,2406 | 137,3537 | 139,017 | 139,9532 | 141,127 | 141,7842 |
| 101 | 122,9745 | 124,001 | 125,7343 | 129,1793 | 133,7631 | 137,9127 | 139,5953 | 140,5447 | 141,7379 | 142,4074 |
| 102 | 123,3879 | 124,4323 | 126,1917 | 129,6744 | 134,2947 | 138,48 | 140,182 | 141,1449 | 142,3582 | 143,0405 |
| 103 | 123,8094 | 124,8723 | 126,6584 | 130,1789 | 134,8345 | 139,0547 | 140,7761 | 141,7528 | 142,9868 | 143,6824 |
| 104 | 124,2386 | 125,3206 | 127,1341 | 130,6924 | 135,3817 | 139,6356 | 141,3766 | 142,3674 | 143,6226 | 144,332 |
| 105 | 124,6751 | 125,7768 | 127,6182 | 131,2141 | 135,9356 | 140,2219 | 141,9824 | 142,9874 | 144,2645 | 144,9882 |
| 106 | 125,1184 | 126,2403 | 128,1101 | 131,7434 | 136,4953 | 140,8125 | 142,5925 | 143,612 | 144,9115 | 145,6499 |
| 107 | 125,5681 | 126,7108 | 128,6094 | 132,2797 | 137,0599 | 141,4066 | 143,2059 | 144,2401 | 145,5626 | 146,3161 |
| 108 | 126,0239 | 127,1877 | 129,1155 | 132,8223 | 137,6287 | 142,003 | 143,8216 | 144,8706 | 146,2165 | 146,9857 |
| 109 | 126,4853 | 127,6707 | 129,628 | 133,3707 | 138,2009 | 142,6009 | 144,4385 | 145,5024 | 146,8722 | 147,6575 |
| 110 | 126,9517 | 128,1591 | 130,1462 | 133,9241 | 138,7757 | 143,1993 | 145,0557 | 146,1347 | 147,5289 | 148,3307 |
| 111 | 127,4225 | 128,6523 | 130,6695 | 134,4819 | 139,3523 | 143,7975 | 145,6725 | 146,7667 | 148,1858 | 149,0045 |
| 112 | 127,8969 | 129,1496 | 131,1973 | 135,0435 | 139,93 | 144,3946 | 146,2879 | 147,3974 | 148,8419 | 149,6781 |
| 113 | 128,3743 | 129,6505 | 131,729 | 135,6082 | 140,5082 | 144,9898 | 146,901 | 148,026 | 149,4964 | 150,3506 |
| 114 | 128,8542 | 130,1543 | 132,2638 | 136,1754 | 141,0859 | 145,5821 | 147,5111 | 148,6516 | 150,1485 | 151,0212 |
| 115 | 129,3358 | 130,6603 | 132,8013 | 136,7445 | 141,6625 | 146,1708 | 148,1172 | 149,2734 | 150,7973 | 151,689 |
| 116 | 129,8185 | 131,168 | 133,3409 | 137,3148 | 142,2372 | 146,7549 | 148,7185 | 149,8905 | 151,4419 | 152,3532 |
| 117 | 130,3017 | 131,6767 | 133,8818 | 137,8856 | 142,8093 | 147,3337 | 149,314 | 150,502 | 152,0815 | 153,013 |
| 118 | 130,7848 | 132,1859 | 134,4235 | 138,4564 | 143,3781 | 147,9063 | 149,903 | 151,107 | 152,7153 | 153,6675 |
| 119 | 131,267 | 132,6949 | 134,9655 | 139,0264 | 143,9427 | 148,4718 | 150,4845 | 151,7046 | 153,3422 | 154,3159 |
| 120 | 131,7478 | 133,2031 | 135,507 | 139,5952 | 144,5025 | 149,0293 | 151,0576 | 152,294 | 153,9616 | 154,9573 |
| 121 | 132,2266 | 133,7099 | 136,0475 | 140,162 | 145,0567 | 149,5781 | 151,6215 | 152,8743 | 154,5725 | 155,591 |
| 122 | 132,7028 | 134,2148 | 136,5865 | 140,7262 | 145,6049 | 150,1177 | 152,1758 | 153,445 | 155,1744 | 156,2163 |
| 123 | 133,1758 | 134,7171 | 137,1233 | 141,2874 | 146,1465 | 150,6476 | 152,72 | 154,0057 | 155,7669 | 156,8329 |
| 124 | 133,6451 | 135,2163 | 137,6573 | 141,8448 | 146,6811 | 151,1675 | 153,2536 | 154,5559 | 156,3496 | 157,4404 |
| 125 | 134,1101 | 135,7118 | 138,188 | 142,3979 | 147,2081 | 151,677 | 153,7764 | 155,0953 | 156,922 | 158,0382 |
| 126 | 134,5704 | 136,2032 | 138,7146 | 142,946 | 147,7272 | 152,1757 | 154,288 | 155,6235 | 157,4836 | 158,6258 |
| 127 | 135,0255 | 136,6897 | 139,2366 | 143,4885 | 148,2379 | 152,6634 | 154,788 | 156,1401 | 158,034 | 159,2027 |
| 128 | 135,475 | 137,171 | 139,7533 | 144,0246 | 148,7396 | 153,1396 | 155,2761 | 156,6446 | 158,5727 | 159,7685 |
| 129 | 135,9185 | 137,6465 | 140,2639 | 144,5536 | 149,2319 | 153,6043 | 155,752 | 157,1369 | 159,0993 | 160,3225 |
| 130 | 136,3556 | 138,1155 | 140,7678 | 145,0747 | 149,7143 | 154,0571 | 156,2155 | 157,6165 | 159,6133 | 160,8641 |
| 131 | 136,7859 | 138,5777 | 141,2642 | 145,587 | 150,1864 | 154,498 | 156,6664 | 158,0833 | 160,1143 | 161,3928 |
| 132 | 137,2092 | 139,0323 | 141,7523 | 146,0897 | 150,6477 | 154,9267 | 157,1045 | 158,5369 | 160,6017 | 161,908 |
| 133 | 137,6252 | 139,479 | 142,2314 | 146,582 | 151,0977 | 155,3434 | 157,5299 | 158,9772 | 161,0753 | 162,4091 |
| 134 | 138,0341 | 139,9178 | 142,7014 | 147,0636 | 151,5367 | 155,7482 | 157,9427 | 159,4045 | 161,5352 | 162,8962 |
| 135 | 138,436 | 140,3487 | 143,1622 | 147,5348 | 151,9647 | 156,1414 | 158,3433 | 159,8191 | 161,9816 | 163,3693 |
| 136 | 138,831 | 140,7719 | 143,6139 | 147,9953 | 152,3819 | 156,5235 | 158,7321 | 160,2211 | 162,4147 | 163,8287 |
| 137 | 139,2195 | 141,1873 | 144,0565 | 148,4454 | 152,7885 | 156,8948 | 159,1093 | 160,6111 | 162,8347 | 164,2746 |
| 138 | 139,6014 | 141,5952 | 144,4901 | 148,8849 | 153,1848 | 157,2555 | 159,4752 | 160,9892 | 163,242 | 164,707 |
| 139 | 139,9768 | 141,9954 | 144,9146 | 149,314 | 153,5707 | 157,6059 | 159,8302 | 161,3558 | 163,6367 | 165,1263 |
| 140 | 140,346 | 142,3882 | 145,3301 | 149,7327 | 153,9467 | 157,9465 | 160,1747 | 161,7111 | 164,0191 | 165,5326 |
| 141 | 140,7089 | 142,7734 | 145,7365 | 150,141 | 154,3128 | 158,2774 | 160,5088 | 162,0555 | 164,3896 | 165,9261 |
| 142 | 141,0656 | 143,1512 | 146,1339 | 150,5391 | 154,6692 | 158,5989 | 160,833 | 162,3893 | 164,7483 | 166,3072 |
| 143 | 141,4161 | 143,5216 | 146,5224 | 150,927 | 155,016 | 158,9113 | 161,1474 | 162,7128 | 165,0955 | 166,676 |
| 144 | 141,7605 | 143,8845 | 146,902 | 151,3048 | 155,3536 | 159,2149 | 161,4525 | 163,0262 | 165,4316 | 167,0329 |
| 145 | 142,0988 | 144,24 | 147,2727 | 151,6727 | 155,6819 | 159,5098 | 161,7484 | 163,3299 | 165,7568 | 167,378 |
| 146 | 142,4309 | 144,5881 | 147,6344 | 152,0305 | 156,0012 | 159,7965 | 162,0355 | 163,6242 | 166,0714 | 167,7116 |
| 147 | 142,7569 | 144,9286 | 147,987 | 152,3782 | 156,3116 | 160,0751 | 162,3141 | 163,9093 | 166,3756 | 168,0338 |
| 148 | 143,0768 | 145,2614 | 148,3302 | 152,7157 | 156,613 | 160,3459 | 162,5844 | 164,1856 | 166,6695 | 168,3448 |
| 149 | 143,3905 | 145,5865 | 148,6641 | 153,0429 | 156,9058 | 160,6093 | 162,8469 | 164,4534 | 166,9536 | 168,6447 |
| 150 | 143,698 | 145,9037 | 148,9883 | 153,3596 | 157,1898 | 160,8654 | 163,1018 | 164,7129 | 167,2281 | 168,9337 |
| 151 | 143,9991 | 146,2129 | 149,3028 | 153,6659 | 157,4653 | 161,1146 | 163,3495 | 164,9646 | 167,4931 | 169,2121 |
| 152 | 144,294 | 146,514 | 149,6075 | 153,9617 | 157,7322 | 161,3571 | 163,5902 | 165,2086 | 167,749 | 169,48 |
| 153 | 144,5824 | 146,807 | 149,9022 | 154,2469 | 157,9908 | 161,5932 | 163,8242 | 165,4454 | 167,996 | 169,7376 |
| 154 | 144,8644 | 147,0916 | 150,1868 | 154,5214 | 158,2412 | 161,8231 | 164,0518 | 165,6751 | 168,2344 | 169,9851 |
| 155 | 145,1399 | 147,3678 | 150,4612 | 154,7854 | 158,4833 | 162,0469 | 164,2733 | 165,8982 | 168,4645 | 170,2228 |
| 156 | 145,4087 | 147,6355 | 150,7254 | 155,0386 | 158,7173 | 162,265 | 164,4889 | 166,1147 | 168,6864 | 170,4508 |
| 157 | 145,6709 | 147,8947 | 150,9793 | 155,2812 | 158,9434 | 162,4775 | 164,6989 | 166,3251 | 168,9005 | 170,6695 |
| 158 | 145,9266 | 148,1455 | 151,2231 | 155,5134 | 159,1617 | 162,6846 | 164,9035 | 166,5294 | 169,107 | 170,8789 |
| 159 | 146,1758 | 148,3881 | 151,457 | 155,7354 | 159,3723 | 162,8866 | 165,1028 | 166,728 | 169,3061 | 171,0795 |
| 160 | 146,4186 | 148,6225 | 151,6811 | 155,9474 | 159,5756 | 163,0834 | 165,2971 | 166,921 | 169,4981 | 171,2715 |
| 161 | 146,6551 | 148,849 | 151,8957 | 156,1496 | 159,7716 | 163,2754 | 165,4865 | 167,1086 | 169,6831 | 171,455 |
| 162 | 146,8856 | 149,0678 | 152,1011 | 156,3424 | 159,9606 | 163,4626 | 165,6711 | 167,291 | 169,8615 | 171,6305 |
| 163 | 147,11 | 149,279 | 152,2975 | 156,526 | 160,1428 | 163,6451 | 165,8511 | 167,4682 | 170,0333 | 171,798 |
| 164 | 147,3286 | 149,483 | 152,4853 | 156,7007 | 160,3183 | 163,8231 | 166,0265 | 167,6405 | 170,1989 | 171,958 |
| 165 | 147,5414 | 149,6798 | 152,6647 | 156,8669 | 160,4874 | 163,9967 | 166,1975 | 167,8079 | 170,3582 | 172,1105 |
| 166 | 147,7486 | 149,8697 | 152,8361 | 157,0247 | 160,6503 | 164,1659 | 166,3642 | 167,9706 | 170,5117 | 172,2559 |
| 167 | 147,9503 | 150,0531 | 152,9997 | 157,1746 | 160,8071 | 164,3308 | 166,5266 | 168,1286 | 170,6593 | 172,3944 |
| 168 | 148,1467 | 150,23 | 153,1558 | 157,3169 | 160,958 | 164,4916 | 166,6849 | 168,282 | 170,8013 | 172,5263 |
| 169 | 148,3378 | 150,4007 | 153,3049 | 157,4519 | 161,1033 | 164,6482 | 166,839 | 168,431 | 170,9379 | 172,6518 |
| 170 | 148,5239 | 150,5657 | 153,4472 | 157,5799 | 161,2432 | 164,8009 | 166,9891 | 168,5757 | 171,0692 | 172,7711 |
| 171 | 148,7053 | 150,725 | 153,5832 | 157,7013 | 161,3779 | 164,9498 | 167,1354 | 168,7163 | 171,1954 | 172,8846 |
| 172 | 148,8822 | 150,8792 | 153,7132 | 157,8165 | 161,5077 | 165,095 | 167,278 | 168,8528 | 171,3169 | 172,9925 |
| 173 | 149,0548 | 151,0285 | 153,8377 | 157,926 | 161,6329 | 165,2367 | 167,4169 | 168,9854 | 171,4337 | 173,0951 |
| 174 | 149,2233 | 151,1732 | 153,9569 | 158,0299 | 161,7537 | 165,375 | 167,5524 | 169,1143 | 171,5461 | 173,1927 |
| 175 | 149,388 | 151,3135 | 154,0714 | 158,1288 | 161,8703 | 165,51 | 167,6846 | 169,2397 | 171,6543 | 173,2855 |
| 176 | 149,5491 | 151,4499 | 154,1813 | 158,223 | 161,983 | 165,642 | 167,8136 | 169,3616 | 171,7585 | 173,3739 |
| 177 | 149,7067 | 151,5826 | 154,2872 | 158,3128 | 162,0921 | 165,771 | 167,9396 | 169,4802 | 171,859 | 173,4581 |
| 178 | 149,8612 | 151,7119 | 154,3893 | 158,3987 | 162,1978 | 165,8972 | 168,0627 | 169,5958 | 171,9559 | 173,5384 |
| 179 | 150,0127 | 151,838 | 154,488 | 158,4809 | 162,3004 | 166,0207 | 168,183 | 169,7085 | 172,0495 | 173,6151 |
| 180 | 150,1614 | 151,9613 | 154,5836 | 158,5598 | 162,4001 | 166,1418 | 168,3008 | 169,8185 | 172,1401 | 173,6886 |
| 181 | 150,3075 | 152,082 | 154,6765 | 158,6358 | 162,4972 | 166,2606 | 168,4162 | 169,9259 | 172,2279 | 173,759 |
| 182 | 150,4513 | 152,2002 | 154,7668 | 158,709 | 162,5918 | 166,3772 | 168,5293 | 170,0308 | 172,313 | 173,8266 |
| 183 | 150,5927 | 152,3162 | 154,8548 | 158,7797 | 162,6841 | 166,4917 | 168,6402 | 170,1335 | 172,3956 | 173,8915 |
| 184 | 150,732 | 152,4301 | 154,9406 | 158,8481 | 162,7742 | 166,6043 | 168,749 | 170,234 | 172,4758 | 173,9539 |
| 185 | 150,8693 | 152,5421 | 155,0245 | 158,9144 | 162,8624 | 166,7149 | 168,8559 | 170,3325 | 172,5539 | 174,0141 |
| 186 | 151,0047 | 152,6524 | 155,1067 | 158,9787 | 162,9487 | 166,8239 | 168,961 | 170,429 | 172,6298 | 174,0722 |
| 187 | 151,1384 | 152,761 | 155,1872 | 159,0414 | 163,0334 | 166,9312 | 169,0644 | 170,5237 | 172,7039 | 174,1283 |
| 188 | 151,2704 | 152,8682 | 155,2664 | 159,1025 | 163,1165 | 167,037 | 169,1661 | 170,6168 | 172,7762 | 174,1827 |
| 189 | 151,401 | 152,9741 | 155,3444 | 159,1624 | 163,1983 | 167,1415 | 169,2665 | 170,7083 | 172,847 | 174,2356 |
| 190 | 151,5302 | 153,0789 | 155,4213 | 159,2211 | 163,2788 | 167,2446 | 169,3654 | 170,7985 | 172,9164 | 174,287 |
| 191 | 151,6583 | 153,1827 | 155,4974 | 159,2789 | 163,3584 | 167,3466 | 169,4632 | 170,8874 | 172,9845 | 174,3373 |
| 192 | 151,7852 | 153,2856 | 155,5729 | 159,336 | 163,437 | 167,4477 | 169,5599 | 170,9752 | 173,0515 | 174,3866 |
| 193 | 151,9112 | 153,3879 | 155,6478 | 159,3925 | 163,5149 | 167,5478 | 169,6556 | 171,0621 | 173,1176 | 174,4351 |
| 194 | 152,0361 | 153,4895 | 155,7224 | 159,4487 | 163,5922 | 167,647 | 169,7504 | 171,1479 | 173,1828 | 174,4828 |
| 195 | 152,1601 | 153,5906 | 155,7966 | 159,5046 | 163,6688 | 167,7452 | 169,8442 | 171,2329 | 173,2473 | 174,53 |
| 196 | 152,2832 | 153,691 | 155,8707 | 159,5605 | 163,7449 | 167,8425 | 169,937 | 171,3169 | 173,3111 | 174,5769 |
| 197 | 152,4052 | 153,791 | 155,9448 | 159,6164 | 163,8205 | 167,9388 | 170,0289 | 171,4001 | 173,3744 | 174,6234 |
| 198 | 152,5263 | 153,8905 | 156,0188 | 159,6724 | 163,8957 | 168,0342 | 170,1198 | 171,4824 | 173,4371 | 174,6697 |
| 199 | 152,6464 | 153,9896 | 156,0929 | 159,7287 | 163,9705 | 168,1286 | 170,2098 | 171,5639 | 173,4994 | 174,716 |
| 200 | 152,7656 | 154,0883 | 156,1672 | 159,7853 | 164,045 | 168,2221 | 170,299 | 171,6447 | 173,5614 | 174,7624 |
| 201 | 152,8839 | 154,1866 | 156,2417 | 159,8424 | 164,1193 | 168,3147 | 170,3872 | 171,7248 | 173,6231 | 174,8089 |
| 202 | 153,0013 | 154,2847 | 156,3165 | 159,9001 | 164,1934 | 168,4065 | 170,4747 | 171,8043 | 173,6846 | 174,8556 |
| 203 | 153,1179 | 154,3825 | 156,3916 | 159,9584 | 164,2674 | 168,4974 | 170,5614 | 171,8832 | 173,746 | 174,9026 |

**Supplementary Table 4.** **Centile reference values for male weight**

| Months | 3th | 5th | 10th | 25th | 50th | 75th | 85th | 90th | 95th | 97th |
| --- | --- | --- | --- | --- | --- | --- | --- | --- | --- | --- |
| 72 | 18,55962 | 18,88797 | 19,49188 | 20,90438 | 23,2666 | 25,94157 | 27,17806 | 27,90832 | 28,85612 | 29,40237 |
| 73 | 18,74915 | 19,08431 | 19,70013 | 21,13786 | 23,53882 | 26,25685 | 27,51411 | 28,2574 | 29,22321 | 29,78043 |
| 74 | 18,93821 | 19,28029 | 19,9082 | 21,37142 | 23,81135 | 26,57266 | 27,85083 | 28,60727 | 29,59128 | 30,1596 |
| 75 | 19,12708 | 19,47619 | 20,11635 | 21,60534 | 24,0845 | 26,88934 | 28,18855 | 28,95826 | 29,96067 | 30,54024 |
| 76 | 19,31603 | 19,67229 | 20,32488 | 21,83992 | 24,35859 | 27,20719 | 28,52761 | 29,31071 | 30,33172 | 30,92269 |
| 77 | 19,50532 | 19,86884 | 20,53406 | 22,07547 | 24,63394 | 27,52655 | 28,86834 | 29,66494 | 30,70477 | 31,30727 |
| 78 | 19,69522 | 20,06614 | 20,74418 | 22,31226 | 24,91084 | 27,84775 | 29,21105 | 30,0213 | 31,08015 | 31,69434 |
| 79 | 19,88601 | 20,26444 | 20,95551 | 22,55061 | 25,18963 | 28,17109 | 29,55609 | 30,3801 | 31,4582 | 32,08423 |
| 80 | 20,07793 | 20,46403 | 21,16835 | 22,7908 | 25,4706 | 28,49692 | 29,90378 | 30,74169 | 31,83925 | 32,47727 |
| 81 | 20,27126 | 20,66516 | 21,38295 | 23,03315 | 25,75407 | 28,82555 | 30,25444 | 31,10639 | 32,22364 | 32,8738 |
| 82 | 20,46627 | 20,86813 | 21,59962 | 23,27793 | 26,04036 | 29,1573 | 30,6084 | 31,47453 | 32,61169 | 33,27416 |
| 83 | 20,66322 | 21,07318 | 21,81863 | 23,52546 | 26,32978 | 29,4925 | 30,96599 | 31,84644 | 33,00374 | 33,67869 |
| 84 | 20,86237 | 21,28061 | 22,04026 | 23,77602 | 26,62264 | 29,83147 | 31,32753 | 32,22245 | 33,40012 | 34,0877 |
| 85 | 21,06395 | 21,49062 | 22,26475 | 24,02988 | 26,9192 | 30,17446 | 31,6933 | 32,60281 | 33,8011 | 34,50147 |
| 86 | 21,26799 | 21,70327 | 22,49214 | 24,28707 | 27,2195 | 30,52153 | 32,06331 | 32,98758 | 34,20672 | 34,92005 |
| 87 | 21,47448 | 21,91855 | 22,72243 | 24,54759 | 27,52354 | 30,87265 | 32,43756 | 33,37672 | 34,61694 | 35,3434 |
| 88 | 21,68342 | 22,13645 | 22,95562 | 24,81146 | 27,83132 | 31,22781 | 32,81603 | 33,77021 | 35,03176 | 35,7715 |
| 89 | 21,89481 | 22,35698 | 23,19171 | 25,07866 | 28,14282 | 31,58698 | 33,19868 | 34,16804 | 35,45113 | 36,20433 |
| 90 | 22,10864 | 22,58012 | 23,4307 | 25,34921 | 28,45805 | 31,95015 | 33,58551 | 34,57017 | 35,87505 | 36,64187 |
| 91 | 22,3249 | 22,80589 | 23,6726 | 25,62309 | 28,77698 | 32,31731 | 33,97648 | 34,97659 | 36,30348 | 37,08408 |
| 92 | 22,54359 | 23,03427 | 23,91739 | 25,90033 | 29,09962 | 32,68842 | 34,37158 | 35,38726 | 36,7364 | 37,53094 |
| 93 | 22,76472 | 23,26527 | 24,1651 | 26,1809 | 29,42596 | 33,06348 | 34,77078 | 35,80217 | 37,17378 | 37,98244 |
| 94 | 22,98827 | 23,49889 | 24,41571 | 26,46483 | 29,75599 | 33,44247 | 35,17406 | 36,22129 | 37,6156 | 38,43853 |
| 95 | 23,21425 | 23,73514 | 24,66924 | 26,75211 | 30,08971 | 33,82535 | 35,58139 | 36,64459 | 38,06182 | 38,89919 |
| 96 | 23,44266 | 23,974 | 24,92569 | 27,04275 | 30,42711 | 34,21212 | 35,99276 | 37,07206 | 38,51243 | 39,3644 |
| 97 | 23,67345 | 24,21544 | 25,18501 | 27,33671 | 30,76814 | 34,60273 | 36,4081 | 37,50363 | 38,96737 | 39,83411 |
| 98 | 23,90636 | 24,45921 | 25,44697 | 27,63377 | 31,11261 | 34,997 | 36,82727 | 37,93916 | 39,42652 | 40,30821 |
| 99 | 24,14111 | 24,70502 | 25,71129 | 27,93366 | 31,46027 | 35,39472 | 37,25009 | 38,37849 | 39,88974 | 40,78656 |
| 100 | 24,37738 | 24,95258 | 25,97769 | 28,23612 | 31,8109 | 35,79571 | 37,67637 | 38,82147 | 40,35689 | 41,26906 |
| 101 | 24,61486 | 25,20157 | 26,24586 | 28,54087 | 32,16424 | 36,19977 | 38,10596 | 39,26794 | 40,82785 | 41,75557 |
| 102 | 24,85324 | 25,45169 | 26,51552 | 28,84766 | 32,52008 | 36,6067 | 38,53868 | 39,71775 | 41,30249 | 42,24601 |
| 103 | 25,0922 | 25,70263 | 26,78637 | 29,1562 | 32,87816 | 37,01631 | 38,97438 | 40,17075 | 41,78069 | 42,74028 |
| 104 | 25,3314 | 25,95407 | 27,0581 | 29,46623 | 33,23825 | 37,42842 | 39,41288 | 40,62681 | 42,26234 | 43,23827 |
| 105 | 25,57053 | 26,20569 | 27,3304 | 29,77745 | 33,60012 | 37,84283 | 39,85404 | 41,08579 | 42,74734 | 43,7399 |
| 106 | 25,80923 | 26,45715 | 27,60297 | 30,0896 | 33,96352 | 38,25937 | 40,2977 | 41,54756 | 43,2356 | 44,24511 |
| 107 | 26,04718 | 26,70814 | 27,8755 | 30,40239 | 34,32822 | 38,67786 | 40,74373 | 42,012 | 43,72701 | 44,75381 |
| 108 | 26,28401 | 26,95831 | 28,14765 | 30,71554 | 34,69398 | 39,09811 | 41,19196 | 42,47898 | 44,2215 | 45,26595 |
| 109 | 26,5194 | 27,20733 | 28,41913 | 31,02875 | 35,06055 | 39,51991 | 41,64221 | 42,94833 | 44,71891 | 45,78139 |
| 110 | 26,75311 | 27,45498 | 28,68969 | 31,34177 | 35,42759 | 39,94285 | 42,09404 | 43,41959 | 45,21876 | 46,29964 |
| 111 | 26,98495 | 27,70104 | 28,9591 | 31,65431 | 35,79475 | 40,36649 | 42,54696 | 43,89224 | 45,72051 | 46,82013 |
| 112 | 27,21473 | 27,94531 | 29,22714 | 31,96612 | 36,16168 | 40,79036 | 43,00047 | 44,36576 | 46,22359 | 47,34229 |
| 113 | 27,44224 | 28,1876 | 29,4936 | 32,27692 | 36,52802 | 41,21402 | 43,45406 | 44,83962 | 46,72744 | 47,86552 |
| 114 | 27,66732 | 28,42772 | 29,75828 | 32,58647 | 36,89343 | 41,637 | 43,90724 | 45,31329 | 47,23149 | 48,38924 |
| 115 | 27,88978 | 28,66547 | 30,02096 | 32,8945 | 37,25754 | 42,05885 | 44,35949 | 45,78622 | 47,73516 | 48,91285 |
| 116 | 28,10947 | 28,90069 | 30,28144 | 33,20076 | 37,62002 | 42,4791 | 44,81029 | 46,25787 | 48,23786 | 49,43573 |
| 117 | 28,32623 | 29,1332 | 30,53954 | 33,505 | 37,9805 | 42,89729 | 45,25913 | 46,72769 | 48,739 | 49,95727 |
| 118 | 28,5399 | 29,36285 | 30,79506 | 33,80696 | 38,33863 | 43,31295 | 45,7055 | 47,19513 | 49,23799 | 50,47685 |
| 119 | 28,75035 | 29,58946 | 31,04781 | 34,1064 | 38,69407 | 43,72561 | 46,14886 | 47,65964 | 49,73423 | 50,99384 |
| 120 | 28,95743 | 29,81291 | 31,29763 | 34,40308 | 39,04646 | 44,13479 | 46,58868 | 48,12064 | 50,22711 | 51,50761 |
| 121 | 29,16117 | 30,03317 | 31,54449 | 34,6969 | 39,39557 | 44,54015 | 47,02456 | 48,57768 | 50,71611 | 52,01761 |
| 122 | 29,3622 | 30,25087 | 31,78893 | 34,98832 | 39,74173 | 44,94182 | 47,45653 | 49,03076 | 51,20115 | 52,52371 |
| 123 | 29,56128 | 30,46675 | 32,03169 | 35,27797 | 40,08538 | 45,34005 | 47,88474 | 49,47995 | 51,68222 | 53,02585 |
| 124 | 29,75923 | 30,6816 | 32,27349 | 35,56645 | 40,42698 | 45,73507 | 48,30935 | 49,92533 | 52,15931 | 53,52395 |
| 125 | 29,95686 | 30,8962 | 32,51506 | 35,85441 | 40,76695 | 46,12714 | 48,73047 | 50,36699 | 52,6324 | 54,01793 |
| 126 | 30,15501 | 31,11136 | 32,75717 | 36,14247 | 41,10576 | 46,51649 | 49,14825 | 50,80496 | 53,10144 | 54,5077 |
| 127 | 30,35452 | 31,32789 | 33,00057 | 36,43127 | 41,44386 | 46,90334 | 49,5628 | 51,2393 | 53,56638 | 54,99312 |
| 128 | 30,55626 | 31,54663 | 33,24603 | 36,72146 | 41,78168 | 47,28792 | 49,97424 | 51,67005 | 54,02715 | 55,47408 |
| 129 | 30,76111 | 31,76843 | 33,49434 | 37,01368 | 42,11968 | 47,67046 | 50,38266 | 52,09724 | 54,48367 | 55,95041 |
| 130 | 30,97 | 31,99416 | 33,7463 | 37,30859 | 42,4583 | 48,05116 | 50,78817 | 52,52089 | 54,93586 | 56,42197 |
| 131 | 31,18383 | 32,2247 | 34,00273 | 37,60686 | 42,79799 | 48,43025 | 51,19086 | 52,941 | 55,3836 | 56,88855 |
| 132 | 31,40355 | 32,46095 | 34,26444 | 37,90915 | 43,13919 | 48,80792 | 51,5908 | 53,35758 | 55,82677 | 57,34998 |
| 133 | 31,62999 | 32,7037 | 34,53215 | 38,21603 | 43,4823 | 49,18436 | 51,98807 | 53,77063 | 56,26527 | 57,80607 |
| 134 | 31,86341 | 32,95318 | 34,80606 | 38,52766 | 43,82741 | 49,55963 | 52,38273 | 54,18018 | 56,69911 | 58,25683 |
| 135 | 32,10391 | 33,20949 | 35,08626 | 38,84409 | 44,17457 | 49,93379 | 52,77483 | 54,5863 | 57,12834 | 58,7023 |
| 136 | 32,35161 | 33,47273 | 35,37283 | 39,16538 | 44,52383 | 50,3069 | 53,16442 | 54,98903 | 57,55302 | 59,14254 |
| 137 | 32,60662 | 33,74301 | 35,66583 | 39,49156 | 44,87521 | 50,679 | 53,55157 | 55,38843 | 57,9732 | 59,57758 |
| 138 | 32,86906 | 34,02042 | 35,96536 | 39,8227 | 45,22877 | 51,05014 | 53,93632 | 55,78457 | 58,38894 | 60,00748 |
| 139 | 33,13903 | 34,30505 | 36,27149 | 40,15884 | 45,58454 | 51,4204 | 54,31875 | 56,1775 | 58,80028 | 60,43229 |
| 140 | 33,41665 | 34,597 | 36,58428 | 40,50002 | 45,94257 | 51,78982 | 54,69891 | 56,56728 | 59,2073 | 60,85207 |
| 141 | 33,70202 | 34,89637 | 36,90382 | 40,84629 | 46,30289 | 52,15846 | 55,07687 | 56,95399 | 59,61005 | 61,26688 |
| 142 | 33,99525 | 35,20326 | 37,23017 | 41,19769 | 46,66556 | 52,52638 | 55,45269 | 57,33767 | 60,00858 | 61,67677 |
| 143 | 34,29646 | 35,51774 | 37,56341 | 41,55427 | 47,03061 | 52,89364 | 55,82643 | 57,7184 | 60,40297 | 62,08179 |
| 144 | 34,60575 | 35,83992 | 37,9036 | 41,91607 | 47,39808 | 53,2603 | 56,19817 | 58,09625 | 60,79327 | 62,48201 |
| 145 | 34,92307 | 36,16974 | 38,25067 | 42,28299 | 47,7679 | 53,62632 | 56,56787 | 58,47119 | 61,17948 | 62,87741 |
| 146 | 35,24781 | 36,50658 | 38,60398 | 42,65442 | 48,13951 | 53,99126 | 56,93513 | 58,84284 | 61,56123 | 63,26767 |
| 147 | 35,57918 | 36,84964 | 38,96277 | 43,02962 | 48,51227 | 54,35456 | 57,29947 | 59,21076 | 61,93815 | 63,65241 |
| 148 | 35,91637 | 37,19813 | 39,32622 | 43,40781 | 48,88549 | 54,71568 | 57,6604 | 59,57451 | 62,30982 | 64,03127 |
| 149 | 36,25856 | 37,55121 | 39,69353 | 43,78823 | 49,25851 | 55,07411 | 58,01747 | 59,93365 | 62,67589 | 64,40393 |
| 150 | 36,6049 | 37,90803 | 40,06386 | 44,1701 | 49,63068 | 55,42931 | 58,37021 | 60,28778 | 63,036 | 64,77005 |
| 151 | 36,95449 | 38,26774 | 40,43636 | 44,55263 | 50,00132 | 55,78077 | 58,71818 | 60,6365 | 63,38982 | 65,12936 |
| 152 | 37,30644 | 38,62943 | 40,81017 | 44,93503 | 50,36977 | 56,12798 | 59,06097 | 60,97944 | 63,73704 | 65,48159 |
| 153 | 37,65982 | 38,9922 | 41,1844 | 45,31648 | 50,73536 | 56,47044 | 59,39814 | 61,31623 | 64,07737 | 65,82647 |
| 154 | 38,01367 | 39,3551 | 41,55816 | 45,69618 | 51,09743 | 56,80767 | 59,7293 | 61,64652 | 64,41054 | 66,1638 |
| 155 | 38,36701 | 39,71717 | 41,93051 | 46,0733 | 51,45531 | 57,13919 | 60,05406 | 61,96997 | 64,73628 | 66,49337 |
| 156 | 38,71883 | 40,07743 | 42,30053 | 46,447 | 51,80834 | 57,46451 | 60,37203 | 62,28627 | 65,05438 | 66,815 |
| 157 | 39,0682 | 40,43498 | 42,66735 | 46,81655 | 52,15594 | 57,78323 | 60,68291 | 62,59517 | 65,36467 | 67,1286 |
| 158 | 39,41457 | 40,78928 | 43,0305 | 47,18153 | 52,49785 | 58,09527 | 60,98668 | 62,8967 | 65,66725 | 67,43429 |
| 159 | 39,75746 | 41,1399 | 43,38957 | 47,54166 | 52,8339 | 58,4006 | 61,28338 | 63,19095 | 65,96228 | 67,73228 |
| 160 | 40,09642 | 41,48639 | 43,74417 | 47,89662 | 53,16393 | 58,6992 | 61,57307 | 63,47803 | 66,24993 | 68,02279 |
| 161 | 40,43093 | 41,82828 | 44,09387 | 48,2461 | 53,48776 | 58,99105 | 61,8558 | 63,75803 | 66,53038 | 68,30603 |
| 162 | 40,76052 | 42,16511 | 44,43827 | 48,58979 | 53,80522 | 59,27612 | 62,13165 | 64,03107 | 66,8038 | 68,58225 |
| 163 | 41,08466 | 42,4964 | 44,77694 | 48,92736 | 54,11615 | 59,55442 | 62,40065 | 64,29727 | 67,07038 | 68,85166 |
| 164 | 41,40284 | 42,82166 | 45,10945 | 49,25851 | 54,42038 | 59,82591 | 62,66289 | 64,55673 | 67,33034 | 69,11453 |
| 165 | 41,71452 | 43,1404 | 45,43536 | 49,58291 | 54,71773 | 60,09059 | 62,91843 | 64,80959 | 67,58385 | 69,37111 |
| 166 | 42,01916 | 43,45212 | 45,75425 | 49,90024 | 55,00803 | 60,34845 | 63,16733 | 65,05596 | 67,83114 | 69,62165 |
| 167 | 42,31621 | 43,7563 | 46,06566 | 50,21019 | 55,29112 | 60,59947 | 63,40968 | 65,29598 | 68,07242 | 69,86643 |
| 168 | 42,60512 | 44,05243 | 46,36915 | 50,51242 | 55,56683 | 60,84364 | 63,64553 | 65,52977 | 68,3079 | 70,10573 |
| 169 | 42,88547 | 44,34014 | 46,6644 | 50,80675 | 55,83508 | 61,08103 | 63,87504 | 65,75753 | 68,53785 | 70,33986 |
| 170 | 43,15748 | 44,61966 | 46,95169 | 51,09346 | 56,09623 | 61,31201 | 64,09859 | 65,97966 | 68,7627 | 70,56926 |
| 171 | 43,4215 | 44,89137 | 47,2314 | 51,37299 | 56,35071 | 61,53704 | 64,31664 | 66,19661 | 68,98291 | 70,79441 |
| 172 | 43,67794 | 45,15567 | 47,50395 | 51,64578 | 56,59896 | 61,75655 | 64,52964 | 66,40885 | 69,19895 | 71,01577 |
| 173 | 43,92716 | 45,41295 | 47,76976 | 51,91225 | 56,84145 | 61,971 | 64,73803 | 66,61682 | 69,41126 | 71,2338 |
| 174 | 44,16959 | 45,66363 | 48,02925 | 52,17285 | 57,0786 | 62,18083 | 64,94225 | 66,82095 | 69,62028 | 71,44895 |
| 175 | 44,40561 | 45,90811 | 48,28285 | 52,42801 | 57,31086 | 62,38646 | 65,14274 | 67,02168 | 69,82646 | 71,66164 |
| 176 | 44,63564 | 46,14684 | 48,53099 | 52,67819 | 57,53868 | 62,58835 | 65,33993 | 67,21945 | 70,03022 | 71,87233 |
| 177 | 44,86011 | 46,38023 | 48,77411 | 52,92384 | 57,7625 | 62,78691 | 65,53424 | 67,41467 | 70,23199 | 72,08143 |
| 178 | 45,07944 | 46,60871 | 49,01265 | 53,1654 | 57,98277 | 62,98259 | 65,7261 | 67,60777 | 70,4322 | 72,28937 |
| 179 | 45,29406 | 46,83274 | 49,24707 | 53,40332 | 58,19992 | 63,1758 | 65,91593 | 67,79917 | 70,63125 | 72,49656 |
| 180 | 45,50442 | 47,05275 | 49,47783 | 53,63807 | 58,41442 | 63,36698 | 66,10415 | 67,98928 | 70,82955 | 72,70342 |
| 181 | 45,71083 | 47,26907 | 49,70523 | 53,86996 | 58,62655 | 63,5564 | 66,29103 | 68,17836 | 71,02736 | 72,91019 |
| 182 | 45,91312 | 47,48151 | 49,92909 | 54,09878 | 58,83606 | 63,74377 | 66,47625 | 68,36608 | 71,22434 | 73,11651 |
| 183 | 46,11097 | 47,68976 | 50,14908 | 54,32416 | 59,04257 | 63,92865 | 66,65934 | 68,55197 | 71,41998 | 73,32188 |
| 184 | 46,30408 | 47,8935 | 50,36487 | 54,54576 | 59,24567 | 64,11061 | 66,83986 | 68,73554 | 71,61378 | 73,52578 |
| 185 | 46,49214 | 48,09242 | 50,57613 | 54,7632 | 59,44497 | 64,28921 | 67,01733 | 68,91633 | 71,80525 | 73,72769 |
| 186 | 46,67485 | 48,28621 | 50,78254 | 54,97614 | 59,64008 | 64,46401 | 67,19131 | 69,09386 | 71,99388 | 73,92711 |
| 187 | 46,85192 | 48,47456 | 50,98376 | 55,18422 | 59,83061 | 64,63458 | 67,36133 | 69,26765 | 72,17918 | 74,12352 |
| 188 | 47,02303 | 48,65714 | 51,17946 | 55,38707 | 60,01615 | 64,80049 | 67,52693 | 69,43723 | 72,36065 | 74,3164 |
| 189 | 47,18789 | 48,83367 | 51,36933 | 55,58434 | 60,19632 | 64,96131 | 67,68766 | 69,60212 | 72,53778 | 74,50525 |
| 190 | 47,3462 | 49,00382 | 51,55302 | 55,77567 | 60,37073 | 65,1166 | 67,84306 | 69,76185 | 72,71009 | 74,68953 |
| 191 | 47,49768 | 49,16728 | 51,73022 | 55,9607 | 60,53896 | 65,26592 | 67,99267 | 69,91595 | 72,87706 | 74,86874 |
| 192 | 47,64201 | 49,32376 | 51,9006 | 56,13906 | 60,70064 | 65,40885 | 68,13604 | 70,06394 | 73,03819 | 75,04234 |
| 193 | 47,77906 | 49,4731 | 52,06398 | 56,31055 | 60,85554 | 65,54513 | 68,27287 | 70,20551 | 73,19315 | 75,21001 |
| 194 | 47,90929 | 49,61574 | 52,22083 | 56,47563 | 61,00406 | 65,67512 | 68,40354 | 70,34102 | 73,34231 | 75,37208 |
| 195 | 48,03329 | 49,75232 | 52,37176 | 56,63491 | 61,14678 | 65,79938 | 68,52857 | 70,47101 | 73,48617 | 75,52909 |
| 196 | 48,15168 | 49,88344 | 52,51739 | 56,78899 | 61,2843 | 65,91844 | 68,64848 | 70,59598 | 73,62526 | 75,68154 |
| 197 | 48,26506 | 50,00972 | 52,65836 | 56,9385 | 61,41717 | 66,03284 | 68,76381 | 70,71646 | 73,76008 | 75,82997 |
| 198 | 48,37406 | 50,13178 | 52,79528 | 57,08404 | 61,546 | 66,14313 | 68,87508 | 70,83295 | 73,89116 | 75,97488 |
| 199 | 48,47929 | 50,25026 | 52,92879 | 57,22625 | 61,67135 | 66,24982 | 68,98279 | 70,94598 | 74,019 | 76,11678 |
| 200 | 48,58137 | 50,36578 | 53,05952 | 57,36573 | 61,7938 | 66,35346 | 69,08747 | 71,05604 | 74,14409 | 76,25618 |
| 201 | 48,68094 | 50,47898 | 53,18812 | 57,50312 | 61,91393 | 66,45458 | 69,18963 | 71,16364 | 74,26694 | 76,39357 |
| 202 | 48,77861 | 50,5905 | 53,31522 | 57,63904 | 62,03233 | 66,5537 | 69,28977 | 71,26929 | 74,38805 | 76,52946 |
| 203 | 48,87502 | 50,70097 | 53,44147 | 57,77411 | 62,14957 | 66,65135 | 69,38841 | 71,37347 | 74,5079 | 76,66435 |

**Supplementary Table 5.** **Centile reference values for female weight**

| Months | 3th | 5th | 10th | 25th | 50th | 75th | 85th | 90th | 95th | 97th |
| --- | --- | --- | --- | --- | --- | --- | --- | --- | --- | --- |
| 72 | 18,09078 | 18,41479 | 18,99383 | 20,28149 | 22,42863 | 25,14694 | 26,6212 | 27,60369 | 29,04224 | 29,97167 |
| 73 | 18,27862 | 18,61077 | 19,20392 | 20,52098 | 22,71195 | 25,4786 | 26,97633 | 27,97352 | 29,43215 | 30,37356 |
| 74 | 18,46518 | 18,80564 | 19,4132 | 20,7602 | 22,99554 | 25,81076 | 27,33197 | 28,3438 | 29,82237 | 30,77562 |
| 75 | 18,65057 | 18,99953 | 19,62183 | 20,99934 | 23,27965 | 26,14376 | 27,68845 | 28,71489 | 30,21325 | 31,17824 |
| 76 | 18,83487 | 19,19255 | 19,82994 | 21,23859 | 23,56456 | 26,47791 | 28,04612 | 29,08713 | 30,60519 | 31,58179 |
| 77 | 19,01818 | 19,38482 | 20,03767 | 21,47816 | 23,85051 | 26,81353 | 28,40531 | 29,46088 | 30,99853 | 31,98665 |
| 78 | 19,20057 | 19,57642 | 20,24515 | 21,71822 | 24,13778 | 27,15095 | 28,76638 | 29,83649 | 31,39365 | 32,39318 |
| 79 | 19,38213 | 19,76746 | 20,45251 | 21,95898 | 24,42662 | 27,49048 | 29,12966 | 30,21432 | 31,79092 | 32,80176 |
| 80 | 19,56289 | 19,958 | 20,65986 | 22,20061 | 24,71731 | 27,83245 | 29,49549 | 30,59472 | 32,19068 | 33,21275 |
| 81 | 19,7429 | 20,14811 | 20,86732 | 22,44329 | 25,01009 | 28,17719 | 29,86423 | 30,97804 | 32,59332 | 33,62653 |
| 82 | 19,9222 | 20,33785 | 21,07496 | 22,6872 | 25,30525 | 28,52501 | 30,2362 | 31,36463 | 32,99918 | 34,04344 |
| 83 | 20,10079 | 20,52727 | 21,2829 | 22,93251 | 25,60303 | 28,87624 | 30,61176 | 31,75485 | 33,40862 | 34,46385 |
| 84 | 20,27868 | 20,71638 | 21,4912 | 23,17939 | 25,9037 | 29,2312 | 30,99124 | 32,14903 | 33,82199 | 34,88811 |
| 85 | 20,45582 | 20,90519 | 21,6999 | 23,42795 | 26,20747 | 29,59015 | 31,37491 | 32,54745 | 34,23956 | 35,31648 |
| 86 | 20,63205 | 21,09353 | 21,90888 | 23,67813 | 26,51427 | 29,95303 | 31,7627 | 32,95004 | 34,66125 | 35,74889 |
| 87 | 20,80717 | 21,28125 | 22,118 | 23,92978 | 26,824 | 30,31969 | 32,15446 | 33,35663 | 35,08689 | 36,18518 |
| 88 | 20,98101 | 21,46816 | 22,32709 | 24,18281 | 27,13653 | 30,69001 | 32,55004 | 33,76707 | 35,51634 | 36,62517 |
| 89 | 21,15336 | 21,65409 | 22,53602 | 24,43707 | 27,45176 | 31,06384 | 32,9493 | 34,18121 | 35,94944 | 37,06873 |
| 90 | 21,32403 | 21,83886 | 22,74461 | 24,69246 | 27,76957 | 31,44106 | 33,35208 | 34,59891 | 36,38603 | 37,5157 |
| 91 | 21,49283 | 22,0223 | 22,95274 | 24,94885 | 28,08983 | 31,82152 | 33,75825 | 35,02 | 36,82596 | 37,96593 |
| 92 | 21,65956 | 22,20423 | 23,16024 | 25,20612 | 28,41245 | 32,20509 | 34,16766 | 35,44435 | 37,26908 | 38,41928 |
| 93 | 21,82403 | 22,38447 | 23,36697 | 25,46416 | 28,73729 | 32,59164 | 34,58016 | 35,8718 | 37,71525 | 38,8756 |
| 94 | 21,98604 | 22,56286 | 23,57279 | 25,72285 | 29,06425 | 32,98102 | 34,99561 | 36,30221 | 38,16432 | 39,33476 |
| 95 | 22,14541 | 22,73921 | 23,77753 | 25,98207 | 29,39321 | 33,3731 | 35,41386 | 36,73543 | 38,61615 | 39,79662 |
| 96 | 22,30193 | 22,91336 | 23,98107 | 26,24171 | 29,72405 | 33,76774 | 35,83478 | 37,17132 | 39,07061 | 40,26105 |
| 97 | 22,45547 | 23,08519 | 24,18331 | 26,50171 | 30,05671 | 34,16486 | 36,25827 | 37,6098 | 39,52762 | 40,72799 |
| 98 | 22,60614 | 23,25481 | 24,38439 | 26,76221 | 30,39131 | 34,56455 | 36,68443 | 38,05096 | 39,98729 | 41,19757 |
| 99 | 22,75411 | 23,42243 | 24,58451 | 27,02342 | 30,72801 | 34,96695 | 37,11341 | 38,49497 | 40,44982 | 41,67 |
| 100 | 22,89957 | 23,58824 | 24,78389 | 27,28554 | 31,06699 | 35,37222 | 37,54536 | 38,94199 | 40,9154 | 42,14551 |
| 101 | 23,04274 | 23,75245 | 24,98274 | 27,54878 | 31,40841 | 35,78048 | 37,98043 | 39,39218 | 41,38423 | 42,62431 |
| 102 | 23,18384 | 23,91532 | 25,18131 | 27,81335 | 31,75244 | 36,1919 | 38,41878 | 39,84574 | 41,85652 | 43,10666 |
| 103 | 23,32314 | 24,07709 | 25,37985 | 28,07949 | 32,09925 | 36,60662 | 38,86058 | 40,30283 | 42,33249 | 43,59278 |
| 104 | 23,4609 | 24,23804 | 25,57863 | 28,34742 | 32,449 | 37,02478 | 39,306 | 40,76365 | 42,81237 | 44,08295 |
| 105 | 23,59746 | 24,39848 | 25,77794 | 28,61738 | 32,80187 | 37,44655 | 39,7552 | 41,22838 | 43,29639 | 44,57743 |
| 106 | 23,73314 | 24,55875 | 25,97808 | 28,88961 | 33,15803 | 37,87207 | 40,20836 | 41,69723 | 43,7848 | 45,0765 |
| 107 | 23,86833 | 24,7192 | 26,17939 | 29,16437 | 33,51764 | 38,30149 | 40,66566 | 42,1704 | 44,27784 | 45,58045 |
| 108 | 24,00344 | 24,88023 | 26,3822 | 29,4419 | 33,88086 | 38,73497 | 41,12728 | 42,6481 | 44,77577 | 46,08956 |
| 109 | 24,1389 | 25,04222 | 26,58685 | 29,72244 | 34,24782 | 39,17259 | 41,5933 | 43,13043 | 45,27875 | 46,60403 |
| 110 | 24,27502 | 25,20546 | 26,79354 | 30,00602 | 34,61835 | 39,61407 | 42,06343 | 43,61709 | 45,78646 | 47,12353 |
| 111 | 24,41216 | 25,37022 | 27,00246 | 30,29264 | 34,99226 | 40,05907 | 42,53729 | 44,10768 | 46,29847 | 47,64763 |
| 112 | 24,55071 | 25,53683 | 27,21381 | 30,58231 | 35,36933 | 40,50723 | 43,01447 | 44,60177 | 46,81436 | 48,1759 |
| 113 | 24,69111 | 25,70568 | 27,42786 | 30,87507 | 35,74935 | 40,9582 | 43,49458 | 45,09897 | 47,33371 | 48,70791 |
| 114 | 24,83388 | 25,87718 | 27,64487 | 31,17093 | 36,13211 | 41,41162 | 43,97724 | 45,59885 | 47,85608 | 49,24323 |
| 115 | 24,97958 | 26,0518 | 27,86516 | 31,46997 | 36,51741 | 41,86713 | 44,46203 | 46,10101 | 48,38106 | 49,78145 |
| 116 | 25,12886 | 26,23006 | 28,08909 | 31,77223 | 36,90503 | 42,32437 | 44,94855 | 46,60503 | 48,90822 | 50,32214 |
| 117 | 25,2824 | 26,41253 | 28,31702 | 32,07779 | 37,29477 | 42,78297 | 45,43642 | 47,11049 | 49,43713 | 50,86486 |
| 118 | 25,44094 | 26,59982 | 28,54937 | 32,38673 | 37,68642 | 43,24256 | 45,9252 | 47,61696 | 49,96736 | 51,40919 |
| 119 | 25,60529 | 26,79258 | 28,78655 | 32,69915 | 38,07977 | 43,70276 | 46,41449 | 48,12403 | 50,49848 | 51,95469 |
| 120 | 25,77629 | 26,99151 | 29,02905 | 33,01515 | 38,4746 | 44,16321 | 46,90387 | 48,63124 | 51,03003 | 52,50091 |
| 121 | 25,95474 | 27,19723 | 29,27725 | 33,33478 | 38,87069 | 44,62349 | 47,39291 | 49,13817 | 51,56158 | 53,04742 |
| 122 | 26,14102 | 27,41001 | 29,53123 | 33,65785 | 39,26764 | 45,08316 | 47,88114 | 49,64434 | 52,09268 | 53,59378 |
| 123 | 26,33543 | 27,63002 | 29,79098 | 33,98413 | 39,66505 | 45,54174 | 48,3681 | 50,14932 | 52,62289 | 54,13957 |
| 124 | 26,53824 | 27,8574 | 30,05648 | 34,31335 | 40,0625 | 45,99877 | 48,85332 | 50,65261 | 53,15176 | 54,68436 |
| 125 | 26,74971 | 28,09229 | 30,3277 | 34,64527 | 40,45958 | 46,45378 | 49,33633 | 51,15377 | 53,67883 | 55,2277 |
| 126 | 26,97006 | 28,33481 | 30,60461 | 34,97962 | 40,85588 | 46,9063 | 49,81663 | 51,65231 | 54,20366 | 55,76916 |
| 127 | 27,19951 | 28,58505 | 30,88713 | 35,31615 | 41,25098 | 47,35584 | 50,29376 | 52,14776 | 54,72576 | 56,30827 |
| 128 | 27,43821 | 28,84308 | 31,1752 | 35,65457 | 41,64448 | 47,80194 | 50,76723 | 52,63962 | 55,24467 | 56,84458 |
| 129 | 27,68629 | 29,10892 | 31,4687 | 35,99461 | 42,03597 | 48,24411 | 51,23655 | 53,12742 | 55,75992 | 57,37763 |
| 130 | 27,94383 | 29,38256 | 31,76751 | 36,33599 | 42,42502 | 48,68188 | 51,70123 | 53,61067 | 56,27102 | 57,90695 |
| 131 | 28,21086 | 29,66397 | 32,07148 | 36,6784 | 42,81122 | 49,11476 | 52,16077 | 54,08886 | 56,77748 | 58,43206 |
| 132 | 28,48733 | 29,95304 | 32,38044 | 37,02155 | 43,19418 | 49,54229 | 52,61468 | 54,56149 | 57,27882 | 58,95248 |
| 133 | 28,77322 | 30,24968 | 32,6942 | 37,36514 | 43,57348 | 49,96398 | 53,06247 | 55,02809 | 57,77454 | 59,46775 |
| 134 | 29,06858 | 30,5539 | 33,0127 | 37,70899 | 43,94882 | 50,37944 | 53,50372 | 55,48821 | 58,26424 | 59,97746 |
| 135 | 29,37344 | 30,86569 | 33,33587 | 38,0529 | 44,3199 | 50,78829 | 53,93802 | 55,94144 | 58,74749 | 60,48121 |
| 136 | 29,68778 | 31,185 | 33,66361 | 38,3967 | 44,68642 | 51,19011 | 54,36493 | 56,38734 | 59,22388 | 60,9786 |
| 137 | 30,01152 | 31,51174 | 33,9958 | 38,74019 | 45,04808 | 51,58454 | 54,78404 | 56,82549 | 59,69296 | 61,4692 |
| 138 | 30,34451 | 31,84576 | 34,33227 | 39,08317 | 45,40459 | 51,97115 | 55,19491 | 57,25542 | 60,15429 | 61,95258 |
| 139 | 30,68654 | 32,18685 | 34,67282 | 39,42541 | 45,75566 | 52,34956 | 55,59709 | 57,6767 | 60,60742 | 62,42829 |
| 140 | 31,03733 | 32,53476 | 35,01725 | 39,76668 | 46,10098 | 52,71935 | 55,99015 | 58,08883 | 61,05187 | 62,89588 |
| 141 | 31,39654 | 32,88921 | 35,36528 | 40,10676 | 46,44027 | 53,08012 | 56,37361 | 58,49136 | 61,48715 | 63,35488 |
| 142 | 31,76378 | 33,24984 | 35,71663 | 40,44538 | 46,77321 | 53,43146 | 56,74702 | 58,88378 | 61,91276 | 63,80477 |
| 143 | 32,13861 | 33,61626 | 36,07097 | 40,7823 | 47,09952 | 53,77295 | 57,10989 | 59,26559 | 62,32817 | 64,24506 |
| 144 | 32,5205 | 33,98804 | 36,42798 | 41,11723 | 47,4189 | 54,10417 | 57,46174 | 59,63627 | 62,73285 | 64,67519 |
| 145 | 32,90884 | 34,36465 | 36,78722 | 41,44994 | 47,73116 | 54,42484 | 57,80223 | 59,99544 | 63,12637 | 65,09476 |
| 146 | 33,30257 | 34,74525 | 37,14821 | 41,78034 | 48,03654 | 54,73527 | 58,13162 | 60,34333 | 63,50892 | 65,50392 |
| 147 | 33,70061 | 35,12898 | 37,51038 | 42,10839 | 48,33537 | 55,0359 | 58,45033 | 60,68033 | 63,88083 | 65,90297 |
| 148 | 34,1019 | 35,515 | 37,87324 | 42,43405 | 48,62799 | 55,32718 | 58,7588 | 61,00684 | 64,24245 | 66,29221 |
| 149 | 34,50544 | 35,90252 | 38,23628 | 42,75728 | 48,91474 | 55,60959 | 59,05747 | 61,32325 | 64,5941 | 66,67195 |
| 150 | 34,9103 | 36,29079 | 38,59903 | 43,07806 | 49,19598 | 55,88359 | 59,34677 | 61,62999 | 64,93613 | 67,04248 |
| 151 | 35,31562 | 36,67911 | 38,96106 | 43,39635 | 49,47202 | 56,14966 | 59,62718 | 61,92747 | 65,26892 | 67,40413 |
| 152 | 35,72059 | 37,06683 | 39,32195 | 43,71213 | 49,74322 | 56,40829 | 59,89917 | 62,21615 | 65,59284 | 67,75723 |
| 153 | 36,12449 | 37,45337 | 39,68134 | 44,0254 | 50,00992 | 56,65998 | 60,16323 | 62,49649 | 65,90827 | 68,10213 |
| 154 | 36,52665 | 37,83818 | 40,03888 | 44,33616 | 50,27246 | 56,90524 | 60,41986 | 62,76896 | 66,21565 | 68,43921 |
| 155 | 36,92646 | 38,22077 | 40,39427 | 44,64441 | 50,53117 | 57,14457 | 60,66961 | 63,03407 | 66,51542 | 68,76885 |
| 156 | 37,32341 | 38,60071 | 40,74722 | 44,95018 | 50,78639 | 57,37852 | 60,913 | 63,29235 | 66,80805 | 69,09148 |
| 157 | 37,71693 | 38,97756 | 41,09745 | 45,25345 | 51,03846 | 57,60762 | 61,1506 | 63,54436 | 67,09405 | 69,40758 |
| 158 | 38,10632 | 39,35067 | 41,4445 | 45,55408 | 51,2876 | 57,83239 | 61,38303 | 63,79071 | 67,37402 | 69,7177 |
| 159 | 38,49084 | 39,71943 | 41,78788 | 45,85191 | 51,53405 | 58,05337 | 61,61089 | 64,03206 | 67,64861 | 70,0225 |
| 160 | 38,86982 | 40,08323 | 42,12716 | 46,14677 | 51,77801 | 58,27112 | 61,83486 | 64,26912 | 67,91857 | 70,32268 |
| 161 | 39,24265 | 40,44154 | 42,46192 | 46,43852 | 52,01972 | 58,4862 | 62,05565 | 64,50264 | 68,18469 | 70,61906 |
| 162 | 39,60873 | 40,79383 | 42,79176 | 46,72702 | 52,25939 | 58,69922 | 62,27398 | 64,73344 | 68,44784 | 70,91254 |
| 163 | 39,96752 | 41,13963 | 43,1163 | 47,01212 | 52,49725 | 58,91077 | 62,49063 | 64,96238 | 68,70898 | 71,20409 |
| 164 | 40,31851 | 41,47847 | 43,43519 | 47,29371 | 52,73352 | 59,12147 | 62,7064 | 65,19036 | 68,96915 | 71,49481 |
| 165 | 40,6612 | 41,8099 | 43,74809 | 47,57165 | 52,96842 | 59,33196 | 62,92214 | 65,41836 | 69,22944 | 71,78587 |
| 166 | 40,99512 | 42,13352 | 44,05466 | 47,84582 | 53,20217 | 59,54289 | 63,13871 | 65,64737 | 69,49104 | 72,07854 |
| 167 | 41,3198 | 42,4489 | 44,35459 | 48,11611 | 53,43499 | 59,75492 | 63,35702 | 65,87845 | 69,7552 | 72,37421 |
| 168 | 41,63478 | 42,75562 | 44,64754 | 48,3824 | 53,6671 | 59,96872 | 63,57799 | 66,11269 | 70,02325 | 72,67432 |
| 169 | 41,93966 | 43,05333 | 44,93322 | 48,64452 | 53,89859 | 60,18474 | 63,80227 | 66,35088 | 70,29617 | 72,97996 |
| 170 | 42,23426 | 43,34179 | 45,21135 | 48,90212 | 54,12901 | 60,40246 | 64,02934 | 66,59248 | 70,57334 | 73,29046 |
| 171 | 42,51838 | 43,62079 | 45,48162 | 49,15476 | 54,35777 | 60,62115 | 64,25835 | 66,83655 | 70,85371 | 73,60464 |
| 172 | 42,79186 | 43,8901 | 45,74375 | 49,40202 | 54,58428 | 60,84003 | 64,48844 | 67,08216 | 71,13617 | 73,92126 |
| 173 | 43,05446 | 44,14945 | 45,99742 | 49,64347 | 54,80796 | 61,05835 | 64,71874 | 67,32834 | 71,41956 | 74,23901 |
| 174 | 43,30596 | 44,39859 | 46,24231 | 49,87868 | 55,0282 | 61,27533 | 64,94834 | 67,57409 | 71,70269 | 74,5565 |
| 175 | 43,54609 | 44,63723 | 46,47807 | 50,1072 | 55,24443 | 61,49019 | 65,17635 | 67,81838 | 71,9843 | 74,87228 |
| 176 | 43,77459 | 44,86507 | 46,70438 | 50,32861 | 55,45606 | 61,70216 | 65,40182 | 68,06016 | 72,26308 | 75,18483 |
| 177 | 43,99116 | 45,0818 | 46,92087 | 50,54246 | 55,6625 | 61,91042 | 65,62382 | 68,29835 | 72,53768 | 75,49253 |
| 178 | 44,1955 | 45,28709 | 47,12719 | 50,7483 | 55,86315 | 62,11419 | 65,84137 | 68,53183 | 72,80669 | 75,79373 |
| 179 | 44,3873 | 45,48061 | 47,32297 | 50,9457 | 56,05744 | 62,31264 | 66,0535 | 68,75946 | 73,06865 | 76,08669 |
| 180 | 44,5662 | 45,66201 | 47,50783 | 51,13419 | 56,24477 | 62,50497 | 66,2592 | 68,98009 | 73,32208 | 76,36959 |
| 181 | 44,73202 | 45,83107 | 47,68153 | 51,31348 | 56,42471 | 62,69055 | 66,4577 | 69,19281 | 73,56578 | 76,64104 |
| 182 | 44,88517 | 45,98818 | 47,84439 | 51,48378 | 56,59738 | 62,86949 | 66,64916 | 69,39781 | 73,80003 | 76,90131 |
| 183 | 45,02618 | 46,13383 | 47,99687 | 51,64547 | 56,76309 | 63,04212 | 66,83398 | 69,59557 | 74,02544 | 77,15115 |
| 184 | 45,15559 | 46,26853 | 48,13941 | 51,79889 | 56,92211 | 63,20875 | 67,01253 | 69,78653 | 74,24261 | 77,39129 |
| 185 | 45,27392 | 46,39277 | 48,27245 | 51,94441 | 57,07474 | 63,36967 | 67,18518 | 69,97114 | 74,45214 | 77,62243 |
| 186 | 45,3817 | 46,50704 | 48,39644 | 52,0824 | 57,22126 | 63,52519 | 67,35229 | 70,14984 | 74,65461 | 77,84531 |
| 187 | 45,47945 | 46,61183 | 48,51182 | 52,2132 | 57,36196 | 63,6756 | 67,51423 | 70,32305 | 74,8506 | 78,06064 |
| 188 | 45,56768 | 46,70764 | 48,61902 | 52,33718 | 57,49713 | 63,8212 | 67,67134 | 70,49119 | 75,0407 | 78,26913 |
| 189 | 45,64691 | 46,79493 | 48,7185 | 52,45471 | 57,62704 | 63,96227 | 67,82398 | 70,65467 | 75,22546 | 78,47151 |
| 190 | 45,71765 | 46,87422 | 48,81069 | 52,56613 | 57,752 | 64,0991 | 67,97247 | 70,81391 | 75,40546 | 78,66846 |
| 191 | 45,78043 | 46,94597 | 48,89603 | 52,67182 | 57,87229 | 64,23197 | 68,11717 | 70,96932 | 75,58125 | 78,86072 |
| 192 | 45,83578 | 47,0107 | 48,97497 | 52,77214 | 57,9882 | 64,36118 | 68,2584 | 71,12128 | 75,75341 | 79,04899 |
| 193 | 45,88422 | 47,0689 | 49,04797 | 52,86746 | 58,10002 | 64,48699 | 68,3965 | 71,27019 | 75,92242 | 79,2339 |
| 194 | 45,92626 | 47,12106 | 49,11547 | 52,95819 | 58,20808 | 64,60968 | 68,5317 | 71,41628 | 76,08857 | 79,41575 |
| 195 | 45,96241 | 47,16767 | 49,17797 | 53,04474 | 58,31272 | 64,72951 | 68,66425 | 71,55978 | 76,25205 | 79,59475 |
| 196 | 45,99317 | 47,20923 | 49,23592 | 53,12754 | 58,4143 | 64,84675 | 68,79437 | 71,7009 | 76,41309 | 79,77113 |
| 197 | 46,01908 | 47,24625 | 49,28981 | 53,20701 | 58,51314 | 64,96166 | 68,9223 | 71,83988 | 76,5719 | 79,9451 |
| 198 | 46,04063 | 47,27923 | 49,34011 | 53,28356 | 58,6096 | 65,0745 | 69,04828 | 71,97691 | 76,72868 | 80,11689 |
| 199 | 46,05837 | 47,30867 | 49,3873 | 53,35763 | 58,704 | 65,18553 | 69,17253 | 72,11225 | 76,88368 | 80,28673 |
| 200 | 46,07282 | 47,33509 | 49,43186 | 53,42962 | 58,7967 | 65,29504 | 69,29531 | 72,2461 | 77,0371 | 80,45487 |
| 201 | 46,0845 | 47,35901 | 49,47428 | 53,49996 | 58,88803 | 65,40327 | 69,41684 | 72,3787 | 77,1892 | 80,62157 |
| 202 | 46,09397 | 47,38094 | 49,51505 | 53,56909 | 58,97834 | 65,51051 | 69,53738 | 72,51028 | 77,3402 | 80,78707 |
| 203 | 46,10177 | 47,4014 | 49,55464 | 53,63741 | 59,06797 | 65,61701 | 69,65717 | 72,64109 | 77,49037 | 80,95166 |

**Supplementary Table 6.** **Centile reference values for male BMI**

| Months | 3th | 5th | 10th | 25th | 50th | 75th | 85th | 90th | 95th | 97th |
| --- | --- | --- | --- | --- | --- | --- | --- | --- | --- | --- |
| 72 | 13,19626 | 13,49292 | 13,97488 | 14,86289 | 15,99314 | 17,17601 | 17,7845 | 18,18565 | 18,76654 | 19,13648 |
| 73 | 13,23893 | 13,52983 | 14,00584 | 14,89519 | 16,04859 | 17,25876 | 17,87406 | 18,27652 | 18,85508 | 19,22117 |
| 74 | 13,28118 | 13,56672 | 14,03722 | 14,92809 | 16,10401 | 17,34097 | 17,96321 | 18,36717 | 18,94392 | 19,30661 |
| 75 | 13,32301 | 13,60358 | 14,06895 | 14,96149 | 16,15935 | 17,42266 | 18,05195 | 18,45762 | 19,03299 | 19,39271 |
| 76 | 13,36443 | 13,64036 | 14,10096 | 14,99532 | 16,21457 | 17,50381 | 18,1403 | 18,54784 | 19,12226 | 19,47935 |
| 77 | 13,40545 | 13,67703 | 14,13317 | 15,02949 | 16,26964 | 17,58444 | 18,22824 | 18,63782 | 19,21165 | 19,56646 |
| 78 | 13,44608 | 13,71358 | 14,16555 | 15,06395 | 16,32452 | 17,66452 | 18,31574 | 18,72752 | 19,3011 | 19,65392 |
| 79 | 13,48632 | 13,74998 | 14,19804 | 15,09862 | 16,37916 | 17,74404 | 18,4028 | 18,81691 | 19,39055 | 19,74164 |
| 80 | 13,52617 | 13,78622 | 14,23059 | 15,13345 | 16,43354 | 17,82297 | 18,48937 | 18,90595 | 19,47991 | 19,82951 |
| 81 | 13,56563 | 13,82226 | 14,26316 | 15,16838 | 16,4876 | 17,9013 | 18,57542 | 18,99457 | 19,56912 | 19,91744 |
| 82 | 13,60471 | 13,85809 | 14,29571 | 15,20335 | 16,54132 | 17,97897 | 18,6609 | 19,08274 | 19,65809 | 20,00532 |
| 83 | 13,64339 | 13,8937 | 14,32822 | 15,23834 | 16,59465 | 18,05597 | 18,74577 | 19,1704 | 19,74674 | 20,09306 |
| 84 | 13,68168 | 13,92905 | 14,36063 | 15,27327 | 16,64756 | 18,13224 | 18,82999 | 19,25747 | 19,835 | 20,18054 |
| 85 | 13,71957 | 13,96415 | 14,39293 | 15,30813 | 16,70001 | 18,20777 | 18,9135 | 19,34392 | 19,92278 | 20,26769 |
| 86 | 13,75706 | 13,999 | 14,42512 | 15,34292 | 16,75202 | 18,28255 | 18,99632 | 19,42975 | 20,0101 | 20,35452 |
| 87 | 13,79418 | 14,03359 | 14,45721 | 15,37764 | 16,8036 | 18,3566 | 19,07845 | 19,51497 | 20,097 | 20,44106 |
| 88 | 13,83094 | 14,06796 | 14,48922 | 15,41233 | 16,85477 | 18,42994 | 19,15992 | 19,59962 | 20,18348 | 20,52734 |
| 89 | 13,86735 | 14,10211 | 14,52116 | 15,44698 | 16,90555 | 18,50259 | 19,24075 | 19,6837 | 20,26959 | 20,61338 |
| 90 | 13,90342 | 14,13605 | 14,55304 | 15,48162 | 16,95596 | 18,57456 | 19,32095 | 19,76725 | 20,35533 | 20,69922 |
| 91 | 13,93918 | 14,16981 | 14,58487 | 15,51626 | 17,00601 | 18,64588 | 19,40054 | 19,85027 | 20,44074 | 20,78487 |
| 92 | 13,97464 | 14,20339 | 14,61667 | 15,5509 | 17,05573 | 18,71657 | 19,47954 | 19,93279 | 20,52584 | 20,87037 |
| 93 | 14,00982 | 14,23681 | 14,64846 | 15,58556 | 17,10512 | 18,78665 | 19,55798 | 20,01483 | 20,61066 | 20,95573 |
| 94 | 14,04475 | 14,27009 | 14,68023 | 15,62025 | 17,15422 | 18,85614 | 19,63586 | 20,09641 | 20,6952 | 21,04099 |
| 95 | 14,07943 | 14,30325 | 14,71202 | 15,65498 | 17,20303 | 18,92506 | 19,71321 | 20,17755 | 20,7795 | 21,12616 |
| 96 | 14,1139 | 14,3363 | 14,74384 | 15,68977 | 17,25158 | 18,99344 | 19,79006 | 20,25826 | 20,86358 | 21,21127 |
| 97 | 14,14815 | 14,36925 | 14,77568 | 15,72462 | 17,29988 | 19,06129 | 19,86641 | 20,33858 | 20,94747 | 21,29635 |
| 98 | 14,18216 | 14,40207 | 14,80753 | 15,75951 | 17,34793 | 19,12865 | 19,94232 | 20,41855 | 21,03122 | 21,38148 |
| 99 | 14,21588 | 14,43472 | 14,83934 | 15,79441 | 17,39574 | 19,19555 | 20,01781 | 20,49822 | 21,11491 | 21,46671 |
| 100 | 14,24927 | 14,46714 | 14,87108 | 15,8293 | 17,4433 | 19,26202 | 20,09294 | 20,57764 | 21,19859 | 21,55214 |
| 101 | 14,28229 | 14,49931 | 14,90271 | 15,86414 | 17,49062 | 19,32808 | 20,16775 | 20,65686 | 21,28234 | 21,63784 |
| 102 | 14,31488 | 14,53118 | 14,93418 | 15,89892 | 17,53769 | 19,39377 | 20,24229 | 20,73595 | 21,36623 | 21,7239 |
| 103 | 14,34701 | 14,56271 | 14,96548 | 15,93361 | 17,58452 | 19,45913 | 20,31659 | 20,81495 | 21,45034 | 21,81041 |
| 104 | 14,37863 | 14,59385 | 14,99655 | 15,96818 | 17,63111 | 19,52418 | 20,3907 | 20,89392 | 21,53475 | 21,89746 |
| 105 | 14,4097 | 14,62457 | 15,02736 | 16,00261 | 17,67746 | 19,58896 | 20,46468 | 20,97293 | 21,61953 | 21,98514 |
| 106 | 14,44017 | 14,65482 | 15,05788 | 16,03688 | 17,72356 | 19,65349 | 20,53856 | 21,05203 | 21,70476 | 22,07355 |
| 107 | 14,47 | 14,68457 | 15,08808 | 16,07095 | 17,76943 | 19,71781 | 20,61239 | 21,13128 | 21,79054 | 22,16279 |
| 108 | 14,49914 | 14,71376 | 15,11791 | 16,10482 | 17,81505 | 19,78194 | 20,68623 | 21,21074 | 21,87695 | 22,25299 |
| 109 | 14,52757 | 14,74239 | 15,14736 | 16,13846 | 17,86044 | 19,84591 | 20,7601 | 21,29046 | 21,96405 | 22,34421 |
| 110 | 14,55535 | 14,77049 | 15,17648 | 16,17192 | 17,90559 | 19,90968 | 20,83394 | 21,37038 | 22,05178 | 22,43638 |
| 111 | 14,58256 | 14,79816 | 15,20532 | 16,20522 | 17,95051 | 19,9732 | 20,90769 | 21,4504 | 22,14003 | 22,52939 |
| 112 | 14,60928 | 14,82547 | 15,23397 | 16,23841 | 17,9952 | 20,03644 | 20,98128 | 21,53044 | 22,2287 | 22,62313 |
| 113 | 14,6356 | 14,85248 | 15,26248 | 16,27154 | 18,03967 | 20,09933 | 21,05462 | 21,6104 | 22,31768 | 22,71749 |
| 114 | 14,66158 | 14,87928 | 15,29091 | 16,30465 | 18,08392 | 20,16182 | 21,12762 | 21,69019 | 22,40685 | 22,81235 |
| 115 | 14,68731 | 14,90592 | 15,31933 | 16,33779 | 18,12796 | 20,22386 | 21,20022 | 21,76972 | 22,49611 | 22,90759 |
| 116 | 14,71285 | 14,93249 | 15,3478 | 16,371 | 18,17178 | 20,28538 | 21,27231 | 21,84886 | 22,58535 | 23,0031 |
| 117 | 14,73828 | 14,95905 | 15,37639 | 16,40433 | 18,21539 | 20,34632 | 21,3438 | 21,92753 | 22,67443 | 23,09875 |
| 118 | 14,76366 | 14,98568 | 15,40517 | 16,43784 | 18,25881 | 20,40662 | 21,41459 | 22,0056 | 22,76324 | 23,19442 |
| 119 | 14,78907 | 15,01243 | 15,4342 | 16,47157 | 18,30202 | 20,46621 | 21,4846 | 22,08296 | 22,85166 | 23,28999 |
| 120 | 14,81457 | 15,03937 | 15,46354 | 16,50557 | 18,34503 | 20,52501 | 21,5537 | 22,15949 | 22,93955 | 23,38533 |
| 121 | 14,84022 | 15,06657 | 15,49325 | 16,5399 | 18,38786 | 20,58296 | 21,6218 | 22,23508 | 23,0268 | 23,48031 |
| 122 | 14,86607 | 15,09407 | 15,52338 | 16,57458 | 18,43049 | 20,64001 | 21,68884 | 22,30965 | 23,11329 | 23,57482 |
| 123 | 14,89217 | 15,12192 | 15,55395 | 16,60964 | 18,47294 | 20,69612 | 21,75474 | 22,38312 | 23,19893 | 23,66876 |
| 124 | 14,91857 | 15,15014 | 15,58501 | 16,6451 | 18,5152 | 20,75126 | 21,81945 | 22,4554 | 23,28363 | 23,762 |
| 125 | 14,94531 | 15,1788 | 15,61658 | 16,68099 | 18,55728 | 20,80539 | 21,88291 | 22,52642 | 23,36726 | 23,85442 |
| 126 | 14,97245 | 15,20793 | 15,6487 | 16,71732 | 18,59918 | 20,85846 | 21,94505 | 22,5961 | 23,44972 | 23,94591 |
| 127 | 15,00003 | 15,23757 | 15,6814 | 16,75412 | 18,64089 | 20,91044 | 22,00581 | 22,66436 | 23,5309 | 24,03631 |
| 128 | 15,0281 | 15,26776 | 15,71471 | 16,79142 | 18,68244 | 20,96129 | 22,06512 | 22,7311 | 23,61068 | 24,1255 |
| 129 | 15,05673 | 15,29855 | 15,74867 | 16,82923 | 18,72381 | 21,01098 | 22,12293 | 22,79625 | 23,68894 | 24,21333 |
| 130 | 15,08595 | 15,32999 | 15,78331 | 16,86756 | 18,76501 | 21,05948 | 22,17918 | 22,85972 | 23,76554 | 24,29962 |
| 131 | 15,11583 | 15,36213 | 15,81866 | 16,90645 | 18,80603 | 21,10676 | 22,23381 | 22,92143 | 23,84036 | 24,38423 |
| 132 | 15,14643 | 15,395 | 15,85475 | 16,94589 | 18,84689 | 21,1528 | 22,28678 | 22,9813 | 23,91324 | 24,46695 |
| 133 | 15,17779 | 15,42865 | 15,8916 | 16,98589 | 18,88759 | 21,19758 | 22,33803 | 23,03927 | 23,98409 | 24,54765 |
| 134 | 15,20991 | 15,46306 | 15,92919 | 17,02644 | 18,92812 | 21,24114 | 22,38761 | 23,09534 | 24,05289 | 24,6263 |
| 135 | 15,24274 | 15,49819 | 15,96748 | 17,06752 | 18,9685 | 21,28349 | 22,43555 | 23,14957 | 24,11968 | 24,70292 |
| 136 | 15,27628 | 15,53403 | 16,00646 | 17,10908 | 19,00871 | 21,32467 | 22,48189 | 23,20199 | 24,18448 | 24,77753 |
| 137 | 15,31049 | 15,57053 | 16,04607 | 17,15112 | 19,04876 | 21,36474 | 22,52668 | 23,25264 | 24,24733 | 24,85014 |
| 138 | 15,34535 | 15,60768 | 16,0863 | 17,1936 | 19,08867 | 21,40371 | 22,56997 | 23,30158 | 24,30825 | 24,92078 |
| 139 | 15,38083 | 15,64545 | 16,12711 | 17,23649 | 19,12841 | 21,44165 | 22,61181 | 23,34886 | 24,36728 | 24,98945 |
| 140 | 15,41693 | 15,68381 | 16,16846 | 17,27977 | 19,16801 | 21,4786 | 22,65227 | 23,39452 | 24,42444 | 25,05617 |
| 141 | 15,45361 | 15,72274 | 16,21033 | 17,32339 | 19,20746 | 21,51461 | 22,69141 | 23,43862 | 24,47979 | 25,12094 |
| 142 | 15,49087 | 15,76221 | 16,25267 | 17,36732 | 19,24675 | 21,54973 | 22,72928 | 23,48123 | 24,53333 | 25,18378 |
| 143 | 15,5287 | 15,8022 | 16,29546 | 17,41153 | 19,28591 | 21,58403 | 22,76596 | 23,5224 | 24,58512 | 25,2447 |
| 144 | 15,56706 | 15,84269 | 16,33866 | 17,45597 | 19,32492 | 21,61756 | 22,80152 | 23,56221 | 24,63519 | 25,30369 |
| 145 | 15,60596 | 15,88364 | 16,38224 | 17,50061 | 19,36379 | 21,65039 | 22,83603 | 23,60071 | 24,68358 | 25,36078 |
| 146 | 15,64536 | 15,92504 | 16,42615 | 17,54542 | 19,40252 | 21,68257 | 22,86958 | 23,638 | 24,73037 | 25,41604 |
| 147 | 15,6852 | 15,96682 | 16,47034 | 17,59035 | 19,44111 | 21,71417 | 22,90223 | 23,67415 | 24,77565 | 25,46955 |
| 148 | 15,72545 | 16,00894 | 16,51478 | 17,63538 | 19,47956 | 21,74523 | 22,93407 | 23,70926 | 24,81952 | 25,52142 |
| 149 | 15,76605 | 16,05136 | 16,55942 | 17,68047 | 19,51788 | 21,77581 | 22,96517 | 23,74341 | 24,86207 | 25,57173 |
| 150 | 15,80698 | 16,09403 | 16,60422 | 17,72558 | 19,55606 | 21,80597 | 22,9956 | 23,7767 | 24,90342 | 25,62061 |
| 151 | 15,84816 | 16,13691 | 16,64914 | 17,7707 | 19,59411 | 21,83575 | 23,02545 | 23,8092 | 24,94365 | 25,66815 |
| 152 | 15,88958 | 16,17996 | 16,69413 | 17,81577 | 19,63203 | 21,86521 | 23,0548 | 23,84102 | 24,98288 | 25,71447 |
| 153 | 15,93116 | 16,22312 | 16,73915 | 17,86077 | 19,66982 | 21,89441 | 23,08371 | 23,87225 | 25,02122 | 25,75969 |
| 154 | 15,97287 | 16,26635 | 16,78416 | 17,90568 | 19,70748 | 21,92339 | 23,11228 | 23,90298 | 25,05879 | 25,80394 |
| 155 | 16,01466 | 16,3096 | 16,82911 | 17,95045 | 19,74502 | 21,95222 | 23,14057 | 23,93331 | 25,0957 | 25,84734 |
| 156 | 16,05647 | 16,35282 | 16,87396 | 17,99506 | 19,78243 | 21,98093 | 23,16867 | 23,96333 | 25,13207 | 25,89003 |
| 157 | 16,09827 | 16,39598 | 16,91867 | 18,03948 | 19,81971 | 22,00958 | 23,19664 | 23,99313 | 25,16801 | 25,93213 |
| 158 | 16,14003 | 16,43904 | 16,96322 | 18,08369 | 19,85687 | 22,03819 | 23,22452 | 24,02274 | 25,20357 | 25,97369 |
| 159 | 16,18173 | 16,48201 | 17,0076 | 18,1277 | 19,89391 | 22,06677 | 23,25234 | 24,05221 | 25,2388 | 26,01477 |
| 160 | 16,22336 | 16,52485 | 17,0518 | 18,17148 | 19,93083 | 22,09535 | 23,28012 | 24,08157 | 25,27373 | 26,05541 |
| 161 | 16,26491 | 16,56756 | 17,09581 | 18,21503 | 19,96763 | 22,12394 | 23,3079 | 24,11085 | 25,30841 | 26,09566 |
| 162 | 16,30635 | 16,61011 | 17,1396 | 18,25834 | 20,00431 | 22,15256 | 23,33568 | 24,14008 | 25,34289 | 26,13558 |
| 163 | 16,34766 | 16,6525 | 17,18317 | 18,30141 | 20,04087 | 22,18122 | 23,36351 | 24,1693 | 25,37721 | 26,1752 |
| 164 | 16,38884 | 16,69471 | 17,22651 | 18,34422 | 20,07732 | 22,20994 | 23,3914 | 24,19853 | 25,41141 | 26,2146 |
| 165 | 16,42985 | 16,73672 | 17,26959 | 18,38677 | 20,11364 | 22,23873 | 23,41938 | 24,22781 | 25,44555 | 26,25383 |
| 166 | 16,47069 | 16,77851 | 17,31242 | 18,42905 | 20,14986 | 22,2676 | 23,44747 | 24,25718 | 25,47967 | 26,29295 |
| 167 | 16,51133 | 16,82007 | 17,35497 | 18,47106 | 20,18596 | 22,29657 | 23,47569 | 24,28666 | 25,51382 | 26,33202 |
| 168 | 16,55174 | 16,86138 | 17,39723 | 18,5128 | 20,22194 | 22,32564 | 23,50407 | 24,31629 | 25,54806 | 26,37111 |
| 169 | 16,59193 | 16,90243 | 17,4392 | 18,55425 | 20,25782 | 22,35483 | 23,53262 | 24,34609 | 25,58242 | 26,41027 |
| 170 | 16,63187 | 16,94321 | 17,48088 | 18,59542 | 20,29358 | 22,38413 | 23,56134 | 24,37606 | 25,6169 | 26,4495 |
| 171 | 16,6716 | 16,98374 | 17,52228 | 18,63632 | 20,32924 | 22,41355 | 23,59022 | 24,4062 | 25,65151 | 26,4888 |
| 172 | 16,7111 | 17,02403 | 17,56339 | 18,67696 | 20,36479 | 22,44307 | 23,61927 | 24,43649 | 25,68622 | 26,52815 |
| 173 | 16,75039 | 17,06407 | 17,60424 | 18,71733 | 20,40023 | 22,4727 | 23,64848 | 24,46694 | 25,72104 | 26,56755 |
| 174 | 16,78948 | 17,10389 | 17,64482 | 18,75744 | 20,43557 | 22,50243 | 23,67783 | 24,49754 | 25,75595 | 26,60699 |
| 175 | 16,82837 | 17,14347 | 17,68515 | 18,79731 | 20,4708 | 22,53225 | 23,70732 | 24,52827 | 25,79095 | 26,64646 |
| 176 | 16,86707 | 17,18284 | 17,72523 | 18,83693 | 20,50594 | 22,56217 | 23,73695 | 24,55913 | 25,82602 | 26,68595 |
| 177 | 16,90558 | 17,222 | 17,76506 | 18,87631 | 20,54097 | 22,59217 | 23,7667 | 24,59012 | 25,86116 | 26,72545 |
| 178 | 16,94392 | 17,26096 | 17,80467 | 18,91547 | 20,57591 | 22,62225 | 23,79657 | 24,62121 | 25,89636 | 26,76495 |
| 179 | 16,98208 | 17,29972 | 17,84404 | 18,9544 | 20,61075 | 22,65241 | 23,82655 | 24,65241 | 25,93162 | 26,80444 |
| 180 | 17,02008 | 17,33828 | 17,8832 | 18,99311 | 20,6455 | 22,68263 | 23,85664 | 24,6837 | 25,96691 | 26,84392 |
| 181 | 17,05792 | 17,37667 | 17,92215 | 19,03161 | 20,68015 | 22,71292 | 23,88681 | 24,71508 | 26,00224 | 26,88338 |
| 182 | 17,0956 | 17,41488 | 17,96088 | 19,06991 | 20,71471 | 22,74326 | 23,91707 | 24,74653 | 26,03758 | 26,92279 |
| 183 | 17,13314 | 17,45291 | 17,99942 | 19,108 | 20,74917 | 22,77365 | 23,94741 | 24,77804 | 26,07292 | 26,96214 |
| 184 | 17,17053 | 17,49077 | 18,03776 | 19,14589 | 20,78353 | 22,80409 | 23,97781 | 24,8096 | 26,10824 | 27,00141 |
| 185 | 17,20778 | 17,52847 | 18,0759 | 19,18357 | 20,8178 | 22,83456 | 24,00826 | 24,8412 | 26,14354 | 27,04057 |
| 186 | 17,2449 | 17,56601 | 18,11386 | 19,22106 | 20,85196 | 22,86506 | 24,03876 | 24,87282 | 26,17878 | 27,0796 |
| 187 | 17,28188 | 17,60339 | 18,15162 | 19,25835 | 20,88602 | 22,89558 | 24,06929 | 24,90445 | 26,21395 | 27,1185 |
| 188 | 17,31875 | 17,64063 | 18,18921 | 19,29544 | 20,91998 | 22,92612 | 24,09984 | 24,93608 | 26,24904 | 27,15722 |
| 189 | 17,35549 | 17,67772 | 18,22661 | 19,33235 | 20,95383 | 22,95666 | 24,13041 | 24,96769 | 26,28403 | 27,19576 |
| 190 | 17,39212 | 17,71467 | 18,26384 | 19,36906 | 20,98758 | 22,98721 | 24,16098 | 24,99928 | 26,31889 | 27,23408 |
| 191 | 17,42864 | 17,75148 | 18,30091 | 19,40559 | 21,02122 | 23,01775 | 24,19154 | 25,03082 | 26,35362 | 27,27218 |
| 192 | 17,46506 | 17,78816 | 18,3378 | 19,44193 | 21,05475 | 23,04827 | 24,22208 | 25,06231 | 26,3882 | 27,31002 |
| 193 | 17,50137 | 17,82471 | 18,37454 | 19,47809 | 21,08816 | 23,07878 | 24,25259 | 25,09373 | 26,4226 | 27,34759 |
| 194 | 17,5376 | 17,86116 | 18,41113 | 19,51409 | 21,12148 | 23,10926 | 24,28307 | 25,12509 | 26,45684 | 27,38491 |
| 195 | 17,57375 | 17,89749 | 18,44759 | 19,54994 | 21,15471 | 23,13972 | 24,31351 | 25,15638 | 26,49092 | 27,42198 |
| 196 | 17,60982 | 17,93374 | 18,48393 | 19,58565 | 21,18785 | 23,17016 | 24,34393 | 25,18761 | 26,52486 | 27,45882 |
| 197 | 17,64583 | 17,9699 | 18,52017 | 19,62125 | 21,22091 | 23,20058 | 24,37432 | 25,21878 | 26,55866 | 27,49546 |
| 198 | 17,68179 | 18,006 | 18,55631 | 19,65674 | 21,25392 | 23,23099 | 24,40467 | 25,2499 | 26,59233 | 27,5319 |
| 199 | 17,7177 | 18,04203 | 18,59238 | 19,69214 | 21,28686 | 23,26137 | 24,435 | 25,28095 | 26,62588 | 27,56816 |
| 200 | 17,75358 | 18,07802 | 18,62839 | 19,72748 | 21,31976 | 23,29173 | 24,46529 | 25,31195 | 26,65931 | 27,60427 |
| 201 | 17,78943 | 18,11397 | 18,66436 | 19,76275 | 21,35263 | 23,32207 | 24,49555 | 25,3429 | 26,69265 | 27,64023 |
| 202 | 17,82526 | 18,1499 | 18,70028 | 19,79799 | 21,38546 | 23,35239 | 24,52577 | 25,37379 | 26,72589 | 27,67606 |
| 203 | 17,86108 | 18,18582 | 18,7362 | 19,83321 | 21,41828 | 23,38269 | 24,55595 | 25,40462 | 26,75905 | 27,71178 |

**Supplementary Table 7.** **Centile reference values for female BMI**

| Months | 3th | 5th | 10th | 25th | 50th | 75th | 85th | 90th | 95th | 97th |
| --- | --- | --- | --- | --- | --- | --- | --- | --- | --- | --- |
| 72 | 13,17031 | 13,4681 | 13,96482 | 14,92783 | 16,23753 | 17,61943 | 18,30089 | 18,73699 | 19,35134 | 19,73304 |
| 73 | 13,198 | 13,49535 | 13,99214 | 14,95821 | 16,27771 | 17,67478 | 18,36478 | 18,80662 | 19,42939 | 19,81656 |
| 74 | 13,22575 | 13,52265 | 14,0195 | 14,98859 | 16,3179 | 17,73027 | 18,42892 | 18,87659 | 19,50795 | 19,90067 |
| 75 | 13,25348 | 13,54995 | 14,04687 | 15,01898 | 16,35812 | 17,78591 | 18,49333 | 18,9469 | 19,58698 | 19,98536 |
| 76 | 13,28115 | 13,57721 | 14,07421 | 15,04936 | 16,39837 | 17,84171 | 18,55799 | 19,01755 | 19,66646 | 20,07058 |
| 77 | 13,30868 | 13,60435 | 14,10148 | 15,07972 | 16,43867 | 17,89768 | 18,6229 | 19,0885 | 19,74636 | 20,15629 |
| 78 | 13,336 | 13,63135 | 14,12867 | 15,11007 | 16,47903 | 17,95382 | 18,68805 | 19,15976 | 19,82663 | 20,24243 |
| 79 | 13,36306 | 13,65813 | 14,15572 | 15,14039 | 16,51946 | 18,01013 | 18,75345 | 19,23128 | 19,90723 | 20,32893 |
| 80 | 13,38979 | 13,68465 | 14,1826 | 15,17068 | 16,55997 | 18,06663 | 18,81906 | 19,30306 | 19,98812 | 20,41575 |
| 81 | 13,41612 | 13,71086 | 14,20928 | 15,20093 | 16,60058 | 18,12331 | 18,88489 | 19,37506 | 20,06923 | 20,50278 |
| 82 | 13,44198 | 13,73668 | 14,23572 | 15,23112 | 16,64129 | 18,18018 | 18,95091 | 19,44724 | 20,1505 | 20,58995 |
| 83 | 13,4673 | 13,76207 | 14,26187 | 15,26125 | 16,68213 | 18,23724 | 19,0171 | 19,51958 | 20,23187 | 20,67717 |
| 84 | 13,49201 | 13,78697 | 14,28769 | 15,29131 | 16,72309 | 18,29448 | 19,08345 | 19,59202 | 20,31325 | 20,76432 |
| 85 | 13,51604 | 13,81131 | 14,31316 | 15,32129 | 16,7642 | 18,3519 | 19,14992 | 19,66452 | 20,39456 | 20,8513 |
| 86 | 13,53937 | 13,83508 | 14,33824 | 15,35118 | 16,80544 | 18,4095 | 19,21651 | 19,73707 | 20,47576 | 20,93805 |
| 87 | 13,56198 | 13,85827 | 14,36292 | 15,38096 | 16,84682 | 18,46727 | 19,28318 | 19,80961 | 20,5568 | 21,0245 |
| 88 | 13,58385 | 13,88084 | 14,38719 | 15,41064 | 16,88834 | 18,52519 | 19,34992 | 19,88212 | 20,63762 | 21,1106 |
| 89 | 13,60496 | 13,90278 | 14,41103 | 15,4402 | 16,92998 | 18,58326 | 19,4167 | 19,95458 | 20,71818 | 21,19628 |
| 90 | 13,62529 | 13,92407 | 14,43443 | 15,46964 | 16,97176 | 18,64145 | 19,48351 | 20,02694 | 20,79843 | 21,28148 |
| 91 | 13,6448 | 13,94469 | 14,45736 | 15,49894 | 17,01366 | 18,69977 | 19,55031 | 20,09918 | 20,87832 | 21,36613 |
| 92 | 13,66348 | 13,96462 | 14,47982 | 15,52809 | 17,05568 | 18,7582 | 19,61709 | 20,17127 | 20,95781 | 21,45017 |
| 93 | 13,68129 | 13,98382 | 14,50176 | 15,5571 | 17,09782 | 18,81673 | 19,68382 | 20,24316 | 21,03682 | 21,53354 |
| 94 | 13,6982 | 14,00227 | 14,52319 | 15,58593 | 17,14007 | 18,87534 | 19,75048 | 20,31483 | 21,11533 | 21,61618 |
| 95 | 13,71419 | 14,01994 | 14,54407 | 15,61459 | 17,18244 | 18,93403 | 19,81705 | 20,38624 | 21,19328 | 21,69803 |
| 96 | 13,72921 | 14,03681 | 14,56437 | 15,64306 | 17,22492 | 18,99278 | 19,8835 | 20,45736 | 21,27062 | 21,77901 |
| 97 | 13,74324 | 14,05285 | 14,5841 | 15,67135 | 17,26751 | 19,05158 | 19,94982 | 20,52817 | 21,34731 | 21,8591 |
| 98 | 13,75637 | 14,06812 | 14,6033 | 15,69946 | 17,3102 | 19,11042 | 20,01599 | 20,59866 | 21,42337 | 21,93833 |
| 99 | 13,76868 | 14,08272 | 14,62202 | 15,72743 | 17,35301 | 19,16931 | 20,08204 | 20,66887 | 21,49886 | 22,01676 |
| 100 | 13,78028 | 14,09673 | 14,64034 | 15,7553 | 17,39592 | 19,22824 | 20,14797 | 20,73881 | 21,57381 | 22,09446 |
| 101 | 13,79127 | 14,11023 | 14,65831 | 15,78309 | 17,43895 | 19,2872 | 20,21378 | 20,80851 | 21,6483 | 22,17151 |
| 102 | 13,80176 | 14,12332 | 14,67603 | 15,81083 | 17,48208 | 19,34621 | 20,27951 | 20,87801 | 21,72237 | 22,24799 |
| 103 | 13,81186 | 14,13611 | 14,69355 | 15,83857 | 17,52534 | 19,40525 | 20,34517 | 20,94733 | 21,7961 | 22,32402 |
| 104 | 13,8217 | 14,1487 | 14,71097 | 15,86634 | 17,5687 | 19,46435 | 20,41077 | 21,01653 | 21,86957 | 22,39969 |
| 105 | 13,83142 | 14,1612 | 14,72836 | 15,89418 | 17,61218 | 19,52348 | 20,47635 | 21,08564 | 21,94285 | 22,47511 |
| 106 | 13,84113 | 14,17372 | 14,74581 | 15,92213 | 17,65578 | 19,58268 | 20,54194 | 21,15472 | 22,01605 | 22,5504 |
| 107 | 13,851 | 14,1864 | 14,76341 | 15,95022 | 17,6995 | 19,64193 | 20,60755 | 21,22381 | 22,08925 | 22,62569 |
| 108 | 13,86117 | 14,19936 | 14,78127 | 15,97851 | 17,74334 | 19,70126 | 20,67323 | 21,29298 | 22,16255 | 22,70111 |
| 109 | 13,8718 | 14,21274 | 14,79948 | 16,00704 | 17,78729 | 19,76065 | 20,73901 | 21,36227 | 22,23604 | 22,77677 |
| 110 | 13,883 | 14,22664 | 14,81812 | 16,03585 | 17,83137 | 19,82011 | 20,80487 | 21,43168 | 22,30972 | 22,8527 |
| 111 | 13,89491 | 14,24117 | 14,83728 | 16,06498 | 17,87556 | 19,8796 | 20,87079 | 21,5012 | 22,38361 | 22,92891 |
| 112 | 13,90764 | 14,25644 | 14,85705 | 16,09448 | 17,91988 | 19,93912 | 20,93676 | 21,57081 | 22,45769 | 23,00539 |
| 113 | 13,92132 | 14,27257 | 14,87752 | 16,12438 | 17,96432 | 19,99863 | 21,00276 | 21,64049 | 22,53196 | 23,08217 |
| 114 | 13,93609 | 14,28967 | 14,89876 | 16,15475 | 18,00888 | 20,05813 | 21,06877 | 21,71025 | 22,60643 | 23,15924 |
| 115 | 13,95208 | 14,30785 | 14,92088 | 16,18561 | 18,05355 | 20,11758 | 21,13477 | 21,78006 | 22,68109 | 23,23663 |
| 116 | 13,96941 | 14,32723 | 14,94396 | 16,21703 | 18,09835 | 20,17698 | 21,20076 | 21,84993 | 22,75596 | 23,31436 |
| 117 | 13,98824 | 14,34794 | 14,96811 | 16,24904 | 18,14327 | 20,2363 | 21,26671 | 21,91983 | 22,83104 | 23,39243 |
| 118 | 14,00868 | 14,37008 | 14,9934 | 16,28169 | 18,18831 | 20,29553 | 21,33261 | 21,98976 | 22,90633 | 23,47087 |
| 119 | 14,03088 | 14,39379 | 15,01995 | 16,31504 | 18,23347 | 20,35465 | 21,39845 | 22,05973 | 22,98185 | 23,54971 |
| 120 | 14,05497 | 14,41918 | 15,04784 | 16,34912 | 18,27875 | 20,41363 | 21,46421 | 22,12971 | 23,05761 | 23,62896 |
| 121 | 14,08107 | 14,44635 | 15,07714 | 16,38399 | 18,32415 | 20,47246 | 21,52988 | 22,1997 | 23,13361 | 23,70865 |
| 122 | 14,10915 | 14,47529 | 15,10786 | 16,41963 | 18,36967 | 20,53114 | 21,59545 | 22,26971 | 23,20987 | 23,78879 |
| 123 | 14,13921 | 14,50599 | 15,13999 | 16,45606 | 18,41532 | 20,58966 | 21,66093 | 22,33973 | 23,28637 | 23,86937 |
| 124 | 14,17119 | 14,5384 | 15,17351 | 16,49326 | 18,46108 | 20,64803 | 21,72631 | 22,40975 | 23,36311 | 23,9504 |
| 125 | 14,20506 | 14,57251 | 15,20838 | 16,53123 | 18,50697 | 20,70624 | 21,79159 | 22,47978 | 23,4401 | 24,03186 |
| 126 | 14,24078 | 14,60827 | 15,2446 | 16,56995 | 18,55298 | 20,76429 | 21,85676 | 22,54981 | 23,51732 | 24,11376 |
| 127 | 14,27828 | 14,64564 | 15,28213 | 16,60943 | 18,59911 | 20,82218 | 21,92182 | 22,61982 | 23,59476 | 24,19609 |
| 128 | 14,31754 | 14,68458 | 15,32094 | 16,64964 | 18,64536 | 20,87989 | 21,98675 | 22,68981 | 23,67242 | 24,27882 |
| 129 | 14,35848 | 14,72505 | 15,36101 | 16,69059 | 18,69174 | 20,93744 | 22,05155 | 22,75976 | 23,75027 | 24,36195 |
| 130 | 14,40105 | 14,767 | 15,4023 | 16,73226 | 18,73824 | 20,9948 | 22,11621 | 22,82967 | 23,8283 | 24,44546 |
| 131 | 14,44519 | 14,81038 | 15,44479 | 16,77464 | 18,78486 | 21,05197 | 22,18071 | 22,89951 | 23,9065 | 24,52933 |
| 132 | 14,49085 | 14,85515 | 15,48842 | 16,81772 | 18,83161 | 21,10896 | 22,24504 | 22,96927 | 23,98483 | 24,61354 |
| 133 | 14,53797 | 14,90124 | 15,53318 | 16,86148 | 18,87849 | 21,16574 | 22,30919 | 23,03893 | 24,06329 | 24,69806 |
| 134 | 14,58648 | 14,94863 | 15,57903 | 16,90592 | 18,92548 | 21,22233 | 22,37315 | 23,10849 | 24,14186 | 24,78289 |
| 135 | 14,63636 | 14,99727 | 15,62595 | 16,95103 | 18,97261 | 21,2787 | 22,43692 | 23,17793 | 24,22052 | 24,86802 |
| 136 | 14,68753 | 15,04711 | 15,6739 | 16,99678 | 19,01986 | 21,33488 | 22,50048 | 23,24724 | 24,29928 | 24,95343 |
| 137 | 14,73996 | 15,09812 | 15,72285 | 17,04318 | 19,06723 | 21,39084 | 22,56384 | 23,31642 | 24,37812 | 25,03913 |
| 138 | 14,7936 | 15,15025 | 15,77277 | 17,0902 | 19,11473 | 21,44658 | 22,62697 | 23,38545 | 24,45701 | 25,12509 |
| 139 | 14,84839 | 15,20347 | 15,82363 | 17,13784 | 19,16236 | 21,50211 | 22,68987 | 23,45432 | 24,53596 | 25,21129 |
| 140 | 14,90429 | 15,25772 | 15,8754 | 17,18609 | 19,21011 | 21,55741 | 22,75253 | 23,52301 | 24,61493 | 25,29773 |
| 141 | 14,96124 | 15,31296 | 15,92804 | 17,23492 | 19,25799 | 21,61248 | 22,81494 | 23,59151 | 24,69392 | 25,38438 |
| 142 | 15,01921 | 15,36916 | 15,98154 | 17,28434 | 19,30599 | 21,66732 | 22,87709 | 23,65981 | 24,77289 | 25,47121 |
| 143 | 15,07813 | 15,42627 | 16,03585 | 17,33431 | 19,35413 | 21,72193 | 22,93896 | 23,72788 | 24,85183 | 25,55821 |
| 144 | 15,13796 | 15,48426 | 16,09095 | 17,38485 | 19,40239 | 21,77628 | 23,00054 | 23,7957 | 24,93071 | 25,64535 |
| 145 | 15,19865 | 15,54307 | 16,1468 | 17,43591 | 19,45078 | 21,8304 | 23,06182 | 23,86326 | 25,00951 | 25,7326 |
| 146 | 15,26014 | 15,60265 | 16,20336 | 17,4875 | 19,49929 | 21,88426 | 23,12279 | 23,93055 | 25,0882 | 25,81994 |
| 147 | 15,32236 | 15,66293 | 16,26058 | 17,53957 | 19,54794 | 21,93789 | 23,18344 | 23,99755 | 25,16676 | 25,90733 |
| 148 | 15,38523 | 15,72387 | 16,3184 | 17,59211 | 19,59671 | 21,99128 | 23,24379 | 24,06425 | 25,24518 | 25,99475 |
| 149 | 15,44869 | 15,78538 | 16,37679 | 17,64509 | 19,64562 | 22,04444 | 23,30382 | 24,13065 | 25,32342 | 26,08217 |
| 150 | 15,51268 | 15,84743 | 16,43569 | 17,69849 | 19,69465 | 22,09737 | 23,36352 | 24,19673 | 25,40146 | 26,16954 |
| 151 | 15,57713 | 15,90994 | 16,49506 | 17,75227 | 19,74381 | 22,15008 | 23,4229 | 24,26248 | 25,47927 | 26,25684 |
| 152 | 15,64199 | 15,97288 | 16,55485 | 17,80643 | 19,7931 | 22,20257 | 23,48196 | 24,32789 | 25,55683 | 26,34403 |
| 153 | 15,70719 | 16,03618 | 16,61503 | 17,86093 | 19,84253 | 22,25486 | 23,54069 | 24,39294 | 25,63412 | 26,43107 |
| 154 | 15,77268 | 16,0998 | 16,67555 | 17,91576 | 19,89208 | 22,30694 | 23,59909 | 24,45764 | 25,71109 | 26,51791 |
| 155 | 15,83841 | 16,16368 | 16,73636 | 17,97088 | 19,94176 | 22,35883 | 23,65715 | 24,52196 | 25,78773 | 26,60453 |
| 156 | 15,90433 | 16,22778 | 16,79744 | 18,02629 | 19,99157 | 22,41053 | 23,71489 | 24,58589 | 25,86401 | 26,69087 |
| 157 | 15,97037 | 16,29205 | 16,85873 | 18,08194 | 20,04151 | 22,46205 | 23,7723 | 24,64943 | 25,93988 | 26,77687 |
| 158 | 16,03642 | 16,35637 | 16,92016 | 18,13781 | 20,09159 | 22,5134 | 23,82936 | 24,71255 | 26,01527 | 26,86242 |
| 159 | 16,10238 | 16,42066 | 16,98164 | 18,19383 | 20,1418 | 22,56459 | 23,88608 | 24,7752 | 26,09009 | 26,9474 |
| 160 | 16,16812 | 16,48482 | 17,04309 | 18,24998 | 20,19214 | 22,61565 | 23,94245 | 24,83737 | 26,16425 | 27,03165 |
| 161 | 16,23355 | 16,54873 | 17,10442 | 18,30621 | 20,24261 | 22,66658 | 23,99846 | 24,89901 | 26,23767 | 27,11503 |
| 162 | 16,29856 | 16,61232 | 17,16557 | 18,36246 | 20,29322 | 22,7174 | 24,05411 | 24,9601 | 26,31025 | 27,19742 |
| 163 | 16,36304 | 16,67548 | 17,22646 | 18,41871 | 20,34396 | 22,76812 | 24,1094 | 25,02062 | 26,38191 | 27,27866 |
| 164 | 16,4269 | 16,73813 | 17,28701 | 18,4749 | 20,39484 | 22,81878 | 24,16434 | 25,08054 | 26,45257 | 27,3586 |
| 165 | 16,49004 | 16,80018 | 17,34714 | 18,531 | 20,44585 | 22,86939 | 24,21893 | 25,13984 | 26,52214 | 27,43711 |
| 166 | 16,55236 | 16,86154 | 17,4068 | 18,58697 | 20,497 | 22,91996 | 24,27317 | 25,19851 | 26,59053 | 27,51405 |
| 167 | 16,61378 | 16,92213 | 17,4659 | 18,64275 | 20,54829 | 22,97053 | 24,32708 | 25,25653 | 26,65768 | 27,58927 |
| 168 | 16,67419 | 16,98186 | 17,52437 | 18,69832 | 20,59971 | 23,02112 | 24,38067 | 25,31389 | 26,72351 | 27,66263 |
| 169 | 16,73352 | 17,04066 | 17,58215 | 18,75364 | 20,65127 | 23,07174 | 24,43395 | 25,37058 | 26,78794 | 27,734 |
| 170 | 16,79172 | 17,09848 | 17,63921 | 18,80869 | 20,70297 | 23,1224 | 24,48688 | 25,42653 | 26,85085 | 27,8032 |
| 171 | 16,84874 | 17,15529 | 17,69552 | 18,86345 | 20,75481 | 23,17309 | 24,53945 | 25,48171 | 26,91211 | 27,87002 |
| 172 | 16,90456 | 17,21105 | 17,75106 | 18,91791 | 20,80678 | 23,2238 | 24,59162 | 25,53603 | 26,97161 | 27,93429 |
| 173 | 16,95914 | 17,26574 | 17,8058 | 18,97206 | 20,85889 | 23,27453 | 24,64336 | 25,58946 | 27,02923 | 27,99583 |
| 174 | 17,01242 | 17,31932 | 17,85971 | 19,02588 | 20,91113 | 23,32526 | 24,69465 | 25,64194 | 27,08484 | 28,05447 |
| 175 | 17,06437 | 17,37174 | 17,91275 | 19,07936 | 20,9635 | 23,37599 | 24,74546 | 25,69341 | 27,13836 | 28,11004 |
| 176 | 17,11493 | 17,42296 | 17,9649 | 19,13248 | 21,01601 | 23,42672 | 24,79576 | 25,74384 | 27,18968 | 28,16242 |
| 177 | 17,16406 | 17,47294 | 18,01612 | 19,18523 | 21,06865 | 23,47745 | 24,84553 | 25,79318 | 27,23871 | 28,21147 |
| 178 | 17,21169 | 17,52163 | 18,06638 | 19,23759 | 21,12142 | 23,52815 | 24,89476 | 25,8414 | 27,28539 | 28,25708 |
| 179 | 17,25778 | 17,56898 | 18,11564 | 19,28955 | 21,17432 | 23,57884 | 24,94342 | 25,88845 | 27,32964 | 28,29916 |
| 180 | 17,30224 | 17,61492 | 18,16385 | 19,34108 | 21,22735 | 23,6295 | 24,99149 | 25,93431 | 27,37141 | 28,33763 |
| 181 | 17,34503 | 17,65943 | 18,21099 | 19,39217 | 21,28051 | 23,68014 | 25,03897 | 25,97897 | 27,41068 | 28,37249 |
| 182 | 17,38614 | 17,70249 | 18,25705 | 19,44283 | 21,3338 | 23,73076 | 25,0859 | 26,02248 | 27,44757 | 28,40389 |
| 183 | 17,4256 | 17,74412 | 18,30206 | 19,49308 | 21,38723 | 23,78138 | 25,13229 | 26,06491 | 27,48222 | 28,43204 |
| 184 | 17,46341 | 17,78435 | 18,34604 | 19,54292 | 21,4408 | 23,83201 | 25,1782 | 26,10633 | 27,51476 | 28,45716 |
| 185 | 17,49958 | 17,82317 | 18,38898 | 19,59236 | 21,49451 | 23,88266 | 25,22367 | 26,1468 | 27,54535 | 28,47948 |
| 186 | 17,5341 | 17,86058 | 18,43091 | 19,64143 | 21,54838 | 23,93334 | 25,26872 | 26,18641 | 27,57412 | 28,4992 |
| 187 | 17,56698 | 17,8966 | 18,47182 | 19,69013 | 21,60241 | 23,98407 | 25,3134 | 26,22521 | 27,60125 | 28,51656 |
| 188 | 17,5982 | 17,93121 | 18,51173 | 19,73847 | 21,65659 | 24,03485 | 25,35775 | 26,26329 | 27,62685 | 28,53179 |
| 189 | 17,62775 | 17,96442 | 18,55063 | 19,78645 | 21,71095 | 24,0857 | 25,40179 | 26,3007 | 27,65109 | 28,54509 |
| 190 | 17,65562 | 17,99621 | 18,58853 | 19,8341 | 21,76547 | 24,13662 | 25,44557 | 26,33751 | 27,67411 | 28,55668 |
| 191 | 17,68179 | 18,02657 | 18,62543 | 19,88141 | 21,82016 | 24,18763 | 25,48913 | 26,37378 | 27,69604 | 28,56678 |
| 192 | 17,70622 | 18,05548 | 18,66132 | 19,9284 | 21,87503 | 24,23873 | 25,53248 | 26,4096 | 27,71701 | 28,57558 |
| 193 | 17,7289 | 18,08295 | 18,69621 | 19,97507 | 21,93009 | 24,28993 | 25,57568 | 26,44501 | 27,73718 | 28,58329 |
| 194 | 17,74986 | 18,10898 | 18,73011 | 20,02144 | 21,98531 | 24,34124 | 25,61876 | 26,48012 | 27,75675 | 28,59021 |
| 195 | 17,76914 | 18,13363 | 18,76307 | 20,06752 | 22,04068 | 24,39265 | 25,66176 | 26,51501 | 27,77591 | 28,59665 |
| 196 | 17,78676 | 18,15692 | 18,79512 | 20,11333 | 22,0962 | 24,44416 | 25,70475 | 26,5498 | 27,79487 | 28,60286 |
| 197 | 17,80278 | 18,17891 | 18,82629 | 20,15888 | 22,15183 | 24,49577 | 25,74774 | 26,58454 | 27,81378 | 28,6091 |
| 198 | 17,81724 | 18,19963 | 18,85663 | 20,20419 | 22,20757 | 24,54747 | 25,79077 | 26,61933 | 27,83281 | 28,61558 |
| 199 | 17,8302 | 18,21914 | 18,88618 | 20,24928 | 22,26341 | 24,59925 | 25,83388 | 26,65422 | 27,8521 | 28,62252 |
| 200 | 17,84171 | 18,2375 | 18,91498 | 20,29416 | 22,31932 | 24,65111 | 25,8771 | 26,68929 | 27,87177 | 28,63007 |
| 201 | 17,85183 | 18,25476 | 18,94309 | 20,33886 | 22,37529 | 24,70306 | 25,92046 | 26,72458 | 27,89195 | 28,63841 |
| 202 | 17,86064 | 18,27099 | 18,97056 | 20,3834 | 22,4313 | 24,75507 | 25,96397 | 26,76016 | 27,91273 | 28,64767 |
| 203 | 17,86822 | 18,28626 | 18,99745 | 20,42781 | 22,48735 | 24,80714 | 26,00765 | 26,79607 | 27,93421 | 28,65798 |


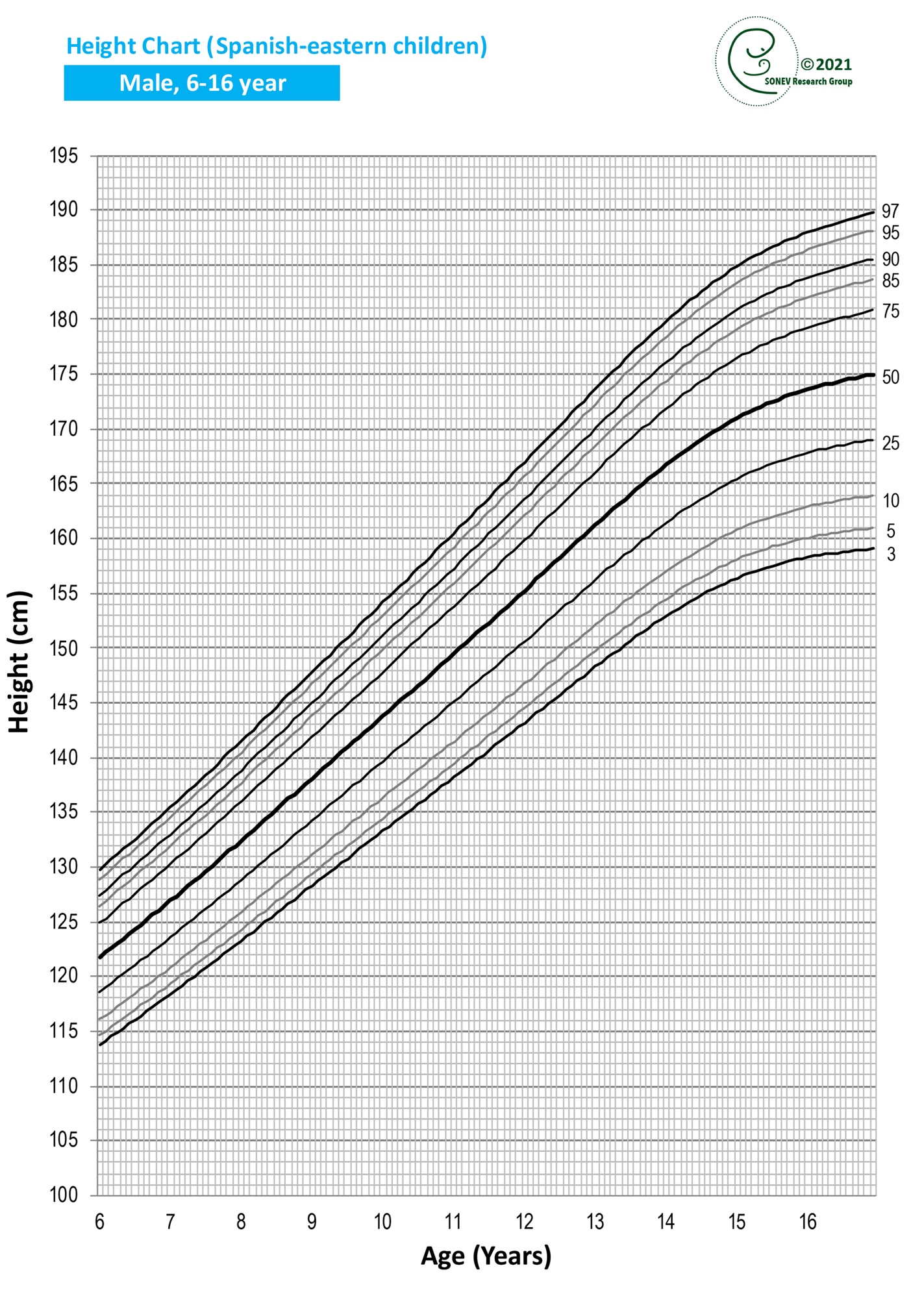


**Supplementary Figure 1. Spanish-eastern children male height chart.**


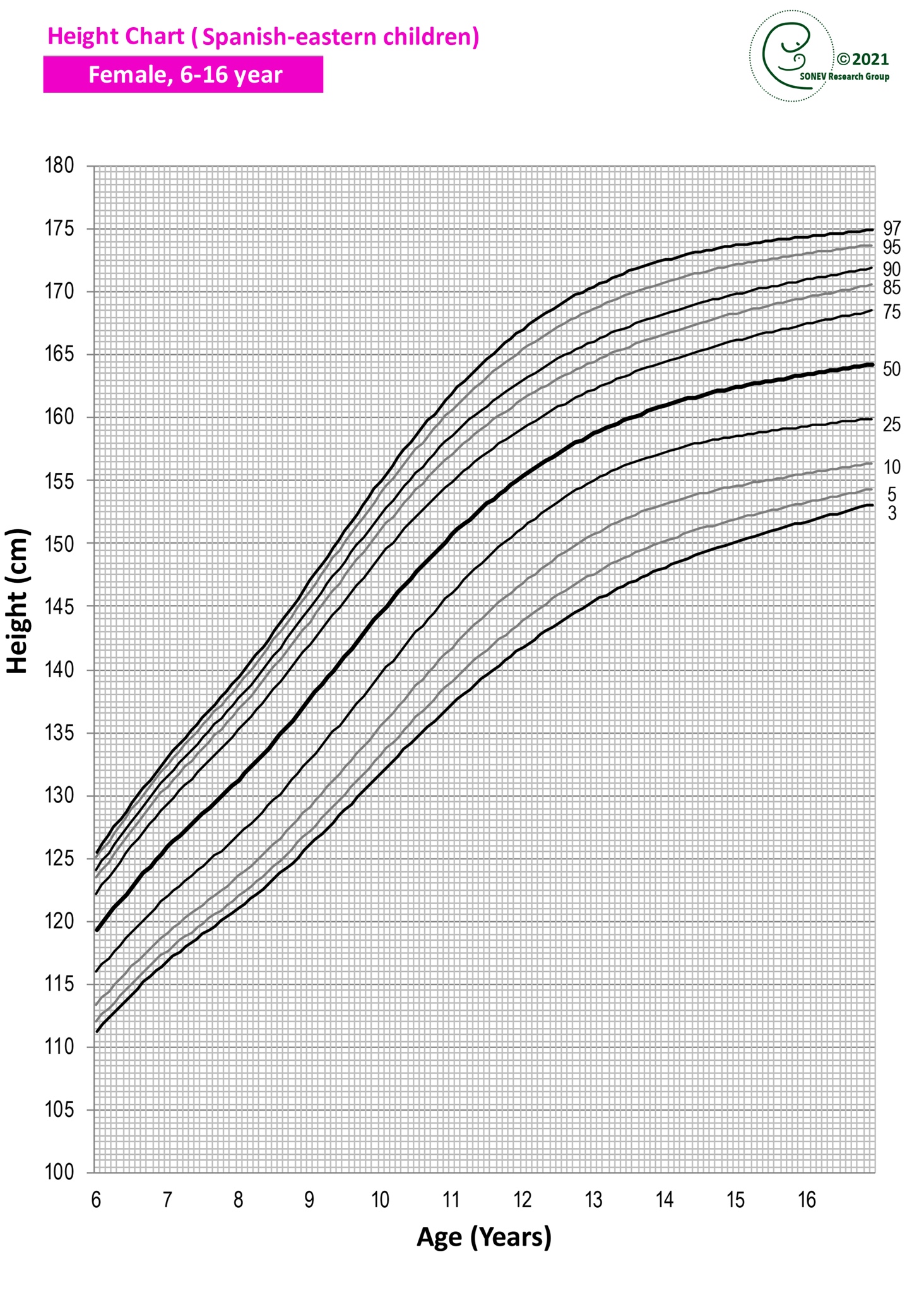
 **Supplementary Figure 2. Spanish-eastern children female height chart.**


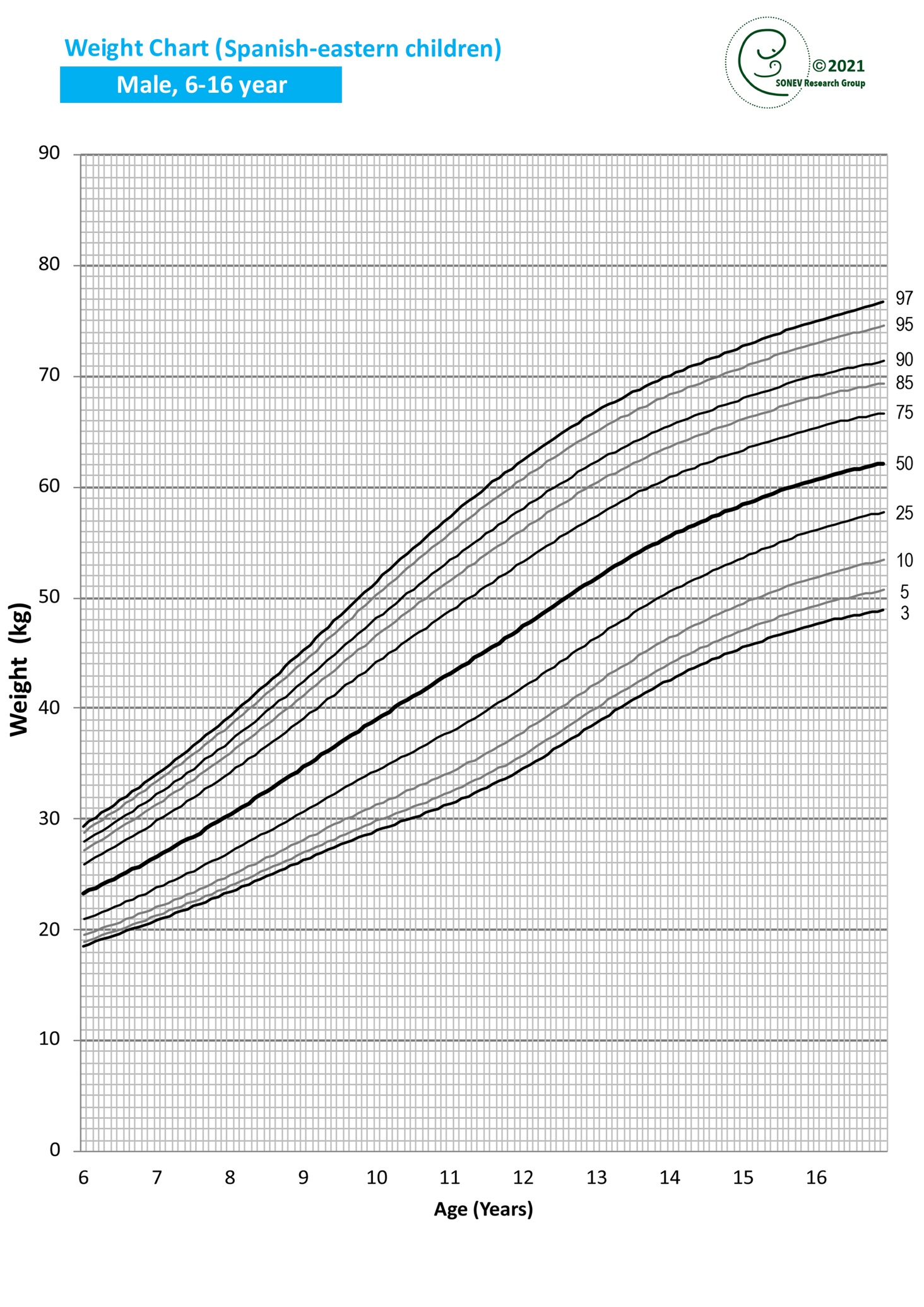
 **Supplementary Figure 3. Spanish-eastern children male weight chart.**


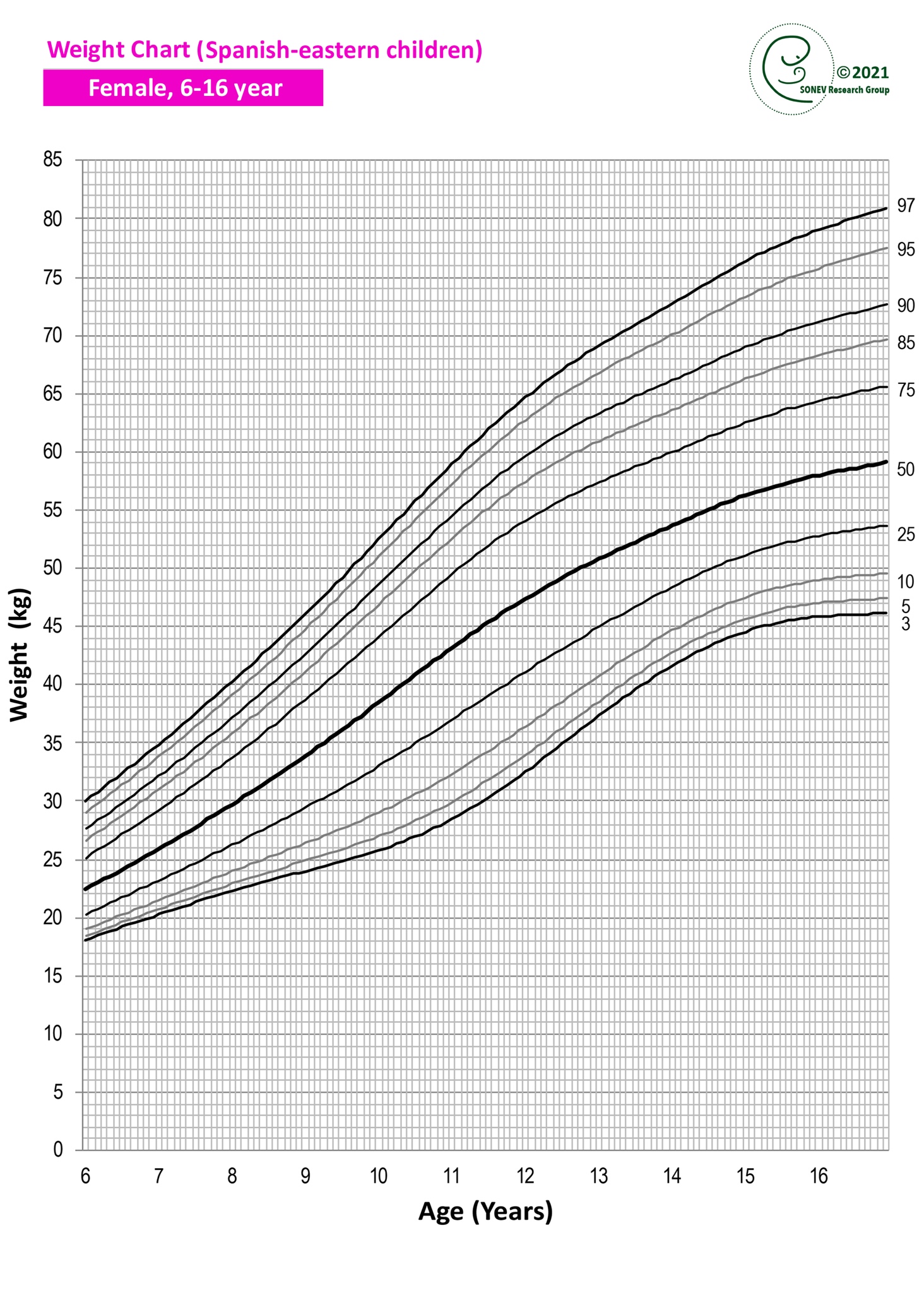


**Supplementary Figure 4. Spanish-eastern children female weight chart.**


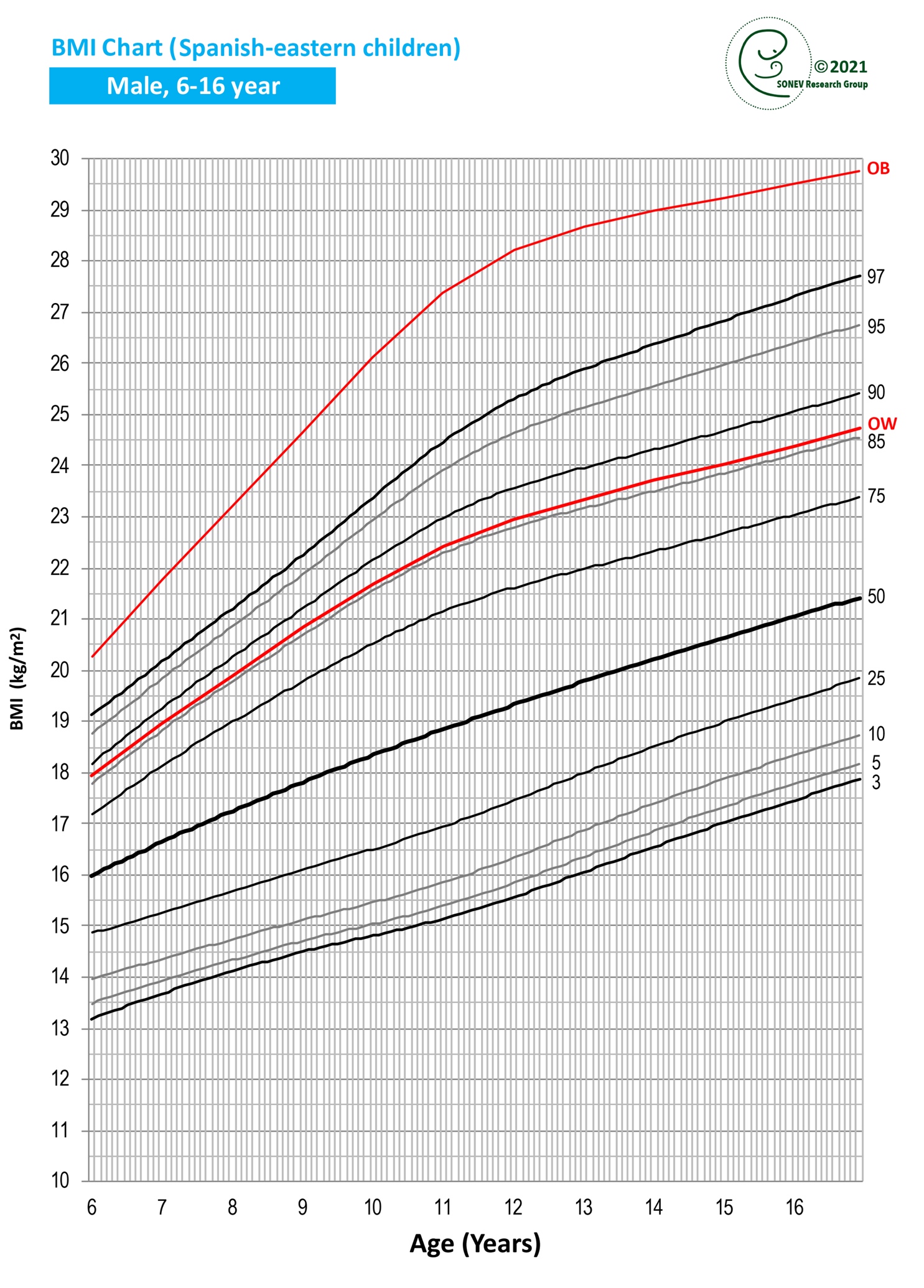


**Supplementary Figure 5. Spanish-eastern children male BMI chart.**


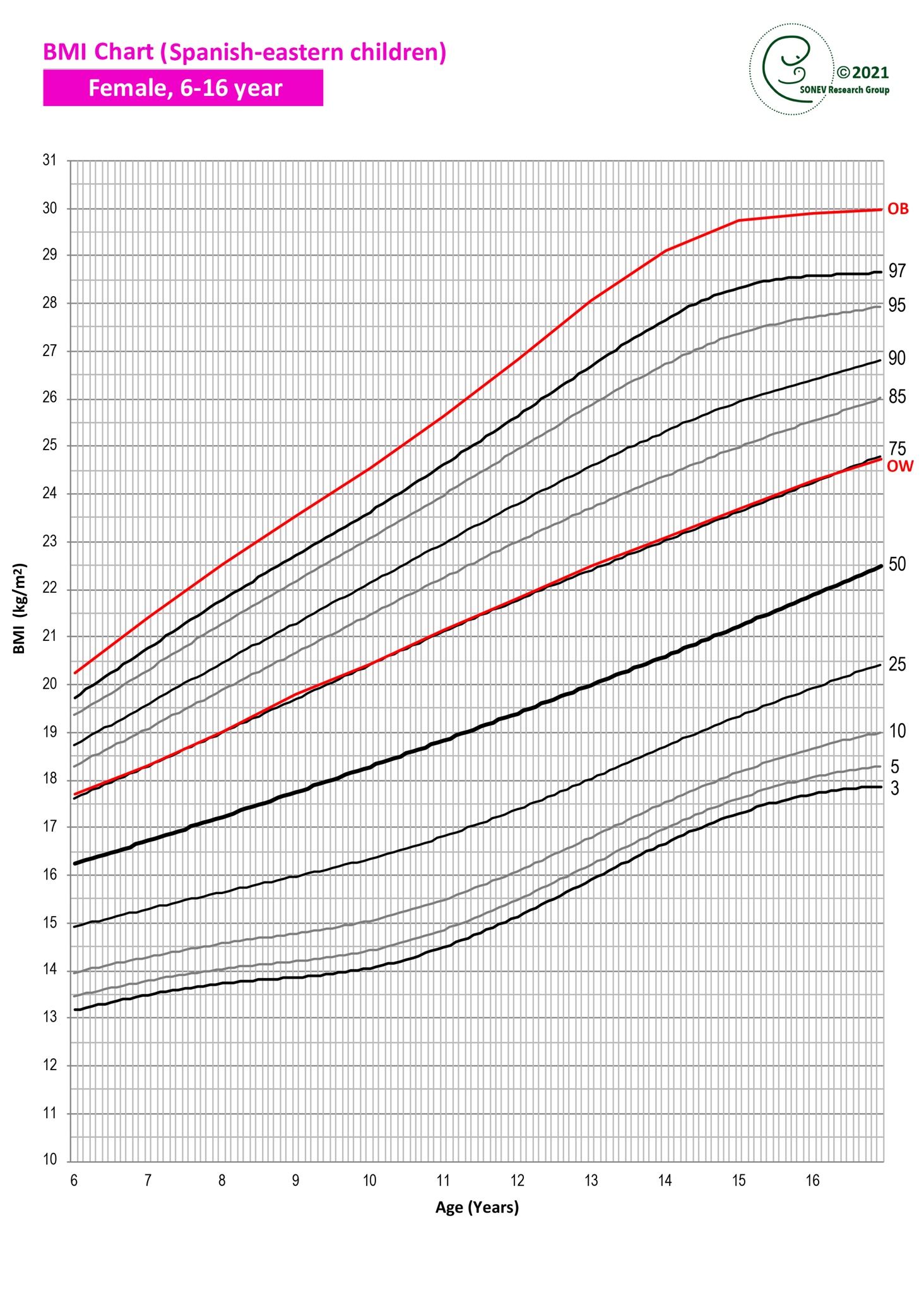


**Supplementary Figure 6. Spanish-eastern children female BMI chart.**
